# Supplementary material for: Selumetinib in combination with dexamethasone for the treatment of relapsed/refractory RAS-pathway mutated paediatric and adult acute lymphoblastic leukaemia (SeluDex): study protocol for an international, parallel-group, dose-finding with expansion phase I/II trial
Source: BMJ Open. 2022 Mar 4;12(3):e059872. doi: 10.1136/bmjopen-2021-059872 (PMC8900053; doi:10.1136/bmjopen-2021-059872)
Supplement: Author's manuscript [file bmjopen-2021-059872.draft_revisions.pdf]

BMJ Open is committed to open peer review. As part of this commitment we make the peer review history of every article we publish publicly available.

When an article is published we post the peer reviewers' comments and the authors' responses online. We also post the versions of the paper that were used during peer review. These are the versions that the peer review comments apply to.

The versions of the paper that follow are the versions that were submitted during the peer review process. They are not the versions of record or the final published versions. They should not be cited or distributed as the published version of this manuscript.

BMJ Open is an open access journal and the full, final, typeset and author-corrected version of record of the manuscript is available on our site with no access controls, subscription charges or pay-per-view fees (http://bmjopen.bmj.com).

If you have any questions on BMJ Open's open peer review process please email info.bmjopen@bmj.com

BMJ Open

Selumetinib in combination with dexamethasone for the treatment of relapsed/refractory RAS-pathway mutated paediatric and adult acute lymphoblastic leukaemia (SeluDex): study protocol for an international, parallel-group, dose-finding with expansion phase I/II trial

Journal:	*BMJ Open*
Manuscript ID	bmjopen-2021-059872
Article Type:	Protocol
Date Submitted by the Author:	03-Dec-2021

Complete List of Authors:	Menne, Tobias; Northern Center for Cancer Care<br>Slade, Daniel; University of Birmingham Cancer Research UK Clinical Trials Unit<br>Savage, Joshua; University of Birmingham Cancer Research UK Clinical Trials Unit<br>Johnson, Sarah; University of Birmingham Cancer Research UK Clinical Trials Unit<br>Irving, Julie; Newcastle University<br>Kearns, Pamela; University of Birmingham Cancer Research UK Clinical Trials Unit<br>Plummer, Ruth; Northern Center for Cancer Care; Newcastle University<br>Shenton, Geoff; Great North Children's Hospital<br>Veal, Gareth J; Newcastle University<br>Vormoor, Britta; Princess Maxima Center for Pediatric Oncology<br>Vormoor, Josef; Princess Maxima Center for Pediatric Oncology; Newcastle University<br>Billingham, Lucinda; University of Birmingham Cancer Research UK Clinical Trials Unit

Keywords:	Clinical trials < THERAPEUTICS, Leukaemia < ONCOLOGY, Adult oncology < ONCOLOGY, Paediatric oncology < ONCOLOGY

SCHOLARONE™
Manuscripts

Selumetinib in combination with dexamethasone for the treatment of relapsed/refractory RAS-pathway mutated paediatric and adult acute lymphoblastic leukaemia (SeluDex): study protocol for an international, parallel-group, dose-finding with expansion phase I/II trial

Tobias Menne[1]*, Daniel Slade[2]*, Joshua Savage[2]*, Sarah Johnson[2], Julie Irving[3], Pamela R. Kearns[2], Ruth Plummer[1,3], Geoff Shenton[4], Gareth J. Veal[3], Britta Vormoor[5], Josef Vormoor[3,5,6#], Lucinda Billingham[1#†]

* These authors have contributed equally and should be considered joint first authors.

# These authors have contributed equally and should be considered joint senior authors.

† Corresponding author contact details:

Prof. Lucinda Billingham, Cancer Research UK Clinical Trials Unit (CRCTU), Institute of Cancer and Genomic Sciences, University of Birmingham, Edgbaston, Birmingham. B15 2TT. UK. Email: L.J.Billingham@bham.ac.uk

[1] Northern Centre for Cancer Care, Freeman Hospital, Newcastle upon Tyne Hospitals NHS Foundation Trust, Newcastle upon Tyne. NE7 7DN. UK

[2] Cancer Research UK Clinical Trials Unit (CRCTU), Institute of Cancer and Genomic Sciences, University of Birmingham, Edgbaston, Birmingham. B15 2TT. UK

[3] Wolfson Childhood Cancer Research Centre, Newcastle University Centre for Cancer, Newcastle University, Newcastle upon Tyne. NE1 7RU. UK

[4] Great North Children's Hospital, Royal Victoria Infirmary Hospital, Newcastle upon Tyne Hospitals NHS Foundation Trust, Newcastle upon Tyne. NE7 7DN. UK

[5] Princess Máxima Center for Paediatric Oncology, Heidelberglaan 25, 3584CS Utrecht. The Netherlands

[6] University Medical Center, Utrecht, Heidelberglaan 100, 3584CX Utrecht. The Netherlands

For peer review only

Abstract

Introduction

Event free survival rates at 15 years for paediatric patients with relapsed/refractory acute lymphoblastic leukaemia (ALL) are 30-50%, with 5-year survival for adult patients only 20%. Many patients with newly diagnosed and relapsed ALL harbour somatic RAS-signalling activation mutations. Induction therapy for ALL involves steroids, with preclinical data suggesting the combination of dexamethasone with the MEK1/2 inhibitor, selumetinib (ARRY-142886) has a synergistic anti-cancer effect.

Methods and analysis

The SeluDex trial is an international, parallel-group, dose-finding with expansion, phase I/II trial to assess the selumetinib/dexamethasone combination in adult and paediatric patients with relapsed/refractory, RAS pathway mutant ALL. The Cancer Research UK Clinical Trials Unit at University of Birmingham is the UK Coordinating Centre, with national hubs in Copenhagen, Denmark; Monza, Italy; Münster, Germany; Paris, France; and Utrecht, Netherlands. Patients with morphologically proven relapsed/refractory or progressive B-cell precursor or T-ALL, with demonstrated RAS pathway activating mutations are eligible. Adult patients are $\geq$18 years old, ECOG $\leq$2 and paediatric <18 years old, Lansky play scale ≥60% or Karnofsky score ≥60%. Phase I primary objective is the recommended phase II dose of selumetinib as defined by occurrence/non-occurrence of dose limiting toxicities using the continual reassessment method; phase II will evaluate preliminary anti-leukaemic activity of the combination, as defined by morphological response 28 days post treatment using a Bayesian approach. Target recruitment is between 26-42 patients (minimum 13 and maximum 21 per group), depending the number of phase I patients included in phase II.

Ethics and dissemination

Medical ethical committees of all the participating countries will approve the study protocol. Results will be disseminated through national and international presentations and peer-reviewed publications.

Trial registration number

ISRCTN: 92323261

Strengths and limitations of this study

- Novel combination of the MEK1/2 inhibitor, selumetinib, with dexamethasone
- Seamless phase I/II Bayesian trial design with a Continual Reassessment Method for dose escalation in phase I
- Parallel cohort trial design of adult and paediatric patients within one protocol
- Availability of CAR T-cell therapy since this trial started recruitment has competed for the same patient population
- Offers a bridging treatment option for patients awaiting CAR T-cell therapy outside clinical studies or at relapse after CAR-T treatment

For peer review only

Introduction

Acute lymphoblastic leukaemia (ALL) is the most common childhood cancer, representing 26.8% of all childhood malignancies (1). While the overall cure rate for newly diagnosed paediatric ALL is approaching 90%, children with relapsed ALL (rALL) are still facing a poor outlook, with reported overall event-free survival rates at 15 years of 30-50% (2), and rALL remains a frequent cause of death.

In adults, the frequency of ALL is significantly lower, with an incidence of around one per 100,000 (3). ALL in adulthood has proven to be more challenging to treat compared to childhood ALL, with the disease being more resistant to chemotherapy, and patients have a reduced treatment tolerance especially in the elderly population. Five-year overall survival rate remains at ~20% among adult patients aged ≥60 years, even though improvement has been observed since 1980 (4).

Data demonstrate that mutations activating the RAS-signalling cascade (NRAS, KRAS, FLT3 and PTPN11 and cCBL) are highly prevalent at both diagnosis and relapse of paediatric B-cell precursor ALL (incidence 38% at relapse), and are associated with high-risk features such as early relapse, central nervous system (CNS) involvement, and chemoresistance (5). Neurofibromin (NF1) copy number variants, point mutations or intragenic deletions have been reported in approximately 4% of T-cell ALL, 14% of early T-cell precursor (ETP), ~30% of low hypodiploidy, 2% high risk of B-cell ALL and 75% of relapsed hypodiploid ALL (6-10). BRAF point mutation incidence is approximately 3% in T-cell ALL, 4% in ETP and 0.5% in high-risk B-cell ALL (7-9). IKZF2 and IKZF3 deletions which have recently been shown to activate the RAS-pathway have an incidence of approximately 18% in hypodiploid ALL (11). IL7Rα point mutations, deletions and in-frame alterations are found in approximately 9% of T-ALL (12, 13), and JAK1 point mutations are found in approximately 5% of T-ALL (14).

Selumetinib is a potent and selective allosteric MEK1/2 inhibitor that has been evaluated in phase II/III clinical trials for several cancers, including BRAF mutation-positive melanoma (15), metastatic uveal melanoma (16), pancreatic (17), colorectal (18), and KRAS mutation-positive NSCLC (19). Pre-clinical models suggest a synergistic effect when selumetinib and dexamethasone were combined in an orthotopic mouse model with three different RAS-pathway mutant ALL primagrafts and a mechanistic basis to the synergy was identified involving heightened levels of the proapoptotic Bim protein (20). However, the combination of selumetinib and dexamethasone has not been evaluated in a clinical trial setting.

The aim of the SeluDex trial is to determine the dose of selumetinib to use in combination with dexamethasone in adult and paediatric patient populations, and to assess the preliminary anti-leukaemic properties of the combination in each group of patients.

Methods and analysis

Study design

The SeluDex trial is a parallel-group, non-randomised, open-label, dose-finding with expansion, phase I/II clinical trial. As an early phase dose-finding and signal-seeking trial, a comparator arm was not included. Group A (adult patients) and Group P (paediatric patients) will both receive selumetinib in combination with a standard dose of dexamethasone (Figure 1). Patients will be recruited from public hospitals. The Cancer Research UK Clinical Trials Unit (CRCTU) at University of Birmingham is the UK Coordinating Centre, with national hubs in Copenhagen, Denmark; Monza, Italy; Münster, Germany; Paris, France; and Utrecht, Netherlands. The national coordinating and participating national centres are all part of the Innovative Therapies for Children with Cancer in Europe consortium (ITCC).

For peer review only

This trial aims to recruit between 26 and 42 patients (minimum of 13 and maximum of 21 in each group), depending on how many phase I patients are included also in phase II. The recruitment period is expected to be four years, with participants followed up for one month after a maximum of 6 treatment cycles. The World Health Organization (WHO) Trial Registration Data Set is attached in Supplementary Appendix 2.

Patient and public involvement

Patients and public were not involved in the design and conduct of this research but will be consulted in the reporting and dissemination of its outputs.

Patient selection

The two parallel patient groups in SeluDex, Group A (Adult) and P (Paediatric), have similar but discrete eligibility criteria. The main eligibility criteria are detailed in Tables 1 and 2, respectively. The screening process is the same for both groups. Eligibility for trial entry is dependent upon screening of a bone marrow aspirate to confirm morphological relapse and RAS-pathway mutation status. Peripheral blood may also be used in the case of a dry tap if white blood cell counts >50 x 10^9/L. Centralised genetic analysis in the UK at Northern Genetics Service's laboratories in Newcastle upon Tyne will identify the presence of a RAS-pathway activating mutation (key exons of NRAS, KRAS, FLT3, PTPN11 or cCBL mutation). A primary screen of the two most common mutations (NRAS and KRAS) will be performed first and if no mutations are found a secondary screen of the remaining mutations (FLT3, PTPN11 and cCBL) will be performed. Alternatively, locally obtained genetic results may be used to confer RAS-pathway mutation status if tested in a clinically compliant laboratory using a clinically validated test with material from the current relapse. Laboratories will be approved by the Trial Office to utilise local NRAS, KRAS, FLT3, PTPN11, cCBL, NF1, BRAF, IKZF2, IKZF3, IL7Rα or JAK1 testing.

Consent

Consent is requested by an investigator at two time points. A pre-trial genetic assessment consent allows for central/local analysis of a sample at screening to assess RAS mutation status, and the main trial consent, which is performed once mutation status is confirmed and a treatment slot is reserved. The main trial consent will be for either the phase I dose finding, of phase II dose expansion. Furthermore, age-specific consent/assent forms are available for patients over 16 years of age, and parents/legal guardians of paediatric patients (as appropriate according to age and national legislation). An example of the main consent form for adult patients is available in Supplementary Appendix 2. Age-specific patient information sheets are available for patients over 16 years of age, parents/legal guardians of paediatric patients, and teenage patients aged 13-15 years. As an example, the adult patients information sheet is available in Supplementary Appendix 3.

Interventions

The dosing schedule for the dose-finding phase of the trial is summarised below for each group and detailed in relevant tables. Treatments are given over a 28-day cycle and will be continued for six cycles unless one or more of the following is observed; intolerable toxicity, confirmed disease recurrence or progression, pregnancy, severe non-compliance to protocol, development of any trial-specific criteria for discontinuation (not reaching at least a partial response (PR) at Cycle 1 day 28), or investigator decision, for example, if the patient requires a prohibited concomitant medication. The trial schedule of events is included in Supplementary Appendix 4.

For peer review only - http://bmjopen.bmj.com/site/about/guidelines.xhtml

Group A (Table 3)

Dexamethasone: Steroids are a backbone of all induction blocks of ALL therapy with dexamethasone, the preferred steroid in the management of patients with ALL. Initially, dexamethasone was to be administered on a continuous dosing schedule with a weekly total dose of 42mg/m^2 for the first course. However, following an observed increase in the risk of infections during the initial stages of the trial, the trial dexamethasone dosing regimen was amended to allow intermittent treatment in blocks rather than continuously, and incorporating a dose reduction from 6mg/m^2/day in cycle 1 (D2-4 & D8-11) to 4mg/m^2/day during D15-18 and D22-25 of cycle 1. Further dexamethasone is given as a low dose in cycle 2 (D1-4 only: 4mg/m2/day) and then as a high dose again in subsequent cycles (C3-6, D1-5 only: 6mg/m^2/day).

Selumetinib: The dose of 75mg was chosen as the starting dose for Group A (Dose Level 0) with the dose only increased if pharmacokinetic (PK) data suggest the addition of dexamethasone decreases selumetinib serum concentrations significantly due to induction of the cytochrome P450 enzyme CYP3A4 (21).

Group P (Table 4)

Dexamethasone: As with adult dosing, trial dexamethasone dosing regimen was amended during the initial stages of the trial to be reduced after the first two weeks and to be given pulsed during cycle 1 due to the increase in infection risk.

Selumetinib: Results of two paediatric phase I trials using selumetinib only (22, 23) gave the maximum tolerated dose (MTD) as 2 x 25mg/m^2; thus in the SeluDex trial, 20mg/m^2 (80% of the single agent MTD of selumetinib) was chosen as the starting dose for safety reasons (Dose Level -1), specifically for those patients with CNS disease receiving concomitant intrathecal methotrexate.

Treatment compliance

Treatment compliance should be discussed with the patient/parent/legal guardian at the beginning of each cycle when study drug is dispensed. The research nurse should complete the relevant sections including recording the morning and afternoon doses and the dates they are to be taken on the diary. All patients/parents/legal guardians will be required to complete a diary with the daily time of administration of the study drugs, which must be returned to the clinic for checking at each visit. If a dose is missed, the reason must be noted in the diary by the patient/parent/legal guardian. Patients/parents/legal guardians should be advised to return any unused investigational medcicinal product in the original bottles, in addition to returning any empty bottles. Compliance should be reviewed at the end of each cycle by the site staff via review of the patient diary and discussion with the patient/parent/legal guardian that is to be documented in the medical notes.

Dose modifications

No dexamethasone dose reductions are permitted during cycle 1 and 2. If identifiable dexamethasone related intolerable toxicity is present during cycle 3 then a dose reduction to 4mg/m^2/day is allowed from cycle 4 onwards.

Dose modifications for the following selumetinib-related toxicities are allowed:

- Grade 3 neutropenia with infection and/or fever (GCSF is permitted)

For peer review only - http://bmjopen.bmj.com/site/about/guidelines.xhtml

- Grade 3 or 4 nausea, vomiting, or diarrhoea, if persistent despite optimal antiemetic and/or anti-diarrhoeal therapy
- Any Grade 3 or greater non-haematological toxicity
- Any other Grade 4 haematological toxicity

If one or more of these are observed the following action should be taken;

1st occurrence: Hold selumetinib until recovery to Grade <1 or baseline; restart at original dose level

2nd occurrence: Hold selumetinib until recovery to Grade <1 or baseline; keep same dose but reduce to once daily

3rd occurrence: Hold selumetinib until recovery to Grade <1 or baseline; use 60% of the original dose but go back to twice daily. For group P: 45mg reduce to 30 mg; 40 mg to 25 mg; 30 – 35 mg to 20 mg; 20 – 25 mg to 10 mg. For Group A: 75 mg reduce to 45 mg; 60 mg to 35 mg

4th occurrence: Discontinue selumetinib

Concomitant medication

Patients should avoid consuming large amounts of grapefruits, seville oranges, and any other products that may contain these fruits, e.g., grapefruit juice. In addition, patients should not take any vitamin E supplements. Restricted medications are listed in in Table 5.

Trial outcomes

Phase I

The primary objective for the phase I dose-finding component is to determine the recommended phase II dose (RP2D) of selumetinib in combination with dexamethasone, as defined by the occurrence/non-occurrence of dose limiting toxicities (DLTs) in the trial-defined assessment period. The trial will achieve this through the use of the continual reassessment method (CRM) (24). DLTs will be assessed from the first dose on cycle 1, day 1 up until cycle 1, day 28 during phase I only. A DLT is defined as any toxicity which is dose limiting, is not attributable to the disease or disease-related processes under investigation, and is considered at least possibly related to selumetinib or dexamethasone (see Dose Limiting Toxicity section).

Secondary outcomes for the phase I component are:

- The occurrence of adverse events (AEs) as measured by Common Terminology Criteria for Adverse Events (CTCAE) version 4 (25) and causality assessment
- Pharmacokinetic variables of selumetinib in combination with dexamethasone from the concentration time profile (area under the plasma concentration-time curve (AUC), C_{max}, T_{max}, $t_{1/2}$)
- Response to treatment assessed by complete remission rate at 28 days as measured by morphological and minimal residual disease (MRD) response in bone marrow (BM) and for patients with CNS involvement only clearance of cerebrospinal fluid (CSF) blasts at 28 days
- Difference in pharmacokinetics of selumetinib (ΔAUC) when selumetinib is administered as single agent and in combination with dexamethasone

For peer review only - http://bmjopen.bmj.com/site/about/guidelines.xhtml

Phase II

The primary objective for the phase II expansion component will be to assess preliminary anti-leukaemic activity of the selumetinib/dexamethasone drug combination as defined by morphological response (complete remission (CR), complete remission with incomplete platelet recovery (CRi), partial remission (PR), non-response (NR) and for patients with CNS involvement only, clearance of CSF blasts) 28 days post-treatment end. Response evaluation will be undertaken with bone marrow response at day 28, including MRD and CSF surveillance for CNS positive patients with standard cytology. A patient will be classified as having a response if they achieve CR or CRi at the day 28 assessment (with an acceptable window of day 15 to day 35).

Secondary outcomes for the phase II component are;

- The occurrence of AEs as measured by CTCAE version 4 and causality assessment
- The occurrence/non-occurrence of DLTs in the trial defined assessment period
- Pharmacokinetic variables of selumetinib in combination with dexamethasone from the concentration time profile (AUC, C_{max}, T_{max}, $t_{1/2}$)
- Difference in pharmacokinetics of selumetinib (ΔAUC) when selumetinib is administered as single agent and in combination with dexamethasone
- MRD response in bone marrow at 28 days

Exploratory outcomes of the trial for both phases of the trial include exploratory pharmacodynamic (PD) biomarker studies if clinical responses are observed. These will include levels of phosphorylated ERK by flow cytometry in leukaemic cells from patients at several time points (cycle 1 day 1, cycle 1 day 4 and end of treatment visit), as well as retrospective mRNA profiling, including the apoptotic initiator BIM (20, 26).

Dose limiting toxicities

DLTs will be assessed from the first dose on cycle 1 day 1 up until cycle 1 day 28 during Phase I. A haematological DLT is defined as bone marrow aplasia/hypoplasia, defined as overall marrow cellularity less than 25% (malignant infiltration or other causes are excluded), which does not recover by cycle 1 day 28. This includes an absolute neutrophil count of less than $0.5x10^9/L$ and a non-transfusion dependent platelet count of less than $25x10^9/L$ due to bone marrow aplasia/hypoplasia. In addition, Grade 4 febrile neutropenia, and any Grade 5 AE are also defined as a haematological DLT.

Non-haematological DLTs in Group A are defined as Left Ventricular Ejection Fraction <45% as measured by echocardiogram; and in Group P as >15% reduction in Shortening Fraction from baseline by echocardiogram. Results must be viewed by a consultant cardiologist to confirm that there has been a genuine deterioration in cardiac function. In addition, any Grade 5 AE, Grade 3 nausea or vomiting on or before C1D28 only which does not resolve to <Grade 2 within 48 hours (with or without supportive care), Grade 3 mucositis on or before C1D28 only which does not resolve to <Grade 2 within 48 hours (with or without supportive care), any other Grade 3 or 4 non-haematological toxicity, specifically excluding: Alopecia; dexamethasone related CTCAE Grade 3 toxicities (e.g., hyperglycaemia, muscle weakness, psychosis, avascular necrosis); tumour lysis syndrome; and increased alanine transaminase or aspartate aminotransferase.

Suspected DLTs will be reviewed and confirmed by the TSC during dose decision meetings at the end of each cohort during Phase I.

For peer review only - http://bmjopen.bmj.com/site/about/guidelines.xhtml

Statistical analysis plan

Both groups (A and P) will be analysed separately for both phases of the trial. If the response rates in the two groups are seen to be similar in the phase II component of the trial, then there may be some consideration to analysing response in the adult and paediatric groups collectively. The phase I evaluable population includes those patients who do not withdraw/die or discontinue treatment due to non-treatment related causes before the end of the DLT assessment period. A patient is evaluable regardless of the above if they experience a DLT. The phase II evaluable population includes all patients who received any treatment. There is no scope to replace unevaluable patients in the trial.

Phase I

The maximum number of patients to be recruited in phase I is 24; 12 into each group in parallel.

The trial will utilise the continual reassessment method (CRM) to find which doses to take forward to the phase II activity component of the trial for each group respectively. Each group will use a modified two-stage Bayesian CRM (27), recruiting patients in cohorts of two. The design incorporates rules to enable early stopping if the DLT rate at the lowest dose is unacceptably high. Patients are assigned to the starting dose level and follow the initial design (see Tables 3 and 4) until the first DLT is observed. Once the first DLT is observed the CRM takes over and determines the 'best' dose level that the next cohort should be assigned to, based on the model-based predicted probabilities of toxicity at each possible dose. The acceptable level of toxicity (probability of a DLT) has been chosen to be 17% for each group, that is, the trial will aim to find the highest dose that coincides with this acceptable level. This value was selected based on discussion with clinicians concerning the acceptable rate of DLTs based on the chosen definition.

The stopping rule for toxicity utilised in the modified CRM is based on the probability of the true toxicity rate at the lowest dose being >27%. If based on the observed data the probability of the true rate of toxicity being greater than this chosen value is >0.85 then the modified CRM will suggest that the trial should stop. The current MTD estimate is calculated after each cohort has been assessed for the full DLT assessment period and the next cohort of patients are then assigned to this MTD estimate. The final MTD estimate calculated after all 12 patients have been treated is the dose estimate to be considered to be taken forward to the phase II component.

This design works relatively well with computer simulations showing a probability for correctly selecting the right dose level of at least 0.6 when it is truly dose level 0 or 1, around 0.5 when it is truly dose level -1 and at least 0.6 for stopping early when all doses are truly too toxic.

Data will be reviewed at each dose decision stage by the independent Trial Safety Committee (TSC). The TSC will incorporate PK results into the decision process; the CRM will advise as to which doses are safe, with the PK additionally informing if there is a need for escalation/de-escalation.

Phase II

The phase II part of the trial will include patients from the phase I component that were dosed at the RP2D plus patients recruited into the expansion cohort. The number of phase I patients taken forward to the phase II assessment will be at least 4 in each group and the maximum number of patients to be recruited in the expansion cohort will be 9 in each group, giving a total of 13.

Based on data published by the TACL group (CR rate of 44% for second relapses and 27% for third relapses (28)) and seeing as we expect a mixture of 2nd, 3rd and higher relapses in paediatric patients and similar if

For peer review only - http://bmjopen.bmj.com/site/about/guidelines.xhtml

not worse relapse rates for adult patients, we estimate the overall response rate on standard treatment to be approximately 35%.

This phase will utilise a Bayesian approach to investigate the true response rate in each group. A Beta-Binomial conjugate analysis will be used to create a posterior probability distribution of the true response rate using the observed trial data combined with a Beta (1,1) minimally informative prior.The trial design is based on the decision criteria that if there is a high probability (>0.80) that the true response rate is >35% then this will indicate that the treatment is worthy of further research. With 13 patients, this design works reasonably well with a low probability of 0.08 of incorrectly concluding that the treatment is worthy when the true response rate is only 25% and a high probability of 0.82 of correctly concluding that the treatment is worthy when the true response rate is 55%.

If the response rates are similar in both groups, then information may be borrowed across Groups A and P to provide more accuracy in determining the true response rate for both groups collectively

There are no planned subgroup or interim analyses.

Pharmacokinetic and pharmacodynamic samples

Blood samples for PK and PD analysis will be obtained on days one, two and four of cycle 1, with blood samples for PK analysis collected again on day one of cycle 2, and blood samples for PD analysis obtained at the end of trial treatment.

For PK analysis plasma will be subjected to liquid chromatography-mass spectrometry (LC-MS) analysis to quantification concentrations of selumetinib, its major metabolite N-desmethyl selumetinib and dexamethasone. Two types of PD samples will be prepared from peripheral blood samples of patients who have a white cell count greater than $10x10^9$/L; samples will either be fixed on site in BD Lyse Fix solution or collected separately in Blood RNA Preservative Tubes. These PD samples will be transferred to the UK Biobank at the University of Birmingham for storage.

All samples will be collected in accordance with national regulations and requirements including standard operating procedures for logistics and infrastructure. Samples will be taken in appropriately licensed premises, stored and transported in accordance with the Human Tissue Authority guidelines and NHS trust policies.

Adverse events reporting and analysis

The collection and reporting of AEs as measured by CTCAE v4 will be in accordance with the Research Governance Framework for Health and Social Care and the requirements of the National Research Ethics Service (NRES). Definitions of different types of AEs are listed in online Supplementary Appendix 5; this includes abnormal laboratory findings which are considered clinically significant. The reporting period for AEs will be from the date of commencement of protocol defined treatment until 28 days after the administration of the last treatment. The investigator should assess the seriousness and causality (relatedness) of all AEs experienced by the patient (this should be documented in the source data) with reference to the protocol. In addition, DLTs will be assessed for the Phase I part of the trial only. All will be reported using the applicable electronic case report form (eCRF).

For peer review only - http://bmjopen.bmj.com/site/about/guidelines.xhtml

Data management

Case report forms (CRFs) can be entered online at https://www.cancertrials.bham.ac.uk/SeluDexLive. Authorised staff at sites will require an individual secure login username and password to access this online data entry system. Paper CRFs must be completed, signed/dated and returned to the SeluDex Trial Office by the investigator or an authorised member of the site research team. Data reported on each CRF should be consistent with the source data or the discrepancies should be explained. If information is not known, this must be clearly indicated on the CRF. All missing and ambiguous data will be queried. All sections are to be completed.

All trial records must be archived and securely retained for at least 25 years. No documents will be destroyed without prior approval from the sponsor, via the SeluDex Trial Office. On-site monitoring will be carried out as required following a risk assessment and as documented in the Quality Management Plan. Any monitoring activities will be reported to the central SeluDex Trial Office and any issues noted will be followed up to resolution. SeluDex will also be centrally monitored, which may trigger additional on-site monitoring. Further information regarding data management is provided in the study protocol.

The CRCTU will hold the final trial dataset and will be responsible for the controlled sharing of anonymised clinical trial data with the wider research community to maximise potential patient benefit while protecting the privacy and confidentiality of trial participants. Data anonymised in compliance with the Information Commissioners Office requirements, using a procedure based on guidelines from the Medical Research Council (MRC) Methodology Hubs, will be available for sharing with researchers outside of the trials team within 12 months of the primary publication.

Trial organisation structure

The University of Birmingham will act as sponsor to this international, multi-centre study: Support Group, Aston Webb Building, Room 119, Birmingham, B15 2TT. Email: researchgovernance@contacts.bham.ac.uk. The trial is being conducted under the auspices of the CRCTU, The University of Birmingham according to their local procedures. The Trial Management Group (TMG) will be responsible for the day-to-day running and management of the trial. Members of the TMG include the chief investigator, coinvestigators, biological coordinators, trial management team leader, senior trial coordinator, trial coordinator, lead trial statistician and trial statistician. The TMG will have regular meetings during recruitment. The TSC will consist of independent clinicians, Dr Bela Wrench and Dr Christina Halsey, as well as an independent statistician, Dr Graham Wheeler.

Data analyses will be supplied in confidence to the independent TSC, which will be asked to support dose decisions and advise on whether the accumulated data from the trial, together with the results from other relevant research, justify the continuing recruitment of further patients. The TSC will operate in accordance with a trial specific charter based on the template created by the Damocles Group. The TSC will meet at the end of every cohort in phase I and at least every 6 months in phase II, or more often if required e.g., an emergency meeting may also be convened if a safety issue is identified. The TSC will report directly to both the SeluDex TMG who will convey the findings of the TSC to the funders/sponsor as appropriate or when specifically requested by these parties.

Confidentiality

Confidential information collected during the trial will be stored in accordance with the General Data Protection Regulation (GDPR) 2018. As specified in the patient information sheet (PIS) and with the

For peer review only

For peer review only - http://bmjopen.bmj.com/site/about/guidelines.xhtml

patient's consent, patients will be identified using only their initials, date of birth and unique trial ID number. Authorised staff may have access to the records for quality assurance and audit purposes. The Trials Office maintains the confidentiality of all patients' data and will not disclose information by which patients may be identified to any third party other than those directly involved in the treatment of the patient and organisations for which the patient has given explicit consent for data transfer (e.g., laboratory staff).

Trial registration

ISRCTN: 92323261. Date applied: 22-Jan-2018; date assigned: 23-May-2018. Due to administrative delays, the date assigned occurred five days after the first patient was recruited (18-May-2018).

EudraCT: 2016-003904-29. Retrospective registration by UK competent authority (MHRA): 27-Mar-2019.

ClinicalTrials.Gov: NCT03705507. Retrospective registration: 15-Oct-2018.

ITCC-063 study

Trial status

Recruitment for the trial opened in April 2018 and the first patient was recruited on 18-May-2018; recruitment is expected to last until 31-Jan-2023. A list of open sites can be obtained from the SeluDex Trial Office (seludex@trials.bham.ac.uk).

Discussion

Position of the trial in an era of CAR-T cell treatment

The SeluDex trial was initially proposed in 2013, before the advent of CAR-T cell and other CD19 and CD22 targeting immunotherapies. This trial was designed for patients who had relapsed after stem cell transplantation, with little or no other therapeutic options. The trial opened in the UK in Spring 2018. Since commercial CAR-T cell treatment (tisagenlecleucel) was approved by the FDA/EMA in 2018, eligible patients were preferentially offered CAR-T cell treatment over enrolment in this experimental study, and this has led to a significant drop in expected trial recruitment. After two years of experience with tisagenlecleucel, relapse after cellular immunotherapy is emerging as a new therapeutic challenge (29). Relapses after CAR-T cell failure, especially CD19 negative relapses, and relapsed/refractory T cell ALL should be considered/screened for the SeluDex trial. For patients who have not had CD19 or CD22 targeting immunotherapy these should be prioritised, however, in exceptional circumstances the SeluDex trial (e.g., cycle 1) may also be considered as bridging therapy before CAR-T cell infusion.

Implemented urgent safety measures

Since the study opened to recruitment in 2018 there have been three urgent safety measures (USM) implemented in the protocol to date. The first USM was in December 2018 to reduce the dose of dexamethasone after two weeks during cycle 1 for Group A patients, after an adult patient experienced a fatal Suspected Unexpected Adverse Reaction (SUSAR) from rapid onset gram-negative sepsis; investigators suspected that the steroid administration had masked potential symptoms of infection and sepsis. The second USM was implemented in May 2019 to reduce the dose of dexamethasone after two weeks during cycle 1 for Group P patients, in line with the measure already implemented for adults, in addition to mandating fluoroquinolone prophylaxis during cycle 1 for all patients. This was due to a further

For peer review only

fatal SUSAR of gram-negative sepsis in a paediatric patient. The third USM implemented in September 2019 changed the dexamethasone dose to pulsed during cycle 1 and mandated fluoroquinolone prophylaxis in cycle 2, in addition to cycle 1, and co-trimoxazole prophylaxis throughout trial treatment for all patients. This was due to a further fatal SUSAR in an adult patient from pneumonia considered related to trial treatment. Pulsed dosing of dexamethasone (four days on, three days off) is standard practice in the management of adult ALL patients and is better tolerated than continuous dexamethasone dosing during induction. Since these USMs were implemented, there have been no further events of concern and the TMG, TSC and regulatory authorities are confident that the potential patient benefit outweighs the risks of the intervention.

Trial strengths

This trial is, to the best of our knowledge, one of the first to span the whole age range of paediatric and adult patients. On the paediatric side, it is only limited because participants need to swallow the selumetinib capsules whole. As soon as a paediatric liquid formulation is available, all age limitations could be removed.

The use of the CRM greatly also aided in the delivery of this trial, providing the flexibility to utilise cohorts of only two patients allowing for more frequent dose adjustment. Additionally, the CRM provides the means to estimate probabilities of DLT at all doses along with associated uncertainty intervals for those estimates, even those untested doses. The CRM in general identifies the MTD with greater accuracy and treats more patients at the MTD than traditional rule-based methods e.g. 3+3 (30).

Finally, the paediatric part of the SeluDex trial is an approved Innovative Therapies for Children with Cancer in Europe (ITCC) study with five National Co-ordinating Centres (NCC) outside the UK. The NCC of the Netherlands opened in Summer 2020, with Germany, France, Italy and Denmark planned to follow in 2021. This will help to improve the slow recruitment numbers resulting from the introduction of CAR-T cell therapy.

Ethics and dissemination

The trial will be performed in accordance with the recommendations guiding physicians in biomedical research involving human subjects, adopted by the 18th World Medical Association General Assembly, Helsinki, Finland and stated in the respective participating countries laws governing human research, and Good Clinical Practice. The protocol was approved by the medical ethical committees of all the participating countries. UK ethics approval (17/YH/0123) was granted by Yorkshire & The Humber - Leeds West Research Ethics Committee on 12th July 2017. Initial UK Competent Authority approval was granted by Medicines and Healthcare Products Regulatory Agency (MHRA) on 5th May 2017, with subsequent protocol versions approved; current version in use is 9.0, 22nd March 2021.

A meeting will be held after the end of the study to allow discussion of the main results among the collaborators before publication. Results of the primary and secondary endpoints will be submitted for publication in peer-reviewed journals. Manuscripts will be prepared by the TMG and authorship determined by mutual agreement.

Summary

In summary, the SeluDex trial investigates the novel combination of the MEK1/2 inhibitor, selumetinib, with dexamethasone in a seamless phase I/II Bayesian trial design, in both adult and paediatric

For peer review only

relapsed/refractory ALL patients. Although CAR T-cell therapy was approved after the trial started recruitment, the trial may serve as a bridging treatment option for patients awaiting CAR T-cell therapy and a treatment option for relapses failure once CAR-T cell treatment has failed.

Contributors

TM: Chief Investigator, study conception, study design, protocol development. DS: Senior Trial Biostatistician, study design, statistical plan development. JS: Trial Team Leader, study management, protocol development, coordination of IRAS, REC, MHRA, HRA and local R&D applications. SJ: Trial Coordinator, protocol development, coordination of IRAS, REC, MHRA, HRA and local R&D applications. PRK: Co-Investigator, study conception, study design, protocol development. JV: International Lead Investigator, study conception, study design, protocol development. BV: Co-Investigator, study conception, study design, protocol development. JI: Biological Coordinator, study conception, study design, protocol development. GV: Biological Coordinator, study conception, study design, protocol development. GS: Co-Investigator, protocol development. RP: Co-Investigator, study conception, study design, protocol development. LB: Chief Biostatistician, study conception, study design, protocol development, statistical plan development.

Funding

This work is supported by Cancer Research UK (C27943/A22304), CRUK trial number CRUKD/16/015 and (C27943/A23260), CRUK trial number CRUKD/16/016, and AstraZeneca through the CRUK's Combinations Alliance and Experimental Cancer Medicine Centre (ECMC). AstraZeneca provides Selumetinib to participating sites free-of-charge, and was consulted over the trial design but are not involved in the trial management group or safety committee. National Coordinating Centres were supported by a grant from ITCC "Imagine for Margo" fund and AstraZeneca. Staff at the CRCTU are also supported by a core funding grant from Cancer Research UK (C22436/A25354).

Role of funders and sponsor

Neither the sponsor or funders had any role in trial design, data collection, data analysis, data interpretation or writing of the report. The corresponding author had full access to all the data in the trial and had final responsibility for the decision to submit for publication.

Competing interests

JI has received research funding from F. Hoffmann-La Roche Ltd. All other authors declare no other relevant conflicts of interest.

Acknowledgements

The Investigators and Sponsor thank all the patients and their families who participated in this trial, as well as the NHS Trusts and staff and the members of the Trial Safety Committee, chaired by Dr Bela Wrench, who have supported this trial. SeluDex was supported by Experimental Cancer Medicine Centres (ECMC) funding and by the ECMC Network. This trial has been independently peer reviewed and has been adopted by the National Institute for Health Research Clinical Research Network Portfolio. Special thanks to Dr Siân Lax for her contributions to this paper.

For peer review only

References

1. Kaatsch P. Epidemiology of childhood cancer. Cancer Treat Rev. 2010;36(4):277-85.

2. Einsiedel HG, von Stackelberg A, Hartmann R, Fengler R, Schrappe M, Janka-Schaub G, et al. Long-term outcome in children with relapsed ALL by risk-stratified salvage therapy: results of trial acute lymphoblastic leukemia-relapse study of the Berlin-Frankfurt-Münster Group 87. J Clin Oncol. 2005;23(31):7942-50.

3. Bassan R, Hoelzer D. Modern Therapy of Acute Lymphoblastic Leukemia. Journal of Clinical Oncology. 2011;29(5):532-43.

4. Geyer MB, Hsu M, Devlin SM, Tallman MS, Douer D, Park JH. Overall survival among older US adults with ALL remains low despite modest improvement since 1980: SEER analysis. Blood. 2017;129(13):1878-81.

5. Irving J, Matheson E, Minto L, Blair H, Case M, Halsey C, et al. Ras pathway mutations are prevalent in relapsed childhood acute lymphoblastic leukemia and confer sensitivity to MEK inhibition. Blood. 2014;124(23):3420-30.

6. Liu Y, Easton J, Shao Y, Maciaszek J, Wang Z, Wilkinson MR, et al. The genomic landscape of pediatric and young adult T-lineage acute lymphoblastic leukemia. Nature Genetics. 2017;49(8):1211-8.

7. Ryan SL, Matheson E, Grossmann V, Sinclair P, Bashton M, Schwab C, et al. The role of the RAS pathway in iAMP21-ALL. Leukemia. 2016;30(9):1824-31.

8. Zhang J, Ding L, Holmfeldt L, Wu G, Heatley SL, Payne-Turner D, et al. The genetic basis of early T-cell precursor acute lymphoblastic leukaemia. Nature. 2012;481(7380):157-63.

9. Zhang J, Mullighan CG, Harvey RC, Wu G, Chen X, Edmonson M, et al. Key pathways are frequently mutated in high-risk childhood acute lymphoblastic leukemia: a report from the Children's Oncology Group. Blood. 2011;118(11):3080-7.

10. Roberts KG, Li Y, Payne-Turner D, Harvey RC, Yang YL, Pei D, et al. Targetable kinase-activating lesions in Ph-like acute lymphoblastic leukemia. New England Journal of Medicine. 2014;371(11):1005-15.

11. Holmfeldt L, Wei L, Diaz-Flores E, Walsh M, Zhang J, Ding L, et al. The genomic landscape of hypodiploid acute lymphoblastic leukemia. Nat Genet. 2013;45(3):242-52.

12. Shochat C, Tal N, Bandapalli OR, Palmi C, Ganmore I, te Kronnie G, et al. Gain-of-function mutations in interleukin-7 receptor-α (IL7R) in childhood acute lymphoblastic leukemias. J Exp Med. 2011;208(5):901-8.

13. Zenatti PP, Ribeiro D, Li W, Zuurbier L, Silva MC, Paganin M, et al. Oncogenic IL7R gain-of-function mutations in childhood T-cell acute lymphoblastic leukemia. Nat Genet. 2011;43(10):932-9.

14. Jeong EG, Kim MS, Nam HK, Min CK, Lee S, Chung YJ, et al. Somatic mutations of JAK1 and JAK3 in acute leukemias and solid cancers. Clin Cancer Res. 2008;14(12):3716-21.

15. Goldinger SM, Zimmer L, Schulz C, Ugurel S, Hoeller C, Kaehler KC, et al. Upstream mitogen-activated protein kinase (MAPK) pathway inhibition: MEK inhibitor followed by a BRAF inhibitor in advanced melanoma patients. Eur J Cancer. 2014;50(2):406-10.

16. Carvajal RD, Piperno-Neumann S, Kapiteijn E, Chapman PB, Frank S, Joshua AM, et al. Selumetinib in Combination With Dacarbazine in Patients With Metastatic Uveal Melanoma: A Phase III, Multicenter, Randomized Trial (SUMIT). J Clin Oncol. 2018;36(12):1232-9.

17. Chung V, McDonough S, Philip PA, Cardin D, Wang-Gillam A, Hui L, et al. Effect of Selumetinib and MK-2206 vs Oxaliplatin and Fluorouracil in Patients With Metastatic Pancreatic Cancer After Prior Therapy: SWOG S1115 Study Randomized Clinical Trial. JAMA Oncol. 2017;3(4):516-22.

18. Krishnamurthy A, Dasari A, Noonan AM, Mehnert JM, Lockhart AC, Leong S, et al. Phase Ib Results of the Rational Combination of Selumetinib and Cyclosporin A in Advanced Solid Tumors with an Expansion Cohort in Metastatic Colorectal Cancer. Cancer Res. 2018;78(18):5398-407.

19. Jänne PA, Shaw AT, Pereira JR, Jeannin G, Vansteenkiste J, Barrios C, et al. Selumetinib plus docetaxel for KRAS-mutant advanced non-small-cell lung cancer: a randomised, multicentre, placebo-controlled, phase 2 study. Lancet Oncol. 2013;14(1):38-47.

For peer review only

20. Matheson EC, Thomas H, Case M, Blair H, Jackson RK, Masic D, et al. Glucocorticoids and selumetinib are highly synergistic in RAS pathway-mutated childhood acute lymphoblastic leukemia through upregulation of BIM. Haematologica. 2019;104(9):1804-11.

21. McCune JS, Hawke RL, LeCluyse EL, Gillenwater HH, Hamilton G, Ritchie J, et al. In vivo and in vitro induction of human cytochrome P4503A4 by dexamethasone. Clin Pharmacol Ther. 2000;68(4):356-66.

22. Banerjee A, Jakacki RI, Onar-Thomas A, Wu S, Nicolaides T, Young Poussaint T, et al. A phase I trial of the MEK inhibitor selumetinib (AZD6244) in pediatric patients with recurrent or refractory low-grade glioma: a Pediatric Brain Tumor Consortium (PBTC) study. Neuro Oncol. 2017;19(8):1135-44.

23. Dombi E, Baldwin A, Marcus LJ, Fisher MJ, Weiss B, Kim A, et al. Activity of Selumetinib in Neurofibromatosis Type 1-Related Plexiform Neurofibromas. N Engl J Med. 2016;375(26):2550-60.

24. O'Quigley J, Pepe M, Fisher L. Continual reassessment method: a practical design for phase 1 clinical trials in cancer. Biometrics. 1990;46(1):33-48.

25. Program CTE. Common Terminology Criteria for Adverse Events (CTCAE), Version 4.03. US Department of Health and Human Services, NIH, NCI. 2010.

26. O'Connor L, Strasser A, O'Reilly LA, Hausmann G, Adams JM, Cory S, et al. Bim: a novel member of the Bcl-2 family that promotes apoptosis. Embo j. 1998;17(2):384-95.

27. Goodman SN, Zahurak ML, Piantadosi S. Some practical improvements in the continual reassessment method for phase I studies. Stat Med. 1995;14(11):1149-61.

28. Ko RH, Ji L, Barnette P, Bostrom B, Hutchinson R, Raetz E, et al. Outcome of patients treated for relapsed or refractory acute lymphoblastic leukemia: a Therapeutic Advances in Childhood Leukemia Consortium study. J Clin Oncol. 2010;28(4):648-54.

29. Nie Y, Lu W, Chen D, Tu H, Guo Z, Zhou X, et al. Mechanisms underlying CD19-positive ALL relapse after anti-CD19 CAR T cell therapy and associated strategies. Biomarker Research. 2020;8(1):18.

30. Wheeler GM, Mander AP, Bedding A, Brock K, Cornelius V, Grieve AP, et al. How to design a dose-finding study using the continual reassessment method. BMC Medical Research Methodology. 2019;19(1):18.

For peer review only

Figure Legend

Figure 1: SeluDex trial schema

SeluDex trial schema showing the patient pathway highlighting screening, trial entry, treatment, and follow-up.

For peer review only

Table 1: Main eligibility criteria for Group A in the SeluDex trial

Main Inclusion Criteria for Group A

Morphologically proven relapsed/refractory (M2 or M3 marrow; ≥1st relapse) or progressive B cell precursor or T-Acute Lymphoblastic Leukaemia (ALL) with demonstrated RAS pathway activating mutations (NRAS, KRAS, FLT3, PTPN11, cCBL, NF1, BRAF, IKZF2, IKZF3, IL7Rα or JAK1) identified during the trial screening process

B cell precursor patients must either:

- Have received CAR -T cell therapy, or
- Be awaiting CAR -T cell therapy, or
- Be considered ineligible for CAR -T cell therapy

≥18 years of age

Adequate renal function: Serum creatinine <1.5 x upper limit of normal (ULN)

Patient is able to swallow selumetinib capsules whole

Eastern Cooperative Oncology Group (ECOG) ≤2

Women of childbearing potential must have a negative pregnancy test

Patients who are women of childbearing potential and male patients with partners who are women of childbearing potential must agree to use appropriate contraception (see section 7.9.3 for definition) whilst on trial.

Written informed consent

Patients must have a body surface area (BSA) ≥ 0.55 m^2

Main Exclusion Criteria for Group A

ALL without presence of RAS-pathway activating mutations

Mature B-cell leukaemia and Philadelphia positive ALL

Prior exposure to MEK, RAS or RAF inhibitors

Any unresolved toxicity ≥ CTCAE Grade 2 from previous anti-cancer therapy, except for alopecia

Cardiac conditions as follows:

- Prior or current cardiomyopathy including but not limited to the following:
  - Known hypertrophic cardiomyopathy
  - Known arrhythmogenic right ventricular cardiomyopathy
- Even if full recovery has occurred, previous moderate or severe impairment of left ventricular systolic function (LVEF <45% on ECHO)
- Severe valvular heart disease
- Severe congenital heart disease
- Uncontrolled hypertension: BP ≥150/95 mmHg despite medical therapy
- Baseline (LVEF) below the lower limit of normal (LLN) or <55% measured by ECHO
- Acute coronary syndrome within 6 months prior to trial registration
- Uncontrolled Angina - Canadian Cardiovascular Society grade II-IV despite medical therapy
- Symptomatic heart failure New York Heart Association (NYHA) Class II-IV, prior or current cardiomyopathy, or severe valvular heart disease
- Atrial fibrillation with a ventricular rate >100 bpm on Electrocardiogram (ECG) at rest
- QTcF >450ms in male patients or ≥460ms in female patients, or other factors that increase the risk of QT prolongation

Ophthalmological conditions as follows:

- Current or past history of retinal pigment epithelial detachment (RPED)/central serous retinopathy (CSR) or retinal vein occlusion (RVO)
- Intraocular pressure (IOP) > 21 mmHg or uncontrolled glaucoma (irrespective of IOP)

Pregnant and breast-feeding females

For peer review only

Known severe hypersensitivity to selumetinib, dexamethasone or combination medications or any excipient of these medicinal products, or history of allergic reactions attributed to compounds of similar chemical or biologic composition to selumetinib

For peer review only

Table 2: Main eligibility criteria for Group P in the SeluDex trial

Main Inclusion Criteria for Group P

Morphologically proven relapsed/refractory (M2 or M3 marrow; ≥2nd relapse) or progressive B cell precursor or T-Acute Lymphoblastic Leukaemia (ALL) with demonstrated RAS pathway activating mutations (NRAS, KRAS, FLT3, PTPN11, cCBL, NF1, BRAF, IKZF2, IKZF3, IL7Rα or JAK1) identified during the trial screening process

B cell precursor patients must either:

- Have received CAR -T cell therapy, or
- Be awaiting CAR -T cell therapy, or

Be considered ineligible for CAR -T cell therapy

<18 years of age

Adequate renal function:

- ≤ 5 years: Serum creatinine <0.8 mg/dL or 70 µmol/L
- > 5 years but ≤ 10 years: Serum creatinine <1 mg/dL or 88 µmol/L
- > 10 years but ≤ 15 years: Serum creatinine <1.2 mg/dL or 106 µmol/L
- > 15 years: Serum creatinine <1.5 mg/dL or 132 µmol/L

Patient is able to swallow selumetinib capsules whole

Lansky play scale ≥60% or Karnofsky scale ≥60%

Women of childbearing potential must have a negative pregnancy test

Patients who are women of childbearing potential and male patients with partners who are women of childbearing potential must agree to use appropriate contraception (see section 7.9.3 for definition) whilst on trial.

Written informed consent

Patients must have a body surface area (BSA) ≥ 0.55 m^2

Main Exclusion Criteria for Group P

ALL without presence of RAS-pathway activating mutations

Mature B-cell leukaemia and Philadelphia positive ALL

Prior exposure to MEK, RAS or RAF inhibitors

Any unresolved toxicity ≥ CTCAE Grade 2 from previous anti-cancer therapy, except for alopecia

Cardiac conditions as follows:

- Prior or current cardiomyopathy including but not limited to the following:
  - Known hypertrophic cardiomyopathy
  - Known arrhythmogenic right ventricular cardiomyopathy
- Even if full recovery has occurred, previous moderate or severe impairment of left ventricular systolic function (SF <29% but excluding transient impairments due to e.g., anaemia/sepsis or results not thought to represent a true reflection of cardiac function)
- Severe valvular heart disease
- Severe congenital heart disease
- Uncontrolled hypertension: BP ≥95th percentile for age, height and gender
- Baseline SF <29%
- Atrial fibrillation with a ventricular rate >130 bpm on Electrocardiogram (ECG) at rest
- QTcF >450ms in patients <12 years or ≥460ms in patients ≥12 but <18 years

Ophthalmological conditions as follows:

- Current or past history of retinal pigment epithelial detachment (RPED)/central serous retinopathy (CSR) or retinal vein occlusion
- Intraocular pressure (IOP) > 21 mmHg or uncontrolled glaucoma (irrespective of IOP)

Pregnant and breast-feeding females

For peer review only - http://bmjopen.bmj.com/site/about/guidelines.xhtml

Known severe hypersensitivity to selumetinib, dexamethasone or combination medications or any excipient of these medicinal products, or history of allergic reactions attributed to compounds of similar chemical or biologic composition to selumetinib

For peer review only

Table 3: Group A (≥18 years) dosing schedule in phase I dose finding

Dose Level	Selumetinib (% of adult MTD)<br>Cycle 1 D1: single dose<br>Cycle 1 D4-D28: BD dosing<br>Subsequent cycles 2-6: D1-28: BD dosing		Dexamethasone<br>Cycle 1 D2-4 & D8-11: 6mg/m²/day, D15-18 & D22-25: 4mg/m²/day<br>Cycle 2 D1-4: 4mg/m²/day<br>Subsequent cycles 3-6, D1-5 only 6mg/m²/day	Initial Design<br>Applicable prior to DLTs
-1	80%	60mg BD PO	mg/m²***/day PO divided into 2 doses (as per local practice)	n/a
0	100%	75mg BD PO		Cohorts 1 and 2
+1*	113-120%**	85-90mg** BD PO		Cohorts 3, 4, 5 and 6

* **Important:** The selumetinib dose reverts from the +1 dose level to dose level 0 with the start of cycle 2 as patients will only be exposed to a short pulse of dexamethasone which is not expected to substantially affect selumetinib PK

** The proposed increase of dose might be changed based on PK results of dose level 0

*** Body surface area (BSA) for Group A to be calculated according to standard institutional practice

For peer review only

Table 4: Group P (<18 years) dosing schedule in phase I dose finding

**Dose Level**	**Selumetinib (% of adult MTD)**<br>Cycle 1 D1: single dose<br>Cycle 1 D4-D28: BD dosing<br>Subsequent cycles 2-6: D1-28: BD dosing		**Dexamethasone**<br>Cycle 1 D2-4 & D8-11: 6mg/m²/day, D15-18 & D22-25: 4mg/m²/day<br>Cycle 2 D1-4: 4mg/m²/day<br>Subsequent cycles 3-6, D1-5 only 6mg/m²/day	**Initial Design**<br>Applicable prior to DLTs
-1	80%	20mg/m²*** BD PO	mg/m²***/day PO divided into 2 doses (as per local practice)	Cohorts 1 and 2
0	100%	25mg/m²*** BD PO		Cohorts 3 and 4
+1*	113-120%**	28-30mg**/m²*** BD PO		Cohorts 5 and 6

* **Important:** The selumetinib dose reverts from the +1 dose level to dose level 0 with the start of cycle 2 as patients will only be exposed to a short pulse of dexamethasone which is not expected to substantially affect selumetinib PK

** The proposed increase of dose might be changed based on PK results of dose level 0

*** BSA for Group P to be calculated according to standard institutional practice

For peer review only

Table 5: Restricted medications within the SeluDex trial

• Acetazolamide<br>• Aminoglutethimide<br>• Antacids<br>• Anticholinesterases<br>• Anti-hypertensives<br>• Aprepitant<br>• Barbiturates<br>• Beta-naphthoflavone<br>• Carbamazepine<br>• Carbenoxolone<br>• Clarithromycin<br>• Coumarin anticoagulants<br>• Diltiazem<br>• Diuretics<br>• Efavirenz<br>• Ephedrine<br>• Erythromycin	• Fluconazole<br>• Fluvoxamine<br>• Glucocorticoids<br>• Hypoglycaemic agents, e.g. insulin<br>• Indinavir<br>• Itraconazole<br>• Ketoconazole<br>• Loop diuretics<br>• Methylcholanthrene<br>• Modafinil<br>• Nafcillin<br>• Nefazodone<br>• Nelfinavir<br>• Nevirapine<br>• Norethindrone<br>• NSAIDs<br>• Omeprazole	• Oral contraceptives<br>• Oxcarbazepine<br>• Phenobarbital<br>• Phenytoin<br>• Pioglitazone<br>• Prednisolone<br>• Primidone<br>• Rifabutin<br>• Rifampicin<br>• Ritonavir<br>• Salicylates<br>• Saquinavir<br>• Suboxone<br>• Telithromycin<br>• Thiazide diuretics<br>• Troglitazone<br>• Verapamil<br>• St John's Wort

For peer review only

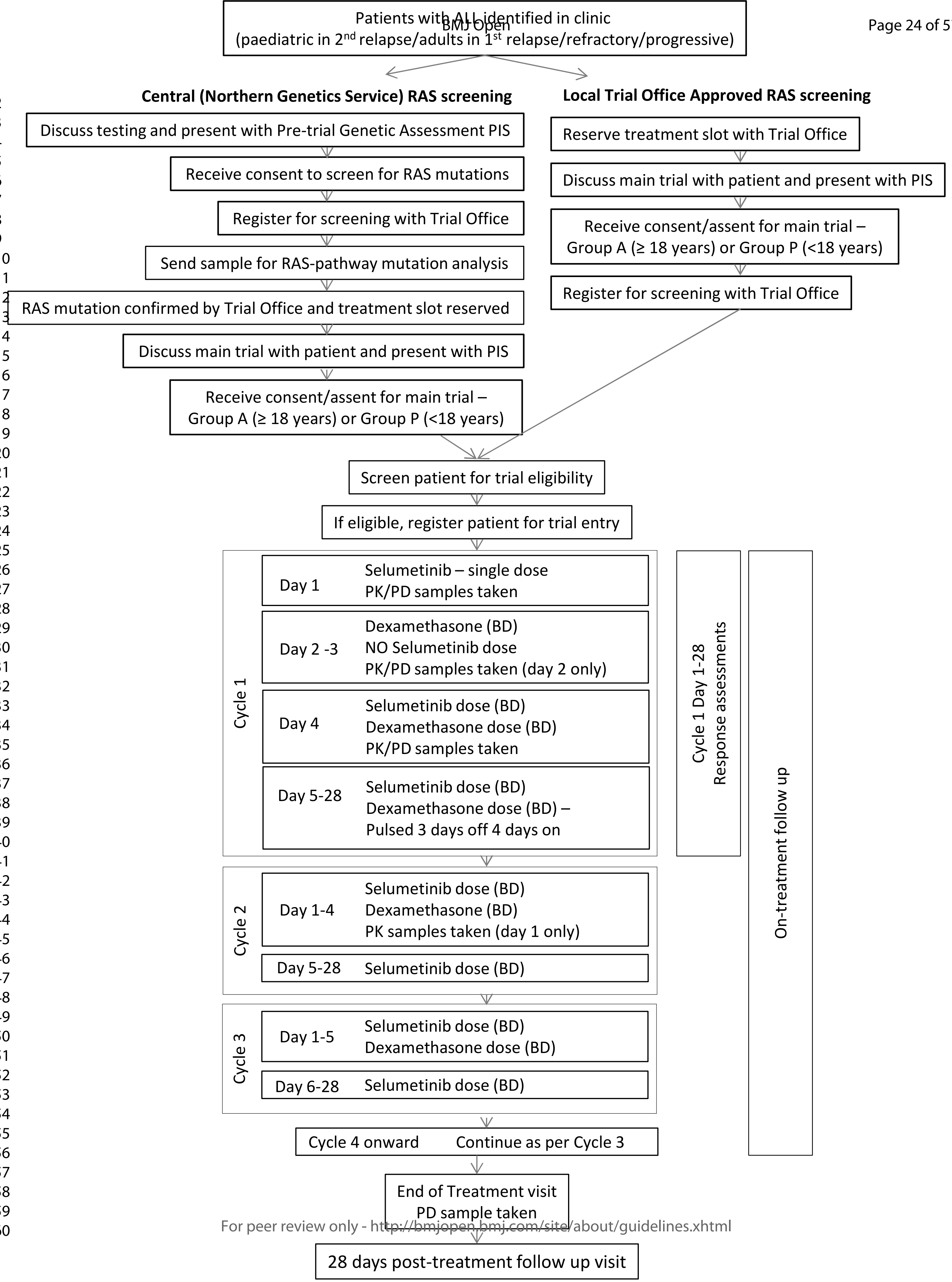
Patients with ALL identified in clinic
(paediatric in 2nd relapse/adults in 1st relapse/refractory/progressive)
Central (Northern Genetics Service) RAS screening
Discuss testing and present with Pre-trial Genetic Assessment PIS
Receive consent to screen for RAS mutations
Register for screening with Trial Office
Send sample for RAS-pathway mutation analysis
RAS mutation confirmed by Trial Office and treatment slot reserved
Discuss main trial with patient and present with PIS
Receive consent/assent for main trial – Group A (≥ 18 years) or Group P (<18 years)
Local Trial Office Approved RAS screening
Reserve treatment slot with Trial Office
Discuss main trial with patient and present with PIS
Receive consent/assent for main trial – Group A (≥ 18 years) or Group P (<18 years)
Register for screening with Trial Office
Screen patient for trial eligibility
If eligible, register patient for trial entry
Cycle 1
Day 1
Selumetinib – single dose
PK/PD samples taken
Day 2 -3
Dexamethasone (BD)
NO Selumetinib dose
PK/PD samples taken (day 2 only)
Day 4
Selumetinib dose (BD)
Dexamethasone dose (BD)
PK/PD samples taken
Day 5-28
Selumetinib dose (BD)
Dexamethasone dose (BD) –
Pulsed 3 days off 4 days on
Cycle 1 Day 1-28
Response assessments
On-treatment follow up
Cycle 2
Day 1-4
Selumetinib dose (BD)
Dexamethasone (BD)
PK samples taken (day 1 only)
Day 5-28
Selumetinib dose (BD)
Cycle 3
Day 1-5
Selumetinib dose (BD)
Dexamethasone dose (BD)
Day 6-28
Selumetinib dose (BD)
Cycle 4 onward
Continue as per Cycle 3
End of Treatment visit
PD sample taken
28 days post-treatment follow up visit

Supplementary Appendix 1

Data category	Information
Primary registry and trial identifying number	ISRCTN: 92323261
Date of registration in primary registry	Date applied: 22-Jan-2018<br>Date assigned: 23-May-2018. Due to administrative delays, the date assigned occurred five days after the first patient was recruited.
Secondary identifying numbers	EudraCT: 2016-003904-29ClinicalTrials.Gov: NCT03705507<br>ITCC-063 study
Source(s) of monetary or material support	Cancer Research UK<br>AstraZeneca<br>National Coordinating Centres were supported by a grant from ITCC "Imagine for Margo" fund and AstraZeneca
Primary sponsor	University of Birmingham
Secondary sponsor(s)	n/a
Contact for public queries	LB: L.J.Billingham@bham.ac.uk
Contact for scientific queries	TM: tobiasmenne@nhs.net
Public title	A trial looking at selumetinib and dexamethasone for acute lymphoblastic leukaemia (SeluDex)
Scientific title	SeluDex: an international trial of selumetinib in combination with dexamethasone for the treatment of acute lymphoblastic leukaemia

For peer review only

SeluDex

**Data category**	**Information**
Countries of recruitment	UK, Denmark, Italy, Germany, France, Netherlands
Health condition(s) or problem(s) studied	Relapsed/refractory acute lymphoblastic leukaemia
Intervention(s)	Dexamethasone and selumetinib
Key inclusion and exclusion criteria: **Adult Group**	Ages eligible for study: ≥18 years<br>Sexes eligible for study: both<br>Accepts healthy volunteers: no
	Inclusion criteria: adult patient (≥18 years) with proven ALL with demonstrated RAS pathway activating mutations, performance status ≤2
	Exclusion criteria: Prior exposure to MEK, RAS or RAF inhibitors, pregnancy or breastfeeding females, cardiac and/or ophthalmology conditions
Key inclusion and exclusion criteria: **Paediatric Group**	Ages eligible for study: <18 years<br>Sexes eligible for study: both<br>Accepts healthy volunteers: no
	Inclusion criteria: paediatric patient (<18 years) with proven ALL with demonstrated RAS pathway activating mutations, able to swallow selumetinib capsules whole, Lansky play scale ≥60% or Karnofsky scale ≥60%
	Exclusion criteria: Prior exposure to MEK, RAS or RAF inhibitors, pregnancy or breastfeeding females, cardiac and/or ophthalmology conditions
Study type	Interventional
	Allocation: non-randomised, open-label

For peer review only

SeluDex

Data category	Information
	Primary purpose: dose-finding and preliminary efficacy
	Phase I/II
Date of first enrolment	18-May-2018
Target sample size	Between 26 and 42 patients; minimum of 13 and maximum of 21 in each group,
Recruitment status	Open
Primary outcome(s)	Phase I: Occurrence/non-occurrence of dose limiting toxicities (time frame: between days 1 and 28 of cycle 1)<br>Phase II: Morphological response (time from: 28 days post-treatment end)
Key secondary outcome(s)	Occurrence of AEs as measured by CTCAE version 4 and causality assessment<br>Pharmacokinetic variables of selumetinib in combination with dexamethasone from the concentration time profile

For peer review only

__Please delete this line and print on hospital headed paper__

SeluDex

SeluDex: An international phase I/II expansion trial of the MEK inhibitor selumetinib in combination with dexamethasone for the treatment of relapsed/refractory RAS-pathway mutated paediatric and adult Acute Lymphoblastic Leukaemia

Informed Consent Form – Adult, Phase I

To be used only for participants over 16 years of age entering dose finding phase

EudraCT Reference: 2016-003904-29

Site: ______________________________

Principal Investigator: ______________________________

Trial Number (TNO): ☐☐☐ Screening Number (SNO): ☐☐ / ☐☐

Please INITIAL each box

1. I confirm that I have read and understand the ***Participant Information Sheet – Adult, Phase I*** (version……... dated…………………………) for the above trial. I have had the opportunity to consider the information, ask questions, and have had these answered satisfactorily ☐

2. I understand that my participation is voluntary and that I am free to withdraw at any time without giving any reason, without my medical care or legal rights being affected. I understand that if I withdraw from treatment my doctor may continue to provide the Trial Office with information that would routinely be collected about me and recorded in my medical notes. I am aware that I can also withdraw consent for this data transfer ☐

3. I give permission for my initials, date of birth, hospital number and NHS number to be given to the SeluDex Trial Office when I am registered to the trial as well as a copy of this consent form ☐

4. I understand that relevant sections of my medical notes and data collected during the trial may be looked at by individuals from the SeluDex Trial Office, regulatory authorities, Sponsor and/or NHS bodies, where it is relevant to my taking part in this research. I understand that this information will be held in a confidential manner. I give permission for ☐

Original to be kept in the Investigator Site File, 1 copy in hospital notes, 1 copy to the participant, 1 copy to the National Coordinating Centre

For peer review only - http://bmjopen.bmj.com/site/about/guidelines.xhtml

these individuals to have access to my records.

5. I understand that anonymised data from the trial may be provided to other third parties (e.g. pharmaceutical companies or other academic institutions) for research, safety monitoring or licensing purposes ☐

6. I agree to my GP being informed of my participation in this trial ☐

7. I understand that the SeluDex Trial Office, may access information held by national cancer registries and within national databases to keep in touch with me and to follow up on my health status ☐

8. I give permission for collection of samples of my blood and bone marrow to be used in the SeluDex trial. I understand that samples will be sent to the University of Birmingham and other laboratories in the United Kingdom ☐

9. I understand that DNA analysis may be performed on the samples taken for the trial. ☐

10. I agree to take part in the above trial

The following is <u>optional</u> and will not affect entry into the trial, please initial in the relevant box:

	**No**	**Yes**
If in the event that an abnormality that might affect other family members is uncovered during genetic testing for the trial, I would like my doctor to be informed and to be referred to a genetic counsellor if appropriate.	☐	☐

**Name of participant**	**Date**	**Signature**

**Name of person taking consent**	**Date**	**Signature**

You must have signed the Site Signature & Delegation Log

This document was drafted using CRCTU-ICF-QCD-001, version 2.0

Original to be kept in the Investigator Site File, 1 copy in hospital notes, 1 copy to the participant, 1 copy to the National Coordinating Centre

For peer review only - http://bmjopen.bmj.com/site/about/guidelines.xhtml

<u>Please delete this line and print on hospital headed paper</u>

SeluDex

SeluDex: An international trial of selumetinib in combination with dexamethasone for the treatment of Acute Lymphoblastic Leukaemia.

Participant Information Sheet – Adult, Phase I

To be used only for patients over 16 years of age considering entering dose finding phase

---

This leaflet provides information about a clinical research trial and is intended to supplement your discussions about the trial with your doctor and nurses. Having read it, you may have further questions and these should be discussed with your consultant or one of the research team.

Dear Patient,

We would like to invite you to take part in a non-commercial, clinical trial being run by the University of Birmingham. Before you decide whether to take part in this trial, it is important for you to understand why the research is being done and what it would involve for you.

Please take time to read the following information carefully, and discuss it with your friends and relatives if you wish. Your doctor will go through the information sheet with you, and answer any questions you may have.

- Part 1 tells you the purpose of this trial, and what will happen to you if you take part
- Part 2 gives you more detailed information about the conduct of the trial

If there is anything that is not clear, or if you would like more information, please ask your doctor.

Take your time to decide whether or not you wish to take part. If you decide not to take part, this will not affect the quality of your care.

For peer review only - http://bmjopen.bmj.com/site/about/guidelines.xhtml

SeluDex

PART 1

What is the purpose of the trial?

The purpose of this trial is to test a new drug called selumetinib in combination with another drug called dexamethasone. The trial specifically includes those patients who have a relapse of their acute lymphoblastic leukaemia (ALL) or refractory ALL, and who have an identified change (mutation) in a particular gene in their cancer's DNA. We would like to see what effect combining these two drugs has on you and your leukaemia. This will include looking at how well this treatment works, finding out more information about how it affects the disease, and to see how safe the drugs are.

During Phase I, the part you have been invited to participate in, the trial will look at establishing what is the most suitable dose level of selumetinib in combination with dexamethasone. The purpose of this step of the research is to see what dose of selumetinib we can safely give to participants. We will get some preliminary information regarding the effectiveness of this combined treatment. We will also use results from your tests to see how the treatment affects the body.

After the Phase I part of the trial is complete, further patients who have not taken part in Phase I will be invited to participate in Phase II. The Phase II part of the trial will look at a dose level of selumetinib which has already been established in Phase I as being the most effective in combination with dexamethasone. The purpose of this step of the research is to develop further evidence about this recommended dose, and to see what effects the combination of these medications will have on participants' leukaemia. We will also continue to use results from tests to see how the treatment affects the body.

Why have I been invited to participate?

You have been invited to take part in the Phase I part of the trial because you have been diagnosed with relapsed ALL or with refractory ALL. If you have relapsed ALL and you are over 18 years of age this will be at least your first relapse whereas if you are 16-18 years of age this will be at least your second relapse. You will have previously consented to us undertaking a genetic test, or already had this testing done at hospital as part of your normal care, to assess the genetic status of your disease. This test has shown that your leukaemia carries a mutation in the RAS pathway, which means that you are a suitable candidate to take part in the SeluDex trial. Up to 42 participants will be recruited to the trial in total.

Do I have to take part?

No, you do not have to take part. It is up to you to decide whether or not to join the trial. Your doctor will describe the trial, and will go through this information sheet with you. They will also give you a copy of the information sheet to keep. If you decide to be part of the trial, you will be asked to sign a consent form to show you have agreed to participate. You are free to withdraw at any time, without giving a reason, and this will not affect the standard of care you receive thereafter.

What are the drugs being tested?

The investigational drugs being tested in this trial are called 'selumetinib' and 'dexamethasone'.

Selumetinib is manufactured and supplied by AstraZeneca, and it has not yet been licensed by the Medicines and Healthcare products Regulatory Agency (MHRA) and therefore only available as part of a clinical trial. As researchers learn more about the gene changes in cells

For peer review only - http://bmjopen.bmj.com/site/about/guidelines.xhtml

that cause cancer, they have been able to develop drugs that target these changes. Selumetinib is an example of such a drug. Cells normally grow in an orderly way. Chemical messages or signals tell them when to grow and when to stop. But if a gene becomes abnormal, a protein called MEK can produce a signal which makes cancer cells grow abnormally. Selumetinib is a MEK inhibitor, which means that it stops MEK proteins from sending signals to cancer cells for them to grow. This may stop or slow down the growth of cancer cells. As of 31 January 2020, approximately 4,335 patients have received selumetinib on its own, or in combination with another drug. You will take one dose of selumetinib on the first day of your first cycle and then from day 4 you will continue taking the drug throughout your treatment.

Dexamethasone is already used for the treatment of a number conditions including cancers and leukaemias, and belongs to a group of drugs known as glucocorticosteroids. These are important in the treatment of leukaemia due their ability to stimulate the death of cancer cells. Dexamethasone will be given as a tablet or oral suspension. You will not start taking dexamethasone until the second day of your first cycle, and will take this drug intermittently throughout treatment. You will take dexamethasone for 3 days during your first week of your first cycle, not take for 3 days and then take for 4 days until the beginning of cycle 2. After this, you will take dexamethasone just for the first five days of each cycle.

The research team at your hospital will provide you with more information on the routine for taking your medicine if you decide to take part in the trial.

If your leukaemia has also been detected in the central nervous system (CNS) (after a test called a lumbar puncture), additional treatment with intrathecal methotrexate (IT MTX) may be administered to help treat the leukaemia in the CNS.

It appears that it is more difficult for ALL participants receiving selumetinib and dexamethasone treatment in the SeluDex trial to ward off infections. Therefore participants have an increased risk of complications from this such as sepsis, which arises when the body's response to infection causes injury to its own tissues and organs requiring quick treatment. This could be due to the way selumetinib works in the body and that dexamethasone can hide symptoms of infection. Because of this you will receive a specific antibiotic (fluoroquinolone) during your first two cycles of treatment and another specific antibiotic (co-trimoxazole) throughout your treatment on the trial to help prevent this. Having preventative antibiotics is often part of your regular cancer care. Your doctor will also discuss with you that if you experience any signs of infection or sepsis (fever, fast heartbeat or breathing) you should contact your treating hospital straight away.

What will happen to me if I take part?

If you decide to take part, you will be asked to sign an Informed Consent Form to show that you have agreed to take part in the trial. After you have signed the consent form, you will undergo a period of screening tests to determine whether you are eligible and that it is safe for you to enter the trial.

Before you begin the trial:

You will need to have the following screening tests to ensure that it is appropriate and safe for you to take part. Some of these procedures are extra to your regular cancer care. If you have had some of them recently, they may not need to be repeated.

Routine tests and procedures

The research team will perform some routine checks. These will include:

- blood, urine tests
- an electrocardiogram (ECG), which will record the electrical activity of the heart
- a review of your medical history

For peer review only - http://bmjopen.bmj.com/site/about/guidelines.xhtml

SeluDex

- a physical examination
- assessment of your vital signs including blood pressure, temperature, heart rate
- review of your performance status, which is a measure of your general health and how your disease affects your daily routine
- you will also be asked questions about your health (e.g. date of diagnosis of your conditions, details of previous treatment or any other illnesses you may have) and what medicines you are taking at the moment
- a pregnancy test if you are female and of child-bearing potential

Extra tests and procedures

In addition to the tests above, an echocardiogram (ECHO) will be performed. This will record how your heart pumps blood and how the valves inside your heart look and work.

You will also have an eye examination. During the eye exam, the ophthalmologist will examine your eyes with a number of different instruments. They will look at the different structures of your eye to check for abnormalities. They may put drops in your eyes which will temporarily affect your vision, so you shouldn't drive yourself home. This test will be performed at screening only, but may be repeated whilst on treatment if deemed necessary by the trial doctor.

Fluid will be taken by lumbar puncture to test for leukaemia in the CNS which may or may not be routine. During this test treatment with intrathecal methotrexate (IT MTX) may be administered to help prevent or treat CNS relapse. This test will be performed for all participants at screening, and repeated at cycle 1 day 28 for those with CNS positive disease.

Please also refer to the summary table at the end of this information sheet. The screening period may last up to 3 weeks. If you are eligible to take part in the trial, you will be registered and given a unique trial number. If your doctor feels it is appropriate for your medical care, you may be given a course of one of three drugs (either dexamethasone, prednisolone or hydroxycarbamide) during the screening period. This treatment will end either seven days prior or up to one day prior to you registering and starting on the trial depending on the choice of pre-treatment.

During the trial:

You will be expected to do the following things during the trial:

- You will have to take selumetinib, twice-daily, continuously throughout the trial, and intermittent dexamethasone during the first and start of the second cycle and then for the first five days of each subsequent cycle
- You will have to have preventative antibiotic treatment
- Whilst taking the trial drugs you will be given a Patient Diary in which to record when you take your medication. Your research nurse will show you how to complete it. The diary will help you to remember if you have taken your dose and to record any side effects. If you accidently miss a dose, it is important that this is recorded in order for your doctor to see what medication you have taken. You will need to bring the diary to your clinic visits, and you will be issued with a new diary at the start of each new cycle of treatment. In addition, you must return the empty bottles and any unused tablets to your doctor when requested
- You will need to come to clinic for scheduled visits as indicated in the table at the end of this leaflet. In this trial, each 28-day period (4 weeks) is called a cycle of treatment
- You will be given a Patient ID Card which provides emergency contact details for the trial team. You should carry this with you at all times and present it to a doctor if you are admitted to a hospital

For peer review only - http://bmjopen.bmj.com/site/about/guidelines.xhtml

Routine tests and procedures

The research team will perform the following routine tests at some or all of the visits to see how the trial medication is affecting your body (please see the table at the end of this information sheet which shows when each assessment or activity is done): physical exam, pregnancy testing if you are female and of child-bearing potential, vital signs, performance status, ECG, and lab safety tests. You will also be asked questions about your health and what medicines you are taking at the moment.

Extra tests and procedures

Additional research blood samples and echocardiograms will be taken at various points during the trial treatment period. A full list of tests and procedures is included at the end of this information sheet.

How long will I be in the trial?

Most participants are expected to receive trial treatment for at least 1 or 2 cycles, and initially the number of cycles of trial treatment is six (approximately 6 months). Following this, participants who are considered to be receiving clinical benefit can remain on trial and have continued access to trial treatment whilst the trial is still ongoing after approval from AstraZeneca. If one drug is permanently stopped (for example due to serious side effects), your trial doctor may decide whether it is in your best interest to continue taking the other drug. If the drugs are not helping you, or you are having serious side effects, or you simply do not wish to continue with the trial treatment, you can stop the trial and you can discuss alternative treatment options with your doctor.

After you stop taking the trial drug

When you have finished taking the trial medication, you will need to come in to the clinic for an end of treatment visit. You will undergo the same routine and extra tests and procedures as performed during the trial (outlined in the table at the end of this information sheet). These are done to see how the trial affected your health. Depending on how well you responded to treatment and if your hospital has the appropriate facilities you may be asked to give a sample of your bone marrow or blood using a hospital consent form to store for future research. This would enable researchers to obtain the samples in the future to look if the RAS pathway activating mutation that was identified in your leukaemia at the start of the trial is still there to learn more about how effective the treatment is.

In addition, you need to come in for a follow-up visit 28 days after the last trial medication was taken. Any side effects you may still have, and other medication you may be taking will be reviewed.

After the follow up visit at 28 days, you will be regularly reviewed as part of your ongoing care by your usual doctor.

Payments

You will not receive any money for taking part in this trial.

What will I have to do?

You will be required to:

- Attend the hospital as requested for assessments on your progress. You will need to have regular checks. Any side effects of this medicine may continue after you finish your treatment so it is important to report side effects promptly

For peer review only - http://bmjopen.bmj.com/site/about/guidelines.xhtml

SeluDex

- Take selumetinib capsules, commencing with one dose on Day 1 and twice daily from day 4 after pre-treatment assessments are completed. Your research nurse will give you guidance on this. Capsules should be taken whole, on an empty stomach (no food or drink other than water for 2 hours prior to dosing and 1 hour after dosing) and with a glassful of water
- Selumetinib doses should be taken approximately 12 hours apart at the same time points each day
- Take dexamethasone on day 2-4, 8-11, 15-18 and 22-25 of your first treatment cycle, day 1-4 of your second treatment cycle, then during the first five days of each subsequent cycle
- Have preventative antibiotic treatment
- Report any symptoms to the trial doctor when you attend for clinic appointments. In between clinic appointments, call the study team if you experience any severe side effects
- If you are on regular medication or herbal supplements, please make sure your oncology doctor and research nurse know about them before you start your treatment so that they can be checked for compatibility with your trial treatment
- After starting your trial treatment, if any new medication is required, it is important that you tell the doctor prescribing the new medication that you are in a clinical trial and are taking selumetinib and dexamethasone. A concomitant medication and restrictions card is provided to carry with you. It is also important that you tell the oncology doctor if you have been asked to start taking any new medications
- Do not have any vaccinations without your Doctor's approval
- If you are going to have surgery or any other procedures, tell your doctor or dentist you are taking part in this trial and inform them of the trial medication
- Bring all unused or remaining trial medication with you to each clinic visit
- Complete a diary to record when you took your trial medication, and bring the diary with you to your clinic appointments. The diary will contain more specific instructions about how to take your medication. A new diary will be provided to you at the beginning of each cycle of treatment
- Stop wearing contact lenses if you experience any mild to moderate eye symptoms. Also, you must not use any eye drops for the treatment of eye symptoms (unless agreed by your doctor). You are advised to talk to your doctor if you have any concerns
- Refrain from:
    - Eating or drinking large amounts of grapefruit/grapefruit juice (no more than a small glass of grapefruit juice (120ml) or half a grapefruit daily)
    - Taking any vitamin or herbal supplements
- Avoid excessive sun exposure, and use adequate sunscreen protection if sun exposure is anticipated
- You must not take Vitamin E vitamins or supplements
- It is recommended that you apply alcohol-free moisturiser to your skin before bedtime.

What are the possible side effects of the treatment?

You may have side effects while on the trial. All treatments can have side effects. Everyone taking part in the trial will be monitored carefully for any side effects, however doctors don't know all the side effects that may happen. Side effects may be mild or very serious. Your health care team may give you medicines to help lessen side effects or in some cases treatment may be delayed.

Participants in this trial will be at risk of known and unknown side effects of selumetinib and dexamethasone, as well as potential unknown risks due to the combination of the 2 drugs.

For peer review only - http://bmjopen.bmj.com/site/about/guidelines.xhtml

Participants with central nervous system (CNS) positive disease may be treated with intrathecal methotrexate (IT MTX) so may also be at risk of methotrexate induced neurotoxicity.

In the event of severe toxicity, and at the advice of your doctor, trial treatment may be interrupted temporarily until side effects have eased. Following an interruption, trial treatment could be restarted at a lower dose. If the side effects have not sufficiently reduced, trial treatment will be permanently stopped.

Selumetinib

Selumetinib is a new drug. More than 3,080 people have had the drug so far, and the drug has been very well tolerated. As it is a new drug, there may be side effects from taking selumetinib that doctors don't know about yet. The side effects already associated with selumetinib are listed below.

Common side effects of selumetinib:

More than 10 in every 100 people (10%) have one or more of these:

- Skin rash, which may be itchy in about 7 out of 10 people (70%). The majority of events are mild, but in some cases (around 10%) this can be more serious and require treatment
- Diarrhoea in about 5 out of 10 people (50%). It may occur within days of starting selumetinib treatment, and participants are advised to call the trial team as soon as possible so that treatment can be started promptly to help manage these side effects
- Feeling sick in about 5 out of 10 people (50%), or being sick in about 3 out of 10 people (30%). Participants are advised to call the trial team as soon as possible so that treatment can be started promptly to help manage these side effects
- Feeling tired or weak in around 4 out of 10 people (40%)
- Swelling of the hands or feet occurs in around 3 out of 10 people (30%)
- Shortness of breath in around 2 out of 10 people (20%). If you experience difficulty breathing or shortness of breath, call the trial team immediately
- Swelling of the face or extremities is commonly reported in patients receiving selumetinib
- Patients may also experience soreness or inflammation of the mouth, which may begin within days of starting treatment and usually occurs within the first month of treatment

Less common side effects of selumetinib:

Between 1 and 10 in every 100 people (1%-10%) have one or more of these:

- Swelling of the face, hands or feet
- Mouth sores, dry mouth
- High temperature
- Dry skin and soreness or infection in the skin around fingernails or toe nails. Sun exposure should be avoided when receiving selumetinib treatment
- Blurred vision – this has been mild or moderate in all reported previous cases. There is around a 5% chance that this may affect your daily living, such as using the telephone
- An increase in blood pressure
- Changes in the way your liver works
- Small decrease in cardiac (heart) function – this is usually mild
- Changes in your lungs – this will be carefully monitored by your doctor
- A lower than normal amount of blood cells that carry oxygen (anaemia)

Early information from clinical studies suggests that people of Asian origin may experience higher blood levels of selumetinib than non-Asian subjects. Higher levels of selumetinib in the blood may cause more side effects. Your trial doctor will discuss this information with you if it might affect your participation in the trial.

For peer review only

For peer review only - http://bmjopen.bmj.com/site/about/guidelines.xhtml

SeluDex

Dexamethasone

This drug has a known side effect profile as listed below:

- Increased appetite/weight gain
- Fluid retention
- An increase in blood pressure
- Excessive glucose in the bloodstream (hyperglycaemia)
- Steroid-induced diabetes mellitus
- Increased sensitivity to infection
- Disturbance of sleep patterns
- Low mood/irritability
- Acne type skin rashes/stretch marks
- Inflammation of the pancreas
- Death of bone tissue due to a lack of blood supply (avascular necrosis)

Intrathecal Methotrexate (IT MTX)

IT MTX may be given to trial participants in whom the leukaemia was found in the fluid taken by lumbar puncture, these are called "CNS positive participants". This drug has the potential for rare cases of neurotoxicity. Patients can experience acute toxicity (headache, nausea/vomiting, stiff neck) or delayed side effects (seizures, altered levels of consciousness and encephalopathy). It has been used for several decades in combination with intensive chemotherapy for the treatment of paediatric and adult ALL order to prevent CNS relapses. All participants will be regularly assessed for the occurrence of adverse effects, and CNS positive participants will also have regular full neurological examinations.

What are the possible benefits of taking part?

We cannot predict whether you will benefit directly from taking part in this trial. All participants in the trial will be closely monitored and supported by the research team. It is possible that your disease may respond to the trial treatment. We cannot guarantee that this treatment will help you, but the information we get from this trial will help us to improve the treatment of people with ALL in the future. You will not be paid for the use of your samples, even if the research does lead to the development of new medical products. Such developments take time and are unlikely to be critically dependent upon samples from any particular participant.

What are the possible disadvantages and risks of taking part?

It is possible that this combination of treatment will not work and you will still experience side effects.

Blood samples

You may experience a little discomfort when a needle is put into a vein or a port-a-cath to take blood samples. There may be slight pain, a small amount of bleeding, discolouration, or bruising at the site where the needle is inserted (but this will clear after a week or two). There is also a risk of infection or phlebitis (abnormal blood clot), but these rarely happen. Blood samples will be grouped together and taken with routine samples as much as possible to minimise the number of times a needle is inserted into your vein.

Bone marrow samples

A bone marrow test is taken during your screening visit and again after 4 weeks of treatment to see how well you have responded to the treatment. This is used to look for the amount of leukaemia that you have left and is done by microscope examination and in addition a minimal residual disease (MRD) test is performed on the screening sample and on future samples for participants who have responded well. The site of the bone marrow examination might be

For peer review only - http://bmjopen.bmj.com/site/about/guidelines.xhtml

SeluDex

painful for a few days afterwards. A bone marrow examination may be performed under a general anaesthetic.

Lumbar puncture

A lumbar puncture is performed during screening and might get repeated in case the leukaemia is found in the fluid taken during the initial lumbar puncture. In this case your physician might want to treat you with IT MTX (see paragraph on side effects of treatment). A lumbar puncture may be performed under a general anaesthetic together with a bone marrow examination. Side effects of the procedure include headaches, local discomfort, bruising, bleeding and a low risk of infection.

Possible harm to the unborn child and pregnancy

Selumetinib may have a harmful effect on a baby developing in the womb when administered to a pregnant woman. Reproductive toxicology data indicate that selumetinib has adverse effects on embryo foetal development and survival, at dose levels that do not induce maternal toxicity in animal models. It is therefore essential that both men and women taking the trial treatment must agree to use a reliable form of contraception during the trial, e.g. oral contraceptive and condom, intra-uterine device (IUD) such as Mirena Coil and condom, diaphragm with spermicide and condom. Total abstinence is only an acceptable method of contraception when it is your usual and preferred lifestyle choice. You should continue the use of adequate contraception for at least 12 weeks after the treatment has finished.

Information for women

You should not take part in this trial if you are pregnant, breastfeeding or may become pregnant during the trial period. If you are female and of childbearing potential, you will have a pregnancy test during screening and then monthly whilst on trial. This may be repeated if pregnancy is suspected whilst you are on the trial. You must agree to use two reliable forms of contraception (as detailed above) for two weeks prior to entering the trial, during the trial, and for at least 12 weeks after the treatment has finished.

If you do become pregnant during the course of the trial, please tell your trial doctor immediately. The pregnancy will need to be monitored and information about the outcome of your pregnancy will be collected from your medical notes and those of your baby.

Information for men

If you have a partner who is pregnant or who could become pregnant you must agree to use two reliable forms of contraception (as detailed above) for two weeks prior to entering the trial, during the trial, and for at least 12 weeks after the treatment has finished.

If your partner becomes pregnant whilst you are on the trial treatment, please tell your doctor immediately. We would also like to collect information about the outcome of the pregnancy. If your partner becomes pregnant during the trial, we will ask your partner to consent to this monitoring, if she is happy to do so.

What are the alternatives for treatment?

It is important that you discuss any possible treatment alternatives with your doctors before deciding whether to enter this trial. If you decide not to participate in this trial, you will receive standard or palliative care, or may enter another early phase trial (if available).

What happens when the research trial stops?

The trial treatment will not be available after the end of the trial. You will receive trial treatment should it be of benefit only for the duration of the trial. Initially this will be for 6 cycles (approximately 6 months). After this if treatment is considered to be of benefit to you then you may remain on treatment by continuing on the trial whilst it is ongoing following approval from

For peer review only - http://bmjopen.bmj.com/site/about/guidelines.xhtml

AstraZeneca. The schedule of assessments for treatment continuation will follow cycles 3-6 (see table at the end of this document). At the end of the research trial, or if you withdraw from the trial before it ends, your trial doctor will assess your symptoms and discuss your options and prescribe further alternative treatment if appropriate. If the academic body sponsoring the research trial decides to stop the trial before it has finished, your trial doctor will explain the reasons why and arrange appropriate care for you.

What if there is a problem?

Any complaint about the way you have been treated during the trial or any possible harm you might suffer will be addressed. The detailed information on this is given in part 2 of this leaflet.

Will my taking part in the trial be kept confidential?

Yes. We will follow ethical and legal practice, and all information about you will be handled in confidence. The details are included in Part 2 of this leaflet.

This completes Part 1 of the Participant Information Sheet

If the information in Part 1 has interested you, and you are considering participation, please read the additional information in Part 2 before making any decision

For peer review only

For peer review only - http://bmjopen.bmj.com/site/about/guidelines.xhtml

PART 2

What if relevant new information becomes available?

Sometimes during the course of a research trial, new information becomes available about the disease or drug that is being studied. If this happens, your doctor will tell you about it and discuss with you whether you want to continue on the trial. If you decide to withdraw, your doctor will make arrangements for your care to continue in a different way. If you decide to continue in the trial you may be asked to sign an updated consent form. If the trial is stopped for any other reason, we will tell you and arrange your continuing care.

What will happen if I don't want to carry on with the trial?

You are free to withdraw from the trial at any time. You do not have to give a reason and your future treatment will not be affected. Your doctor will discuss your treatment options with you and will offer you the most suitable treatment available. However, if you choose to withdraw entirely from the trial, we would still like to use the information we have collected about you up until withdrawal. If you just want to stop trial treatment but are happy to be seen in accordance with the schedule specified in this information leaflet please let your local trial team know. Alternatively we would like to ask your permission for your hospital to continue to send information about your progress to the SeluDex Trial Office. This is information that is routinely taken and you will not need to do anything extra. Any stored blood or tissue samples collected specifically for this trial up until the point of withdrawal will be retained and analysed. With your permission, the SeluDex Trial Office would also like to obtain information about your progress from the cancer registries and national databases (for example NHS Digital).

What if there is a problem?

You will be closely monitored both during and after treatment, and any side effects you experience will be treated.

Complaints: If you have a concern about any aspect of this trial, you should ask to speak with the trial doctor who will do their best to answer your questions (see contact number at the end of this form). If you remain unhappy and wish to complain formally, you can do this through the NHS Complaints Procedure. Details can be obtained from the hospital.

Harm: Every care will be taken in the course of this clinical trial. However, in the unlikely event that you are harmed and this is due to someone's negligence then you may have grounds for legal action for compensation against the trial sponsor (University of Birmingham) or the NHS Trust treating you, but you may have to pay your legal costs. NHS Trust and Non-Trust Hospitals have a duty of care to patients treated, whether or not the patient is taking part in a clinical trial and the normal NHS complaints mechanisms will still be available to you. The sponsor of the trial does not hold insurance against claims for compensation for injury caused by participation in this trial and they cannot offer any indemnity.

Participants may also be able to claim compensation for injury caused by participation in this clinical trial without the need to prove negligence on the part of the sponsor or another party. You should discuss this possibility with your trial doctor in the same way as above.

Regardless of this, if you wish to complain, or have any concerns about any aspect of the way you have been approached or treated by members of staff or about any side effects (adverse events) you may have experienced due to your participation in the clinical trial, the normal NHS complaints mechanisms are available to you. Please ask your trial doctor if you would like more information on this. Details can also be obtained from the Department of Health website: http://www.dh.gov.uk.

For peer review only - http://bmjopen.bmj.com/site/about/guidelines.xhtml

SeluDex

What will happen to the samples that I give?

Routine safety blood and urine samples will be analysed at your hospital laboratory throughout the course of the trial to see how the trial medication is affecting your body (pharmacodynamic analysis) and how your body processes the trial medication (pharmacokinetic analysis).

Throughout the course of the trial, additional samples will be taken for the purpose of the research. These samples will be labelled and identified with a unique sample ID number, your trial number and sometimes date of birth and initials. Samples will be sent to three different locations:

- Samples collected for pharmacokinetic analysis will be sent to Newcastle University
- Samples for pharmacodynamic analysis will be initially stored at the biobank at the University of Birmingham and and may be subsequently analysed at Newcastle University and at AstraZeneca
- Minimal Residual Disease (MRD) samples, if not tested routinely at your hospital, will be sent to University College, London for analysis.

Any samples remaining at the end of the trial may be stored and used for future ethically approved research which may involve genetic analysis, animal or other laboratory work at commercial or private institutions, and which may take place in the UK or overseas. If your samples are used, they will be anonymised.

Will any genetic tests be done?

Prior to being approached by your doctor to consider taking part in this trial, you will have consented to give a sample to assess whether your disease is positive for a RAS pathway activating mutation in its genetic make up.

What will happen to the results of the research trial?

At the end of the trial, the information collected will be analysed and published in recognised medical journals. You will not be identified in any report or publication. The results will help doctors to decide how to treat relapsed ALL in the future. Participants taking part in this trial can find out about the results from their trial doctor once the results have been published. The results will also be available on the Cancer Research UK website and the CRCTU website (http://www.birmingham.ac.uk/crctu). We expect the first results to be available in approximately 3 years.

Who is organising and funding the research?

The trial is an investigator-initiated and investigator-led trial, and is being carried out by a network of doctors across the UK and internationally. The trial is sponsored by the University of Birmingham and is being coordinated by the Cancer Research UK Clinical Trials Unit (CRCTU). Financial support for the trial is provided by the charity Cancer Research UK and from a pharmaceutical company called AstraZeneca. Your doctor will not receive any payments for talking with you about, or recruiting you into, this research trial.

Who has reviewed the trial?

All research in the NHS is looked at by independent group of people called a Research Ethics Committee to protect your safety, rights, wellbeing and dignity. This trial has been reviewed and given favourable opinion by a Research Ethics Committee, and by the local Research and Development department at your hospital's Trust. Whilst the trial is ongoing, the results will be reviewed by a Trial Safety Committee (TSC) to ensure that it is appropriate to continue with the trial.

For peer review only - http://bmjopen.bmj.com/site/about/guidelines.xhtml

Will my taking part in this trial be kept confidential?

All your details and information collected about you for this research trial will be subject to the General Data Protection Regulation (GDPR) and the Data Protection Act 2018 and will be kept totally confidential. Your initials, date of birth, ethnicity, hospital number and NHS number (or Community Health Index (CHI) in Scotland) will be supplied to the Cancer Research UK Clinical Trials Unit when you are entered in to the trial along with a copy of your consent form. In routine communication, your hospital the Cancer Research UK Clinical Trials Unit will refer to you only by a unique trial number allocated to you, and/or your date of birth and initials. All information about you will be securely stored, in both electronic format and paper form by the researchers, and will only be accessible by authorised personnel.

Occasionally, we may need to check your medical records to make sure that the information provided about you is accurate. This will be done either by clinical staff or by designated trial personnel. It may also be necessary to allow authorised personnel from government regulatory agencies, the sponsor of the trial (the group legally responsible for the conduct of the trial) and/or NHS bodies to have access to information about you. This is for your protection, and is to ensure that the research trial is being conducted to the highest possible standards.

If you agree to take part in this trial, then, in addition to the authorised personnel who review the main trial information, medical information about you may be passed on to researchers for future medical research. The information that they will be given for their work relates to your medical condition and treatment only and will not be directly linked to your identity. Researchers will not be able to contact you directly about their research in the future and you will not be identified in any reports or publications resulting from the study.

*At <insert hospital name> this trial will be managed by the <insert name> Clinical Trials Unit at <insert location> where the clinical team looking after you reside. Therefore it will be necessary for identifiable information to be transferred to and held by the Clinical Trials Unit. This includes a copy of the consent form which you will sign if you agree to take part in the trial. This information will remain confidential at all times.

*Delete as appropriate

The University of Birmingham is the sponsor for this study based in the United Kingdom. We will be using information from you and/or your medical records in order to undertake this study and will act as the data controller for this study. This means that we are responsible for looking after your information and using it properly. The University of Birmingham will keep identifiable information about you for up to 25 years after the study has finished.

Your rights to access, change, or move your information is limited, as we need to manage your information in specific ways in order for the research to be reliable and accurate. If you withdraw from the study, we will keep the information about you that we have already obtained. To safeguard your rights, we will use the minimum personally-identifiable information possible.

You can find out more about how we use your information in our Privacy Policy on our website (www.birmingham.ac.uk/crctu).

The NHS via your hospital(s) will collect information from your medical records for this research study in accordance with our instructions. Your hospital(s) will use your name, NHS number and contact details to contact you about the research study, and make sure that relevant information about the study is recorded for your care, and to oversee the quality of the study.

The NHS will keep identifiable information about you from this study for up to 25 years after the study has finished.

For peer review only - http://bmjopen.bmj.com/site/about/guidelines.xhtml

Involvement of the General Practitioner/Family Doctor (GP)

As this trial requires you to take a drug which may cause side-effects, it is important that your General Practitioner (GP) is informed that you are taking part in the trial. With your permission your trial doctor will notify your GP that you intend to participate in the trial. In addition, we may ask them to provide information on your progress. If we do need to contact your GP for any follow-up information, we will need to use your full name in our correspondence.

For peer review only

For peer review only - http://bmjopen.bmj.com/site/about/guidelines.xhtml

SeluDex

Further information and contact details

If you have any further questions or concerns about your disease or this clinical trial please feel free to discuss them with the doctors and nurses looking after you before deciding to take part in the trial or at any time during the trial. Before you make a decision, you may want to discuss the trial with your family and friends and with your family doctor.

You may also find it helpful to contact the following organisations:

Cancer Research UK

An information service about cancer and cancer research studies hosted by Cancer Research UK

www.cancerresearchuk.org/about-cancer/

Tel: 0808 800 40 40

Macmillan Cancer Support

An independent patient advisory group

Macmillan Cancer Support, 89 Albert Embankment, London, SE1 7UQ, UK

www.macmillan.org.uk

Tel: 0808 808 0000

Children's Cancer and Leukaemia Group

A children's cancer charity for those involved in the treatment and care of children with cancer

CCLGroup, Clinical Sciences Building, Leicester Royal Infirmary, Leicester LE2 7LX

http://www.cclg.org.uk/

Tel: 0116 252 5858

Please take as much time as you need to make a decision and then let your doctor know what you have decided so that your treatment can be arranged.

If you have any further questions, you are very welcome to contact the trial doctor who is leading this trial, whose contact details are provided below:

Local Investigator: ……………………………………………………………………..

Trial Nurse: ……………………………………………………………………..

Trial Coordinator: ……………………………………………………………………..

Emergency Contact Number: ……………………………………………………………………..

Thank you for reading this information sheet. If you decide to take part you will be given a copy of this information sheet and your signed consent form.

For peer review only

For peer review only - http://bmjopen.bmj.com/site/about/guidelines.xhtml

Assessments at Each Visit

	Prior to start of treatment	Cycle 1	Cycle 2	Day 1 of each following cycle	End of Treatment	28 days follow up
**Laboratory Tests**						
Blood tests	✓	✓ (Days 1, 8, 15, 22 & 28)	✓ (Days 1 & 15)	✓	✓	✓
Urine sample	✓	✓ (Days 1, 8, 15 & 22)	✓ (Day 1)	✓	✓	✓
Pregnancy test [a]	✓		✓	✓	✓	✓
**Disease Assessment**						
Bone marrow testing/MRD Sample	✓	✓ (Day 28)		✓ (Cycle 4 & 6 only)	✓	
Lumbar puncture	✓	✓ (Day 28 if CNS positive)				
**Treatment**						
Selumetinib		✓ (Day 1 and continuously from day 4)				
Dexamethasone		✓ (Day 2-4, 8-11, 15-18 & 22-25)	✓ (Day 1-4)	✓ (Day 1-5)		
Preventative Antibiotics		Throughout treatment				
**Research Samples**						
Blood samples		✓ (Day 1 & 4)	✓ (Day 1)		✓	
**Other Assessments and Activities**						
Medical history	✓					
Physical examination	✓	✓ (Day 1, 8, 15 & 22)	✓ (Day 1 & 15)	✓	✓	✓
Performance Status	✓	✓ (Day 1, 8, 15 & 22)	✓ (Day 1)	✓	✓	✓
Vital signs (incl. weight)	✓	✓ (Day 1, 8, 15 & 22)	✓ (Day 1)	✓	✓	✓
Growth Chart (Height) – 16-18yrs only	✓			✓ (Cycle 4 only)	✓	✓
ECG	✓	✓ (Day 1)	✓ (Day 1)	✓	✓	✓
ECHO	✓	✓ (Day 28)		Additional tests may be necessary if your doctor is concerned about side effects		
Eye exam	✓	Additional tests may be necessary if your doctor is concerned about side effects				
Medication/side effects monitoring	✓ (Done at every clinic visit from screening through to 28-day follow up visit)					

[a] Female participants of childbearing potential only

For peer review only - http://bmjopen.bmj.com/site/about/guidelines.xhtml

SeluDex

Supplementary Appendix 4

SeluDex Trial Schedule of Events

	**Screening**[1] (Within 14 days of Trial Entry)	**C1/Day 1**[2]	**C1/Day 2**	**C1/Day 4**	**C1/Day 8**[3] (± 2 days)	**C1/Day 15**[3] (± 2 days)	**C1/Day 22**[3] (± 2days)	**C1/Day 28**[4] (- 3 days during Phase II only)	**C2/Day 1**[5]	**C2/Day 15**[3] (± 2 days)	**C3/Day 1**[6] (- 2 days)	**C4/Day 1**[6, 7] (- 2 days)	**C5/Day 1**[6] (- 2 days)	**C6/Day 1**[6, 7] (- 2 days)	**End of Treat-ment Visit**[8] (+ 14 days)	**28 day Follow Up Visit**[9] (+ 2 days)
Informed Consent[a]	x															
RAS-pathway mutation testing[b]	x*														x	
Medical history[c]	x															
Performance Status (ECOG, Lansky, or Karnofsky scale)	x	x			x	x	x		x (- 1 day)		x	x	x	x		x
Vital signs[d]	x	x			x	x	x		x (- 1 day)		x	x	x	x	x	x
Weight (kg)	x	x (-3 days)			x	x	x		x (- 1 day)		x	x	x	x	x	x
Body Surface Area	x	x (-3 days)							x (- 1 day)		x	x	x	x		
Physical examination[e]	x	x			x	x	x		x (- 1 day)	x	x	x	x	x		x
Serum chemistry[f]	x	x (-3 days)			x	x	x		x (- 1 day)	x	x	x	x	x		x
Full blood count & peripheral blood blast count[g]	x	x (-3 days)			x	x	x	x		x	x	x	x	x	x	x
ECG[h]	x	x							x (- 1 day)		x	x	x	x		x
Urinalysis (dipstick)	x	x			x	x	x		x (- 1 day)		x	x	x	x		x
Height (cm)	x															
Bone marrow aspirate[i]	x							x				x (- 3 days)		x (- 3 days)	x	
Cytogenetic analysis & Immunophenotyping	x															
MRD sample [j]	x							x				x (- 3 days)		x (- 3 days)	x	
Lumbar puncture[k]	x							x								
Serological studies for HBV, HCV, HIV	x															
Pregnancy test[l]	x (-7 days)								x (-1 day)		x	x	x	x	x	x
ECHO[m]	x							x (- 3 days)								
Ophthalmological exam[n]	x															
Adverse events		x	x	x	x	x	x	x	x	x	x	x	x	x	x	x
Concomitant medications	x	x	x	x	x	x	x	x	x	x	x	x	x	x	x	x
Growth Chart (Group P only) [o]	x											x			x	x
Pharmacokinetic samples (blood) [p]		x	x	x					x							

For peer review only - http://bmjopen.bmj.com/site/about/guidelines.xhtml

For peer review only

SeluDex

	Screening[1] (Within 14 days of Trial Entry)	C1/Day 1[2]	C1/Day 2	C1/Day 4	C1/Day 8[3] (± 2 days)	C1/Day 15[3] (± 2 days)	C1/Day 22[3] (± 2days)	C1/Day 28[4] (- 3 days during Phase II only)	C2/Day 1[5]	C2/Day 15[3] (± 2 days)	C3/Day 1[6] (- 2 days)	C4/Day 1[6,7] (- 2 days)	C5/Day 1[6] (- 2 days)	C6/Day 1[6,7] (- 2 days)	End of Treat-ment Visit[8] (+ 14 days)	28 day Follow Up Visit[9] (+ 2 days)
Pharmacodynamic samples - RNA & Fixed (blood)[q]		x	x	x											x	
Patient Diary (dispense and review as appropriate)[r]		x							x		x	x	x	x		
Selumetinib dispensing		x							x		x	x	x	x		
Dexamethasone dispensing			x						x		x	x	x	x		
Fluoroquinolone prophylaxis dispensing		x							x							
Co-trimoxazole prophylaxis dispensing		x							x		x	x	x	x		

Initially trial treatment is for 6 cycles (approximately 6 months) however following this participants who are considered to be receiving clinical benefit can remain on trial and have continued access to selumetinib whilst the trial is open following approval from AstraZeneca. The schedule of assessments for treatment continuation should follow cycles 3-6.

[1] Any assessments done as standard care do not require informed consent and may be provided as screening data if conducted within 14 days of trial entry unless otherwise stated

[2] Weight, Body Surface Area, Serum Chemistry, FBC and peripheral blood blast count may be performed within the 3 days prior to cycle 1 day 1

[3] Visit may occur within the 2 days prior to or after the scheduled visit date

[4] During Phase I bone marrow aspirate and all associated assessments at this visit must occur on cycle 1 day 28 of treatment except for the ECHO which may be performed within the 3 days prior to the scheduled visit date. During Phase II this visit and all associated assessments may occur within the 3 days prior to the scheduled visit date

[5] Performance status, vital signs, weight, BSA, physical examination, serum chemistry, FBC, ECG and urinalysis (dipstick) may be performed within the 1 day prior to the scheduled visit date

[6] Visit may occur within the 2 days prior to the scheduled visit date

[7] Bone marrow aspirate and MRD sample may be performed within the 3 days prior to the scheduled visit date

[8] The End of Treatment assessment visit should be performed within 14 days of the patient's last administration of selumetinib. Should the patient discontinue following a recent bone marrow aspirate visit then a repeat bone marrow aspirate for end of treatment is not required at the discretion of the investigator

[9] Patients will be followed up for 28 days after the last dose of selumetinib, with a clinic visit taking place on day 28. Visit may occur within the 2 days after the scheduled visit date

* RAS-pathway mutation result date does not have to be within 14 days of trial entry but must be confirmed using a sample from the current relapse/disease

[a] Written informed consent must be received before any trial specific procedures occur

[b] Sample from current relapse/disease must confirm presence of a RAS-pathway activating mutation - NRAS, KRAS, FLT3, PTPN11, cCBL, NF1, BRAF, IKZF2, IKZF3, IL7Rα or JAK1. Results from trial office approved local molecular testing are accepted for all of these mutations. Testing for NRAS, KRAS, FLT3, PTPN11 and cCBL mutations can be done centrally by Northern Genetics Services (Previously NewGene Limited) in Newcastle, UK – refer to the laboratory manual for further information. Optional end of treatment sample to be collected from non-responders and stored at sites with local biobank facilities and appropriate ethical approval using local consent to assess clonal evolution of RAS-pathway wildtype ALL

[c] Medical history – including demographics, prior treatment, allergy history, discuss contraception

For peer review only - http://bmjopen.bmj.com/site/about/guidelines.xhtml
For peer review only

SeluDex

[d] Blood pressure, pulse measurement, temperature, and respiratory rate to be performed with the patient sitting for 5 minutes prior to the evaluation

[e] Physical examination includes complete review of systems, physical examination of pertinent organ systems, and neurological exam

[f] Includes: Glucose, LDH and creatine kinase (CK) at screening only. Creatinine, Sodium, Potassium, Total Calcium, Total Protein, Phosphate, Magnesium, Urea, Total Bilirubin, AST or ALT ALP and albumin at all timepoints

[g] Full Blood Count with differential count and peripheral blood blast count as an additional request test at all timepoints

[h] Repeat ECG as clinically indicated

[i] Bone marrow aspirate at screening (peripheral blood can be used in the case of a dry tap if peripheral WCC > $50x10^9$/L – screening only); cycle 1 day 28, cycle 4 day 1 and cycle 6 day 1. For any patients who continue treatment after cycle 6, a bone marrow aspirate should be performed every other cycle.

[j] An MRD sample will be collected from the bone marrow aspirate at screening for all patients. At subsequent timepoints an MRD sample should be collected each time a BM aspirate is performed for patients who have achieved at least a CRi and where a marker was identified at screening. Group A: MRD samples will be sent to UCL for analysis. Group P: MRD will be analysed as part of standard of care

[k] A Lumbar puncture to assess CNS status should be done at Screening for all patients and at cycle 1 day 28 for CNS positive patients only

[l] Women of childbearing potential will require a negative pregnancy test (serum or urine) prior to registration and within seven days prior to study drug administration, then monthly

[m] An ECHO should be performed at screening. Repeat on cycle 1 day 28 (or within the 3 days prior) and as clinically indicated. (Group A: LVEF, LVEDV & LVESV, Group P: SF)

[n] Repeat as clinically indicated

[o] Group P only. Body height (standing or lying length where age-appropriate) will be measured. Where possible, a stadiometer should be used. Where this may be difficult in particularly young children, standard measurement methods may be used.

[p] Pharmacokinetic samples at the following time points:

- Cycle 1 day 1: pre first selumetinib dose, then at 30 minutes, 1 hour, 2 hours, 4 hours, 6 hours and 24 hours after the first dose (pre cycle 1 day 2 dexamethasone dose)
- Cycle 1 day 4: pre first selumetinib/dexamethasone dose, then at 30 minutes, 1 hour, 2 hours, 4 hours, 6 hours after the first dose
- Cycle 2 day 1: pre first selumetinib/dexamethasone dose, then at 30 minutes, 1 hour, 2 hours, 4 hours, 6 hours after the first dose

[q] Pharmacodynamic blood samples at the following time points (only if WCC $>10x10^9$/l). PD-RNA samples - all sites. PD-Fixed samples – if facilities available at site as agreed at initiation:

- Cycle 1 day 1: pre first selumetinib dose, then at 1 hour, 4 hours, 6 hours and 24 hours after the first dose
- Cycle 1 day 4: pre first selumetinib/dexamethasone dose, then at 1 hour, 4 hours, 6 hours after the first dose
- End of Treatment visit (one sample only)

[r] All patients/parents/legal guardians will be required to complete a diary, which must be returned to the clinic for checking at each visit. The research nurse should complete the relevant sections including recording the AM and PM doses and the dates they are to be taken on the diary. Patients/parents/legal guardians should be instructed to record daily time of administration of the study drugs in the diary. If a dose is missed, the reason must be noted in the diary by the patient/parent/legal guardian. The patient diary will then be reviewed by the research nurse at the end of each cycle. Patients/parents/legal guardians should be advised to return any unused IMP in the original bottles, in addition to returning any empty bottles.

For peer review only - http://bmjopen.bmj.com/site/about/guidelines.xhtml

Supplementary Appendix 5

Adverse Event

Any untoward medical occurrence in a patient or clinical trial subject administered a medicinal product and which does not necessarily have a causal relationship with this treatment.

Comment:

An AE can therefore be any unfavourable and unintended sign (including abnormal laboratory findings), symptom or disease temporally associated with the use of an investigational medicinal product, whether or not related to the investigational medicinal product (IMP).

Adverse Reaction

All untoward and unintended responses to an IMP related to any dose administered.

Comment:

An AE judged by either the reporting Investigator or Sponsor as having causal relationship to the IMP qualifies as an AR. The expression reasonable causal relationship means to convey in general that there is evidence or argument to suggest a causal relationship.

Serious Adverse Event

Any untoward medical occurrence or effect that at any dose:

Results in death

Is life threatening*

Requires hospitalisation** or prolongation of existing inpatients' hospitalisation

Results in persistent or significant disability or incapacity

Is a congenital anomaly/birth defect

Or is otherwise considered medically significant by the Investigator***

Comments:

The term severe is often used to describe the intensity (severity) of a specific event. This is not the same as serious, which is based on patients/event outcome or action criteria.

* Life threatening in the definition of an SAE refers to an event in which the patient was at risk of death at the time of the event; it does not refer to an event that hypothetically might have caused death if it were more severe.

**Hospitalisation is defined as an unplanned, formal inpatient admission, even if the hospitalisation is a precautionary measure for continued observation. Thus, hospitalisation for protocol treatment (e.g., line insertion), elective procedures (unless brought forward because of worsening symptoms) or for social reasons (e.g., respite care) are not regarded as an SAE.

For peer review only

*** Medical judgment should be exercised in deciding whether an AE is serious in other situations. Important AEs that are not immediately life threatening or do not result in death or hospitalisation but may jeopardise the subject or may require intervention to prevent one of the other outcomes listed in the definition above, should be considered serious.

Serious Adverse Reaction

An Adverse Reaction which also meets the definition of a Serious Adverse Event.

Suspected Unexpected Serious Adverse Reaction

A SAR that is unexpected i.e., the nature, or severity of the event is not consistent with the applicable product information.

A SUSAR should meet the definition of an AR, UAR and SAR.

Unexpected Adverse Reaction

An AR, the nature or severity of which is not consistent with the applicable product information (e.g., Investigator Brochure for an unapproved IMP or (compendium of) Summary of Product Characteristics (SPC) for a licensed product).

When the outcome of an AR is not consistent with the applicable product information the AR should be considered unexpected.

For peer review only

SPIRIT

STANDARD PROTOCOL ITEMS: RECOMMENDATIONS FOR INTERVENTIONAL TRIALS

SPIRIT 2013 Checklist: Recommended items to address in a clinical trial protocol and related documents*

Section/item	Item No	Description
**Administrative information**		
Title	1	Descriptive title identifying the study design, population, interventions, and, if applicable, trial acronym<br>Page 1; lines 1-4
Trial registration	2a	Trial identifier and registry name. If not yet registered, name of intended registry<br>Page 2; lines 57-58
	2b	All items from the World Health Organization Trial Registration Data Set<br>Supplementary Appendix 1
Protocol version	3	Date and version identifier<br>Page 12; line 442
Funding	4	Sources and types of financial, material, and other support<br>Page 13; lines 466-472
Roles and responsibilities	5a	Names, affiliations, and roles of protocol contributors<br>Names and affiliations on page 1; lines 5-26<br>Names and role on page 12; lines 454-464
	5b	Name and contact information for the trial sponsor<br>Page 10; lines 359-360
	5c	Role of study sponsor and funders, if any, in study design; collection, management, analysis, and interpretation of data; writing of the report; and the decision to submit the report for publication, including whether they will have ultimate authority over any of these activities<br>Page 13; lines 474-476
	5d	Composition, roles, and responsibilities of the coordinating centre, steering committee, endpoint adjudication committee, data management team, and other individuals or groups overseeing the trial, if applicable (see Item 21a for data monitoring committee)

For peer review only

		Page 10; lines 359-375
**Introduction**		
Background and rationale	6a	Description of research question and justification for undertaking the trial, including summary of relevant studies (published and unpublished) examining benefits and harms for each intervention<br><br>Page 3; lines 69-95
	6b	Explanation for choice of comparators<br><br>Page 3; line 102
Objectives	7	Specific objectives or hypotheses<br><br>Page 3; lines 96-98
Trial design	8	Description of trial design including type of trial (eg, parallel group, crossover, factorial, single group), allocation ratio, and framework (eg, superiority, equivalence, noninferiority, exploratory)<br>Page 3; lines 101-104
**Methods: Participants, interventions, and outcomes**		
Study setting	9	Description of study settings (eg, community clinic, academic hospital) and list of countries where data will be collected. Reference to where list of study sites can be obtained<br><br>Page 3; lines 103-104 and Page 11; line 338
Eligibility criteria	10	Inclusion and exclusion criteria for participants. If applicable, eligibility criteria for study centres and individuals who will perform the interventions (eg, surgeons, psychotherapists)<br><br>Page 4; lines 116-128
Interventions	11a	Interventions for each group with sufficient detail to allow replication, including how and when they will be administered<br><br>Page 4; lines 141-168
	11b	Criteria for discontinuing or modifying allocated interventions for a given trial participant (eg, drug dose change in response to harms, participant request, or improving/worsening disease)<br><br>Page 5; lines 181-197
	11c	Strategies to improve adherence to intervention protocols, and any procedures for monitoring adherence (eg, drug tablet return, laboratory tests)<br><br>n/a

For peer review

For peer review only - http://bmjopen.bmj.com/site/about/guidelines.xhtml

	11d	Relevant concomitant care and interventions that are permitted or prohibited during the trial<br><br>Page 6; lines 199-201
Outcomes	12	Primary, secondary, and other outcomes, including the specific measurement variable (eg, systolic blood pressure), analysis metric (eg, change from baseline, final value, time to event), method of aggregation (eg, median, proportion), and time point for each outcome. Explanation of the clinical relevance of chosen efficacy and harm outcomes is strongly recommended<br><br>Page 6; lines 203-241
Participant timeline	13	Time schedule of enrolment, interventions (including any run-ins and washouts), assessments, and visits for participants. A schematic diagram is highly recommended (see Figure)<br><br>Supplementary Appendix 4
Sample size	14	Estimated number of participants needed to achieve study objectives and how it was determined, including clinical and statistical assumptions supporting any sample size calculations<br><br>Page 8; lines 262-313
Recruitment	15	Strategies for achieving adequate participant enrolment to reach target sample size<br><br>n/a

Methods: Assignment of interventions (for controlled trials)

Allocation:

Sequence generation	16a	Method of generating the allocation sequence (eg, computer-generated random numbers), and list of any factors for stratification. To reduce predictability of a random sequence, details of any planned restriction (eg, blocking) should be provided in a separate document that is unavailable to those who enrol participants or assign interventions<br><br>n/a
Allocation concealment mechanism	16b	Mechanism of implementing the allocation sequence (eg, central telephone; sequentially numbered, opaque, sealed envelopes), describing any steps to conceal the sequence until interventions are assigned<br><br>n/a
Implementation	16c	Who will generate the allocation sequence, who will enrol participants, and who will assign participants to interventions

For peer review only - http://bmjopen.bmj.com/site/about/guidelines.xhtml

Blinding (masking)	17a	Who will be blinded after assignment to interventions (eg, trial participants, care providers, outcome assessors, data analysts), and how<br><br>n/a
	17b	If blinded, circumstances under which unblinding is permissible, and procedure for revealing a participant's allocated intervention during the trial<br><br>n/a
**Methods: Data collection, management, and analysis**		
Data collection methods	18a	Plans for assessment and collection of outcome, baseline, and other trial data, including any related processes to promote data quality (eg, duplicate measurements, training of assessors) and a description of study instruments (eg, questionnaires, laboratory tests) along with their reliability and validity, if known. Reference to where data collection forms can be found, if not in the protocol<br><br>Page 10; lines 339-357
	18b	Plans to promote participant retention and complete follow-up, including list of any outcome data to be collected for participants who discontinue or deviate from intervention protocols<br><br>n/a
Data management	19	Plans for data entry, coding, security, and storage, including any related processes to promote data quality (eg, double data entry; range checks for data values). Reference to where details of data management procedures can be found, if not in the protocol<br><br>Page 10; lines 339-357
Statistical methods	20a	Statistical methods for analysing primary and secondary outcomes. Reference to where other details of the statistical analysis plan can be found, if not in the protocol<br><br>Page 6; lines 212-242
	20b	Methods for any additional analyses (eg, subgroup and adjusted analyses)<br><br>Page 6; lines 234-236
	20c	Definition of analysis population relating to protocol non-adherence (eg, as randomised analysis), and any statistical methods to handle missing data (eg, multiple imputation)<br><br>Page 8; lines 263-269

For peer review only - http://bmjopen.bmj.com/site/about/guidelines.xhtml

Methods: Monitoring

Data monitoring	21a	Composition of data monitoring committee (DMC); summary of its role and reporting structure; statement of whether it is independent from the sponsor and competing interests; and reference to where further details about its charter can be found, if not in the protocol. Alternatively, an explanation of why a DMC is not needed<br><br>Page 10; lines 360-376
	21b	Description of any interim analyses and stopping guidelines, including who will have access to these interim results and make the final decision to terminate the trial<br><br>Page 9; line 314
Harms	22	Plans for collecting, assessing, reporting, and managing solicited and spontaneously reported adverse events and other unintended effects of trial interventions or trial conduct<br><br>Page 10; lines 372-374
Auditing	23	Frequency and procedures for auditing trial conduct, if any, and whether the process will be independent from investigators and the sponsor<br><br>n/a

Ethics and dissemination

Research ethics approval	24	Plans for seeking research ethics committee/institutional review board (REC/IRB) approval<br><br>Page 12; lines 439-443
Protocol amendments	25	Plans for communicating important protocol modifications (eg, changes to eligibility criteria, outcomes, analyses) to relevant parties (eg, investigators, REC/IRBs, trial participants, trial registries, journals, regulators)<br><br>n/a
Consent or assent	26a	Who will obtain informed consent or assent from potential trial participants or authorised surrogates, and how (see Item 32)<br><br>Page 4; line 131-136
	26b	Additional consent provisions for collection and use of participant data and biological specimens in ancillary studies, if applicable<br><br>n/a

For peer review only - http://bmjopen.bmj.com/site/about/guidelines.xhtml

Confidentiality	27	How personal information about potential and enrolled participants will be collected, shared, and maintained in order to protect confidentiality before, during, and after the trial<br><br>Page 10; lines 378-385
Declaration of interests	28	Financial and other competing interests for principal investigators for the overall trial and each study site<br><br>Page 13; lines 479-480
Access to data	29	Statement of who will have access to the final trial dataset, and disclosure of contractual agreements that limit such access for investigators<br><br>Page 10; lines 353-358
Ancillary and post-trial care	30	Provisions, if any, for ancillary and post-trial care, and for compensation to those who suffer harm from trial participation<br><br>n/a
Dissemination policy	31a	Plans for investigators and sponsor to communicate trial results to participants, healthcare professionals, the public, and other relevant groups (eg, via publication, reporting in results databases, or other data sharing arrangements), including any publication restrictions<br><br>Page 12; lines 444-447
	31b	Authorship eligibility guidelines and any intended use of professional writers<br><br>Page 12; lines 446-447
	31c	Plans, if any, for granting public access to the full protocol, participant-level dataset, and statistical code<br><br>n/a
**Appendices**		
Informed consent materials	32	Model consent form and other related documentation given to participants and authorised surrogates<br><br>Appendices 2 and 3
Biological specimens	33	Plans for collection, laboratory evaluation, and storage of biological specimens for genetic or molecular analysis in the current trial and for future use in ancillary studies, if applicable<br><br>n/a

*It is strongly recommended that this checklist be read in conjunction with the SPIRIT 2013 Explanation & Elaboration for important clarification on the items. Amendments to the protocol should be tracked and dated. The SPIRIT checklist is copyrighted by the SPIRIT

For peer review only - http://bmjopen.bmj.com/site/about/guidelines.xhtml

Group under the Creative Commons "Attribution-NonCommercial-NoDerivs 3.0 Unported" license.

For peer review only

For peer review only - http://bmjopen.bmj.com/site/about/guidelines.xhtml

BMJ Open

Selumetinib in combination with dexamethasone for the treatment of relapsed/refractory RAS-pathway mutated paediatric and adult acute lymphoblastic leukaemia (SeluDex): study protocol for an international, parallel-group, dose-finding with expansion phase I/II trial

Journal:	*BMJ Open*
Manuscript ID	bmjopen-2021-059872.R1
Article Type:	Protocol
Date Submitted by the Author:	26-Jan-2022
Complete List of Authors:	Menne, Tobias; Northern Center for Cancer Care<br>Slade, Daniel; University of Birmingham Cancer Research UK Clinical Trials Unit<br>Savage, Joshua; University of Birmingham Cancer Research UK Clinical Trials Unit<br>Johnson, Sarah; University of Birmingham Cancer Research UK Clinical Trials Unit<br>Irving, Julie; Newcastle University<br>Kearns, Pamela; University of Birmingham Cancer Research UK Clinical Trials Unit<br>Plummer, Ruth; Northern Center for Cancer Care; Newcastle University<br>Shenton, Geoff; Great North Children's Hospital<br>Veal, Gareth J; Newcastle University<br>Vormoor, Britta; Princess Maxima Center for Pediatric Oncology<br>Vormoor, Josef; Princess Maxima Center for Pediatric Oncology; Newcastle University<br>Billingham, Lucinda; University of Birmingham Cancer Research UK Clinical Trials Unit
**Primary Subject Heading**:	Haematology (incl blood transfusion)
Secondary Subject Heading:	Paediatrics, Oncology
Keywords:	Clinical trials < THERAPEUTICS, Leukaemia < ONCOLOGY, Adult oncology < ONCOLOGY, Paediatric oncology < ONCOLOGY

SCHOLARONE™
Manuscripts

Selumetinib in combination with dexamethasone for the treatment of relapsed/refractory RAS-pathway mutated paediatric and adult acute lymphoblastic leukaemia (SeluDex): study protocol for an international, parallel-group, dose-finding with expansion phase I/II trial

Tobias Menne[1]*, Daniel Slade[2]*, Joshua Savage[2]*, Sarah Johnson[2], Julie Irving[3], Pamela R. Kearns[2], Ruth Plummer[1,3], Geoff Shenton[4], Gareth J. Veal[3], Britta Vormoor[5], Josef Vormoor[3,5,6#], Lucinda Billingham[1#†]

* These authors have contributed equally and should be considered joint first authors.

# These authors have contributed equally and should be considered joint senior authors.

† Corresponding author contact details:

Prof. Lucinda Billingham, Cancer Research UK Clinical Trials Unit (CRCTU), Institute of Cancer and Genomic Sciences, University of Birmingham, Edgbaston, Birmingham. B15 2TT. UK. Email: L.J.Billingham@bham.ac.uk

[1] Northern Centre for Cancer Care, Freeman Hospital, Newcastle upon Tyne Hospitals NHS Foundation Trust, Newcastle upon Tyne. NE7 7DN. UK

[2] Cancer Research UK Clinical Trials Unit (CRCTU), Institute of Cancer and Genomic Sciences, University of Birmingham, Edgbaston, Birmingham. B15 2TT. UK

[3] Wolfson Childhood Cancer Research Centre, Newcastle University Centre for Cancer, Newcastle University, Newcastle upon Tyne. NE1 7RU. UK

[4] Great North Children's Hospital, Royal Victoria Infirmary Hospital, Newcastle upon Tyne Hospitals NHS Foundation Trust, Newcastle upon Tyne. NE7 7DN. UK

[5] Princess Máxima Center for Paediatric Oncology, Heidelberglaan 25, 3584CS Utrecht. The Netherlands

[6] University Medical Center, Utrecht, Heidelberglaan 100, 3584CX Utrecht. The Netherlands

For peer review only

Abstract

Introduction

Event free survival rates at 15 years for paediatric patients with relapsed/refractory acute lymphoblastic leukaemia (ALL) are 30-50%, with 5-year survival for adult patients only 20%. Many patients with newly diagnosed and relapsed ALL harbour somatic RAS-signalling activation mutations. Induction therapy for ALL involves steroids, with preclinical data suggesting the combination of dexamethasone with the MEK1/2 inhibitor, selumetinib (ARRY-142886) has a synergistic anti-cancer effect.

Methods and analysis

The SeluDex trial is an international, parallel-group, dose-finding with expansion, phase I/II trial to assess the selumetinib/dexamethasone combination in adult and paediatric patients with relapsed/refractory, RAS pathway mutant ALL. The Cancer Research UK Clinical Trials Unit at University of Birmingham is the UK Coordinating Centre, with national hubs in Copenhagen, Denmark; Monza, Italy; Münster, Germany; Paris, France; and Utrecht, Netherlands. Patients with morphologically proven relapsed/refractory or progressive B-cell precursor or T-ALL, with demonstrated RAS pathway activating mutations are eligible. Adult patients are ≥18 years old, ECOG ≤2 and paediatric <18 years old, Lansky play scale ≥60% or Karnofsky score ≥60%. Phase I primary objective is the recommended phase II dose of selumetinib as defined by occurrence/non-occurrence of dose limiting toxicities using the continual reassessment method; phase II will evaluate preliminary anti-leukaemic activity of the combination, as defined by morphological response 28 days post treatment using a Bayesian approach. Target recruitment is between 26-42 patients (minimum 13 and maximum 21 per group), depending the number of phase I patients included in phase II.

Ethics and dissemination

Medical ethical committees of all the participating countries have approved the study protocol; initial (UK) ethics approval (17/YH/0123) was granted by Yorkshire & The Humber - Leeds West Research Ethics Committee. Participants are required to provide written informed consent/assent. Results will be disseminated through national and international presentations and peer-reviewed publications.

Trial registration number

ISRCTN: 92323261

Strengths and limitations of this study

- Novel combination of the MEK1/2 inhibitor, selumetinib, with dexamethasone
- Seamless phase I/II Bayesian trial design with a Continual Reassessment Method for dose escalation in phase I
- Parallel cohort trial design of adult and paediatric patients within one protocol
- Availability of CAR T-cell therapy since this trial started recruitment has competed for the same patient population, however, SeluDex offers a bridging treatment option for patients awaiting CAR T-cell therapy outside clinical studies or at relapse after CAR-T treatment

For peer review only

Introduction

Acute lymphoblastic leukaemia (ALL) is the most common childhood cancer, representing 26.8% of all childhood malignancies (1). While the overall cure rate for newly diagnosed paediatric ALL is approaching 90%, children with relapsed ALL (rALL) are still facing a poor outlook, with reported overall event-free survival rates at 15 years of 30-50% (2), and rALL remains a frequent cause of death.

In adults, the frequency of ALL is significantly lower, with an incidence of around one per 100,000 (3). ALL in adulthood has proven to be more challenging to treat compared to childhood ALL, with the disease being more resistant to chemotherapy, and patients have a reduced treatment tolerance especially in the elderly population. Five-year overall survival rate remains at ~20% among adult patients aged ≥60 years, even though improvement has been observed since 1980 (4).

Data demonstrate that mutations activating the RAS-signalling cascade (NRAS, KRAS, FLT3 and PTPN11 and cCBL) are highly prevalent at both diagnosis and relapse of paediatric B-cell precursor ALL (incidence 38% at relapse), and are associated with high-risk features such as early relapse, central nervous system (CNS) involvement, and chemoresistance (5). Neurofibromin (NF1) copy number variants, point mutations or intragenic deletions have been reported in approximately 4% of T-cell ALL, 14% of early T-cell precursor (ETP), ~30% of low hypodiploidy, 2% high risk of B-cell ALL and 75% of relapsed hypodiploid ALL (6-10). BRAF point mutation incidence is approximately 3% in T-cell ALL, 4% in ETP and 0.5% in high-risk B-cell ALL (7-9). IKZF2 and IKZF3 deletions which have recently been shown to activate the RAS-pathway have an incidence of approximately 18% in hypodiploid ALL (11). IL7Rα point mutations, deletions and in-frame alterations are found in approximately 9% of T-ALL (12, 13), and JAK1 point mutations are found in approximately 5% of T-ALL (14).

Selumetinib is a potent and selective allosteric MEK1/2 inhibitor that has been evaluated in phase II/III clinical trials for several cancers, including BRAF mutation-positive melanoma (15), metastatic uveal melanoma (16), pancreatic (17), colorectal (18), and KRAS mutation-positive NSCLC (19). Pre-clinical models suggest a synergistic effect when selumetinib and dexamethasone were combined in an orthotopic mouse model with three different RAS-pathway mutant ALL primagrafts and a mechanistic basis to the synergy was identified involving heightened levels of the proapoptotic Bim protein (20). However, the combination of selumetinib and dexamethasone has not been evaluated in a clinical trial setting.

The aim of the SeluDex trial is to determine the dose of selumetinib to use in combination with dexamethasone in adult and paediatric patient populations, and to assess the preliminary anti-leukaemic properties of the combination in each group of patients.

Methods and analysis

Study design

The SeluDex trial is a parallel-group, non-randomised, open-label, dose-finding with expansion, phase I/II clinical trial. As an early phase dose-finding and signal-seeking trial, a comparator arm was not included. Group A (adult patients) and Group P (paediatric patients) will both receive selumetinib in combination with a standard dose of dexamethasone (Figure 1). Patients will be recruited from public hospitals. The Cancer Research UK Clinical Trials Unit (CRCTU) at University of Birmingham is the UK Coordinating Centre, with national hubs in Copenhagen, Denmark; Monza, Italy; Münster, Germany; Paris, France; and Utrecht, Netherlands. The national coordinating and participating national centres are all part of the Innovative Therapies for Children with Cancer in Europe consortium (ITCC).

For peer review only

This trial aims to recruit between 26 and 42 patients (minimum of 13 and maximum of 21 in each group), depending on how many phase I patients are included also in phase II. The recruitment period is expected to be four years, with participants followed up for one month after a maximum of 6 treatment cycles. The World Health Organization (WHO) Trial Registration Data Set is attached in Supplementary Appendix 1.

Patient and public involvement

Patients and public were not involved in the design and conduct of this research but will be consulted in the reporting and dissemination of its outputs.

Patient selection

The two parallel patient groups in SeluDex, Group A (Adult) and P (Paediatric), have similar but discrete eligibility criteria. The main eligibility criteria are detailed in Tables 1 and 2, respectively. The screening process is the same for both groups. Eligibility for trial entry is dependent upon screening of a bone marrow aspirate to confirm morphological relapse and RAS-pathway mutation status. Peripheral blood may also be used in the case of a dry tap if white blood cell counts >50 x 10^9/L. Centralised genetic analysis in the UK at Northern Genetics Service's laboratories in Newcastle upon Tyne will identify the presence of a RAS-pathway activating mutation (key exons of NRAS, KRAS, FLT3, PTPN11 or cCBL mutation). A primary screen of the two most common mutations (NRAS and KRAS) will be performed first and if no mutations are found a secondary screen of the remaining mutations (FLT3, PTPN11 and cCBL) will be performed. Alternatively, locally obtained genetic results may be used to confer RAS-pathway mutation status if tested in a clinically compliant laboratory using a clinically validated test with material from the current relapse. Laboratories will be approved by the Trial Office to utilise local NRAS, KRAS, FLT3, PTPN11, cCBL, NF1, BRAF, IKZF2, IKZF3, IL7Rα or JAK1 testing.

Consent

Consent is requested by an investigator at two time points. A pre-trial genetic assessment consent allows for central/local analysis of a sample at screening to assess RAS mutation status, and the main trial consent, which is performed once mutation status is confirmed and a treatment slot is reserved. The main trial consent will be for either the phase I dose finding, of phase II dose expansion. Furthermore, age-specific consent/assent forms are available for patients over 16 years of age, and parents/legal guardians of paediatric patients (as appropriate according to age and national legislation). An example of the main consent form for adult patients is available in Supplementary Appendix 2. Age-specific patient information sheets are available for patients over 16 years of age, parents/legal guardians of paediatric patients, and teenage patients aged 13-15 years. As an example, the adult patients information sheet is available in Supplementary Appendix 3.

Interventions

The dosing schedule for the dose-finding phase of the trial is summarised below for each group and detailed in relevant tables. Treatments are given over a 28-day cycle and will be continued for six cycles unless one or more of the following is observed; intolerable toxicity, confirmed disease recurrence or progression, pregnancy, severe non-compliance to protocol, development of any trial-specific criteria for discontinuation (not reaching at least a partial response (PR) at Cycle 1 day 28), or investigator decision, for example, if the patient requires a prohibited concomitant medication. The trial schedule of events is included in Supplementary Appendix 4.

For peer review only

Group A (Table 3)

Dexamethasone: Steroids are a backbone of all induction blocks of ALL therapy with dexamethasone, the preferred steroid in the management of patients with ALL. Initially, dexamethasone was to be administered on a continuous dosing schedule with a weekly total dose of 42mg/m^2 for the first course. However, following an observed increase in the risk of infections during the initial stages of the trial, the trial dexamethasone dosing regimen was amended to allow intermittent treatment in blocks rather than continuously, and incorporating a dose reduction from 6mg/m^2/day in cycle 1 (D2-4 & D8-11) to 4mg/m^2/day during D15-18 and D22-25 of cycle 1. Further dexamethasone is given as a low dose in cycle 2 (D1-4 only: 4mg/m2/day) and then as a high dose again in subsequent cycles (C3-6, D1-5 only: 6mg/m^2/day).

Selumetinib: The dose of 75mg was chosen as the starting dose for Group A (Dose Level 0) with the dose only increased if pharmacokinetic (PK) data suggest the addition of dexamethasone decreases selumetinib serum concentrations significantly due to induction of the cytochrome P450 enzyme CYP3A4 (21).

Group P (Table 4)

Dexamethasone: As with adult dosing, trial dexamethasone dosing regimen was amended during the initial stages of the trial to be reduced after the first two weeks and to be given pulsed during cycle 1 due to the increase in infection risk.

Selumetinib: Results of two paediatric phase I trials using selumetinib only (22, 23) gave the maximum tolerated dose (MTD) as 2 x 25mg/m^2; thus in the SeluDex trial, 20mg/m^2 (80% of the single agent MTD of selumetinib) was chosen as the starting dose for safety reasons (Dose Level -1), specifically for those patients with CNS disease receiving concomitant intrathecal methotrexate.

Treatment compliance

Treatment compliance should be discussed with the patient/parent/legal guardian at the beginning of each cycle when study drug is dispensed. The research nurse should complete the relevant sections including recording the morning and afternoon doses and the dates they are to be taken on the diary. All patients/parents/legal guardians will be required to complete a diary with the daily time of administration of the study drugs, which must be returned to the clinic for checking at each visit. If a dose is missed, the reason must be noted in the diary by the patient/parent/legal guardian. Patients/parents/legal guardians should be advised to return any unused investigational medcicinal product in the original bottles, in addition to returning any empty bottles. Compliance should be reviewed at the end of each cycle by the site staff via review of the patient diary and discussion with the patient/parent/legal guardian that is to be documented in the medical notes.

Dose modifications

No dexamethasone dose reductions are permitted during cycle 1 and 2. If identifiable dexamethasone related intolerable toxicity is present during cycle 3 then a dose reduction to 4mg/m^2/day is allowed from cycle 4 onwards.

Dose modifications for the following selumetinib-related toxicities are allowed:

- Grade 3 neutropenia with infection and/or fever (GCSF is permitted)

For peer review only

- Grade 3 or 4 nausea, vomiting, or diarrhoea, if persistent despite optimal antiemetic and/or anti-diarrhoeal therapy
- Any Grade 3 or greater non-haematological toxicity
- Any other Grade 4 haematological toxicity

If one or more of these are observed the following action should be taken;

1st occurrence: Hold selumetinib until recovery to Grade <1 or baseline; restart at original dose level

2nd occurrence: Hold selumetinib until recovery to Grade <1 or baseline; keep same dose but reduce to once daily

3rd occurrence: Hold selumetinib until recovery to Grade <1 or baseline; use 60% of the original dose but go back to twice daily. For group P: 45mg reduce to 30 mg; 40 mg to 25 mg; 30 – 35 mg to 20 mg; 20 – 25 mg to 10 mg. For Group A: 75 mg reduce to 45 mg; 60 mg to 35 mg

4th occurrence: Discontinue selumetinib

Concomitant medication

Patients should avoid consuming large amounts of grapefruits, seville oranges, and any other products that may contain these fruits, e.g., grapefruit juice. In addition, patients should not take any vitamin E supplements. Restricted medications are listed in in Table 5.

Trial outcomes

Phase I

The primary objective for the phase I dose-finding component is to determine the recommended phase II dose (RP2D) of selumetinib in combination with dexamethasone, as defined by the occurrence/non-occurrence of dose limiting toxicities (DLTs) in the trial-defined assessment period. The trial will achieve this through the use of the continual reassessment method (CRM) (24). DLTs will be assessed from the first dose on cycle 1, day 1 up until cycle 1, day 28 during phase I only. A DLT is defined as any toxicity which is dose limiting, is not attributable to the disease or disease-related processes under investigation, and is considered at least possibly related to selumetinib or dexamethasone (see Dose Limiting Toxicity section).

Secondary outcomes for the phase I component are:

- The occurrence of adverse events (AEs) as measured by Common Terminology Criteria for Adverse Events (CTCAE) version 4 (25) and causality assessment
- Pharmacokinetic variables of selumetinib in combination with dexamethasone from the concentration time profile (area under the plasma concentration-time curve (AUC), C_{max}, T_{max}, $t_{1/2}$)
- Response to treatment assessed by complete remission rate at 28 days as measured by morphological and minimal residual disease (MRD) response in bone marrow (BM) and for patients with CNS involvement only clearance of cerebrospinal fluid (CSF) blasts at 28 days
- Difference in pharmacokinetics of selumetinib (ΔAUC) when selumetinib is administered as single agent and in combination with dexamethasone

For peer review only

Phase II

The primary objective for the phase II expansion component will be to assess preliminary anti-leukaemic activity of the selumetinib/dexamethasone drug combination as defined by morphological response (complete remission (CR), complete remission with incomplete platelet recovery (CRi), partial remission (PR), non-response (NR) and for patients with CNS involvement only, clearance of CSF blasts) 28 days post-treatment end. Response evaluation will be undertaken with bone marrow response at day 28, including MRD and CSF surveillance for CNS positive patients with standard cytology. A patient will be classified as having a response if they achieve CR or CRi at the day 28 assessment (with an acceptable window of day 15 to day 35).

Secondary outcomes for the phase II component are;

- The occurrence of AEs as measured by CTCAE version 4 and causality assessment
- The occurrence/non-occurrence of DLTs in the trial defined assessment period
- Pharmacokinetic variables of selumetinib in combination with dexamethasone from the concentration time profile (AUC, C_{max}, T_{max}, $t_{1/2}$)
- Difference in pharmacokinetics of selumetinib (ΔAUC) when selumetinib is administered as single agent and in combination with dexamethasone
- MRD response in bone marrow at 28 days

Exploratory outcomes of the trial for both phases of the trial include exploratory pharmacodynamic (PD) biomarker studies if clinical responses are observed. These will include levels of phosphorylated ERK by flow cytometry in leukaemic cells from patients at several time points (cycle 1 day 1, cycle 1 day 4 and end of treatment visit), as well as retrospective mRNA profiling, including the apoptotic initiator BIM (20, 26).

Dose limiting toxicities

DLTs will be assessed from the first dose on cycle 1 day 1 up until cycle 1 day 28 during Phase I. A haematological DLT is defined as bone marrow aplasia/hypoplasia, defined as overall marrow cellularity less than 25% (malignant infiltration or other causes are excluded), which does not recover by cycle 1 day 28. This includes an absolute neutrophil count of less than $0.5x10^9/L$ and a non-transfusion dependent platelet count of less than $25x10^9/L$ due to bone marrow aplasia/hypoplasia. In addition, Grade 4 febrile neutropenia, and any Grade 5 AE are also defined as a haematological DLT.

Non-haematological DLTs in Group A are defined as Left Ventricular Ejection Fraction <45% as measured by echocardiogram; and in Group P as >15% reduction in Shortening Fraction from baseline by echocardiogram. Results must be viewed by a consultant cardiologist to confirm that there has been a genuine deterioration in cardiac function. In addition, any Grade 5 AE, Grade 3 nausea or vomiting on or before C1D28 only which does not resolve to <Grade 2 within 48 hours (with or without supportive care), Grade 3 mucositis on or before C1D28 only which does not resolve to <Grade 2 within 48 hours (with or without supportive care), any other Grade 3 or 4 non-haematological toxicity, specifically excluding: Alopecia; dexamethasone related CTCAE Grade 3 toxicities (e.g., hyperglycaemia, muscle weakness, psychosis, avascular necrosis); tumour lysis syndrome; and increased alanine transaminase or aspartate aminotransferase.

Suspected DLTs will be reviewed and confirmed by the TSC during dose decision meetings at the end of each cohort during Phase I.

For peer review only - http://bmjopen.bmj.com/site/about/guidelines.xhtml

Statistical analysis plan

Both groups (A and P) will be analysed separately for both phases of the trial. If the response rates in the two groups are seen to be similar in the phase II component of the trial, then there may be some consideration to analysing response in the adult and paediatric groups collectively. The phase I evaluable population includes those patients who do not withdraw/die or discontinue treatment due to non-treatment related causes before the end of the DLT assessment period. A patient is evaluable regardless of the above if they experience a DLT. The phase II evaluable population includes all patients who received any treatment. There is no scope to replace unevaluable patients in the trial.

Phase I

The maximum number of patients to be recruited in phase I is 24; 12 into each group in parallel.

The trial will utilise the continual reassessment method (CRM) to find which doses to take forward to the phase II activity component of the trial for each group respectively. Each group will use a modified two-stage Bayesian CRM (27), recruiting patients in cohorts of two. The design incorporates rules to enable early stopping if the DLT rate at the lowest dose is unacceptably high. Patients are assigned to the starting dose level and follow the initial design (see Tables 3 and 4) until the first DLT is observed. Once the first DLT is observed the CRM takes over and determines the 'best' dose level that the next cohort should be assigned to, based on the model-based predicted probabilities of toxicity at each possible dose. The acceptable level of toxicity (probability of a DLT) has been chosen to be 17% for each group, that is, the trial will aim to find the highest dose that coincides with this acceptable level. This value was selected based on discussion with clinicians concerning the acceptable rate of DLTs based on the chosen definition.

The stopping rule for toxicity utilised in the modified CRM is based on the probability of the true toxicity rate at the lowest dose being >27%. If based on the observed data the probability of the true rate of toxicity being greater than this chosen value is >0.85 then the modified CRM will suggest that the trial should stop. The current MTD estimate is calculated after each cohort has been assessed for the full DLT assessment period and the next cohort of patients are then assigned to this MTD estimate. The final MTD estimate calculated after all 12 patients have been treated is the dose estimate to be considered to be taken forward to the phase II component.

This design works relatively well with computer simulations showing a probability for correctly selecting the right dose level of at least 0.6 when it is truly dose level 0 or 1, around 0.5 when it is truly dose level -1 and at least 0.6 for stopping early when all doses are truly too toxic.

Data will be reviewed at each dose decision stage by the independent Trial Safety Committee (TSC). The TSC will incorporate PK results into the decision process; the CRM will advise as to which doses are safe, with the PK additionally informing if there is a need for escalation/de-escalation.

Phase II

The phase II part of the trial will include patients from the phase I component that were dosed at the RP2D plus patients recruited into the expansion cohort. The number of phase I patients taken forward to the phase II assessment will be at least 4 in each group and the maximum number of patients to be recruited in the expansion cohort will be 9 in each group, giving a total of 13.

Based on data published by the TACL group (CR rate of 44% for second relapses and 27% for third relapses (28)) and seeing as we expect a mixture of 2nd, 3rd and higher relapses in paediatric patients and similar if

For peer review only - http://bmjopen.bmj.com/site/about/guidelines.xhtml

not worse relapse rates for adult patients, we estimate the overall response rate on standard treatment to be approximately 35%.

This phase will utilise a Bayesian approach to investigate the true response rate in each group. A Beta-Binomial conjugate analysis will be used to create a posterior probability distribution of the true response rate using the observed trial data combined with a Beta (1,1) minimally informative prior.The trial design is based on the decision criteria that if there is a high probability (>0.80) that the true response rate is >35% then this will indicate that the treatment is worthy of further research. With 13 patients, this design works reasonably well with a low probability of 0.08 of incorrectly concluding that the treatment is worthy when the true response rate is only 25% and a high probability of 0.82 of correctly concluding that the treatment is worthy when the true response rate is 55%.

If the response rates are similar in both groups, then information may be borrowed across Groups A and P to provide more accuracy in determining the true response rate for both groups collectively

There are no planned subgroup or interim analyses.

Pharmacokinetic and pharmacodynamic samples

Blood samples for PK and PD analysis will be obtained on days one, two and four of cycle 1, with blood samples for PK analysis collected again on day one of cycle 2, and blood samples for PD analysis obtained at the end of trial treatment.

For PK analysis plasma will be subjected to liquid chromatography-mass spectrometry (LC-MS) analysis to quantification concentrations of selumetinib, its major metabolite N-desmethyl selumetinib and dexamethasone. Two types of PD samples will be prepared from peripheral blood samples of patients who have a white cell count greater than $10x10^9$/L; samples will either be fixed on site in BD Lyse Fix solution or collected separately in Blood RNA Preservative Tubes. These PD samples will be transferred to the UK Biobank at the University of Birmingham for storage.

All samples will be collected in accordance with national regulations and requirements including standard operating procedures for logistics and infrastructure. Samples will be taken in appropriately licensed premises, stored and transported in accordance with the Human Tissue Authority guidelines and NHS trust policies.

Adverse events reporting and analysis

The collection and reporting of AEs as measured by CTCAE v4 will be in accordance with the Research Governance Framework for Health and Social Care and the requirements of the National Research Ethics Service (NRES). Definitions of different types of AEs are listed in online Supplementary Appendix 5; this includes abnormal laboratory findings which are considered clinically significant. The reporting period for AEs will be from the date of commencement of protocol defined treatment until 28 days after the administration of the last treatment. The investigator should assess the seriousness and causality (relatedness) of all AEs experienced by the patient (this should be documented in the source data) with reference to the protocol. In addition, DLTs will be assessed for the Phase I part of the trial only. All will be reported using the applicable electronic case report form (eCRF).

For peer review only

Data management

Case report forms (CRFs) can be entered online at https://www.cancertrials.bham.ac.uk/SeluDexLive. Authorised staff at sites will require an individual secure login username and password to access this online data entry system. Paper CRFs must be completed, signed/dated and returned to the SeluDex Trial Office by the investigator or an authorised member of the site research team. Data reported on each CRF should be consistent with the source data or the discrepancies should be explained. If information is not known, this must be clearly indicated on the CRF. All missing and ambiguous data will be queried. All sections are to be completed.

All trial records must be archived and securely retained for at least 25 years. No documents will be destroyed without prior approval from the sponsor, via the SeluDex Trial Office. On-site monitoring will be carried out as required following a risk assessment and as documented in the Quality Management Plan. Any monitoring activities will be reported to the central SeluDex Trial Office and any issues noted will be followed up to resolution. SeluDex will also be centrally monitored, which may trigger additional on-site monitoring. Further information regarding data management is provided in the study protocol.

The CRCTU will hold the final trial dataset and will be responsible for the controlled sharing of anonymised clinical trial data with the wider research community to maximise potential patient benefit while protecting the privacy and confidentiality of trial participants. Data anonymised in compliance with the Information Commissioners Office requirements, using a procedure based on guidelines from the Medical Research Council (MRC) Methodology Hubs, will be available for sharing with researchers outside of the trials team within 12 months of the primary publication.

Trial organisation structure

The University of Birmingham will act as sponsor to this international, multi-centre study: Support Group, Aston Webb Building, Room 119, Birmingham, B15 2TT. Email: researchgovernance@contacts.bham.ac.uk. The trial is being conducted under the auspices of the CRCTU, The University of Birmingham according to their local procedures. The Trial Management Group (TMG) will be responsible for the day-to-day running and management of the trial. Members of the TMG include the chief investigator, coinvestigators, biological coordinators, trial management team leader, senior trial coordinator, trial coordinator, lead trial statistician and trial statistician. The TMG will have regular meetings during recruitment. The TSC will consist of independent clinicians, Dr Bela Wrench and Dr Christina Halsey, as well as an independent statistician, Dr Graham Wheeler.

Data analyses will be supplied in confidence to the independent TSC, which will be asked to support dose decisions and advise on whether the accumulated data from the trial, together with the results from other relevant research, justify the continuing recruitment of further patients. The TSC will operate in accordance with a trial specific charter based on the template created by the Damocles Group. The TSC will meet at the end of every cohort in phase I and at least every 6 months in phase II, or more often if required e.g., an emergency meeting may also be convened if a safety issue is identified. The TSC will report directly to both the SeluDex TMG who will convey the findings of the TSC to the funders/sponsor as appropriate or when specifically requested by these parties.

Confidentiality

Confidential information collected during the trial will be stored in accordance with the General Data Protection Regulation (GDPR) 2018. As specified in the patient information sheet (PIS) and with the

For peer review only - http://bmjopen.bmj.com/site/about/guidelines.xhtml

patient's consent, patients will be identified using only their initials, date of birth and unique trial ID number. Authorised staff may have access to the records for quality assurance and audit purposes. The Trials Office maintains the confidentiality of all patients' data and will not disclose information by which patients may be identified to any third party other than those directly involved in the treatment of the patient and organisations for which the patient has given explicit consent for data transfer (e.g., laboratory staff).

Trial registration

ISRCTN: 92323261. Date applied: 22-Jan-2018; date assigned: 23-May-2018. Due to administrative delays, the date assigned occurred five days after the first patient was recruited (18-May-2018).

EudraCT: 2016-003904-29. Retrospective registration by UK competent authority (MHRA): 27-Mar-2019.

ClinicalTrials.Gov: NCT03705507. Retrospective registration: 15-Oct-2018.

ITCC-063 study

Trial status

Recruitment for the trial opened in April 2018 and the first patient was recruited on 18-May-2018; recruitment is expected to last until 31-Jan-2023. A list of open sites can be obtained from the SeluDex Trial Office (seludex@trials.bham.ac.uk).

Ethics and dissemination

The trial will be performed in accordance with the recommendations guiding physicians in biomedical research involving human subjects, adopted by the 18th World Medical Association General Assembly, Helsinki, Finland and stated in the respective participating countries laws governing human research, and Good Clinical Practice. The protocol was approved by the medical ethical committees of all the participating countries. UK ethics approval (17/YH/0123) was granted by Yorkshire & The Humber - Leeds West Research Ethics Committee on 12th July 2017. Initial UK Competent Authority approval was granted by Medicines and Healthcare Products Regulatory Agency (MHRA) on 5th May 2017, with subsequent protocol versions approved; current version in use is 9.0, 22nd March 2021. Participants are required to provide written informed consent/assent.A meeting will be held after the end of the study to allow discussion of the main results among the collaborators before publication. Results of the primary and secondary endpoints will be disseminated through national and international presentations and submitted for publication in peer-reviewed journals. Manuscripts will be prepared by the TMG and authorship determined by mutual agreement.

Discussion

Position of the trial in an era of CAR-T cell treatment

The SeluDex trial was initially proposed in 2013, before the advent of CAR-T cell and other CD19 and CD22 targeting immunotherapies. This trial was designed for patients who had relapsed after stem cell transplantation, with little or no other therapeutic options. The trial opened in the UK in Spring 2018. Since commercial CAR-T cell treatment (tisagenlecleucel) was approved by the FDA/EMA in 2018, eligible patients were preferentially offered CAR-T cell treatment over enrolment in this experimental study, and this has led to a significant drop in expected trial recruitment. After two years of experience with

For peer review only - http://bmjopen.bmj.com/site/about/guidelines.xhtml

tisagenlecleucel, relapse after cellular immunotherapy is emerging as a new therapeutic challenge (29). Relapses after CAR-T cell failure, especially CD19 negative relapses, and relapsed/refractory T cell ALL should be considered/screened for the SeluDex trial. For patients who have not had CD19 or CD22 targeting immunotherapy these should be prioritised, however, in exceptional circumstances the SeluDex trial (e.g., cycle 1) may also be considered as bridging therapy before CAR-T cell infusion.

Implemented urgent safety measures

Since the study opened to recruitment in 2018 there have been three urgent safety measures (USM) implemented in the protocol to date. The first USM was in December 2018 to reduce the dose of dexamethasone after two weeks during cycle 1 for Group A patients, after an adult patient experienced a fatal Suspected Unexpected Adverse Reaction (SUSAR) from rapid onset gram-negative sepsis; investigators suspected that the steroid administration had masked potential symptoms of infection and sepsis. The second USM was implemented in May 2019 to reduce the dose of dexamethasone after two weeks during cycle 1 for Group P patients, in line with the measure already implemented for adults, in addition to mandating fluoroquinolone prophylaxis during cycle 1 for all patients. This was due to a further fatal SUSAR of gram-negative sepsis in a paediatric patient. The third USM implemented in September 2019 changed the dexamethasone dose to pulsed during cycle 1 and mandated fluoroquinolone prophylaxis in cycle 2, in addition to cycle 1, and co-trimoxazole prophylaxis throughout trial treatment for all patients. This was due to a further fatal SUSAR in an adult patient from pneumonia considered related to trial treatment. Pulsed dosing of dexamethasone (four days on, three days off) is standard practice in the management of adult ALL patients and is better tolerated than continuous dexamethasone dosing during induction. Since these USMs were implemented, there have been no further events of concern and the TMG, TSC and regulatory authorities are confident that the potential patient benefit outweighs the risks of the intervention.

Trial strengths

This trial is, to the best of our knowledge, one of the first to not only use the novel combination of a MEK1/2 inhibitor, selumetinib, with dexamethasone but is also one of the first to span the whole age range of paediatric and adult patients. On the paediatric side, it is only limited because participants need to swallow the selumetinib capsules whole. As soon as a paediatric liquid formulation is available, all age limitations could be removed.

The seamless phase I/II Bayesian trial design with a CRM for dose escalation in phase greatly aids in the delivery of this trial, providing the flexibility to utilise cohorts of only two patients allowing for more frequent dose adjustment. Additionally, the CRM provides the means to estimate probabilities of DLT at all doses along with associated uncertainty intervals for those estimates, even those untested doses. The CRM in general identifies the MTD with greater accuracy and treats more patients at the MTD than traditional rule-based methods e.g. 3+3 (30).

Finally, the paediatric part of the SeluDex trial is an approved Innovative Therapies for Children with Cancer in Europe (ITCC) study with five National Co-ordinating Centres (NCC) outside the UK. The NCC of the Netherlands opened in Summer 2020, with Germany, France, Italy and Denmark planned to follow in 2021/22. This will help to improve the slow recruitment numbers resulting from the introduction of CAR-T cell therapy.

For peer review only - http://bmjopen.bmj.com/site/about/guidelines.xhtml

Summary

In summary, the SeluDex trial investigates the novel combination of the MEK1/2 inhibitor, selumetinib, with dexamethasone in a seamless phase I/II Bayesian trial design, in both adult and paediatric relapsed/refractory ALL patients. Although CAR T-cell therapy was approved after the trial started recruitment, the trial may serve as a bridging treatment option for patients awaiting CAR T-cell therapy and a treatment option for relapses failure once CAR-T cell treatment has failed.

Contributors

TM: Chief Investigator, study conception, study design, protocol development. DS: Senior Trial Biostatistician, study design, statistical plan development. JS: Trial Team Leader, study management, protocol development, coordination of IRAS, REC, MHRA, HRA and local R&D applications. SJ: Trial Coordinator, protocol development, coordination of IRAS, REC, MHRA, HRA and local R&D applications. PRK: Co-Investigator, study conception, study design, protocol development. JV: International Lead Investigator, study conception, study design, protocol development. BV: Co-Investigator, study conception, study design, protocol development. JI: Biological Coordinator, study conception, study design, protocol development. GV: Biological Coordinator, study conception, study design, protocol development. GS: Co-Investigator, protocol development. RP: Co-Investigator, study conception, study design, protocol development. LB: Chief Biostatistician, study conception, study design, protocol development, statistical plan development.

Funding

This work is supported by Cancer Research UK (C27943/A22304), CRUK trial number CRUKD/16/015 and (C27943/A23260), CRUK trial number CRUKD/16/016, and AstraZeneca through the CRUK's Combinations Alliance and Experimental Cancer Medicine Centre (ECMC). AstraZeneca provides Selumetinib to participating sites free-of-charge, and was consulted over the trial design but are not involved in the trial management group or safety committee. National Coordinating Centres were supported by a grant from ITCC "Imagine for Margo" fund and AstraZeneca. Staff at the CRCTU are also supported by a core funding grant from Cancer Research UK (C22436/A25354).

Role of funders and sponsor

Neither the sponsor or funders had any role in trial design, data collection, data analysis, data interpretation or writing of the report. The corresponding author had full access to all the data in the trial and had final responsibility for the decision to submit for publication.

Competing interests

JI has received research funding from F. Hoffmann-La Roche Ltd. All other authors declare no other relevant conflicts of interest.

Acknowledgements

The Investigators and Sponsor thank all the patients and their families who participated in this trial, as well as the NHS Trusts and staff and the members of the Trial Safety Committee, chaired by Dr Bela Wrench, who have supported this trial. SeluDex was supported by Experimental Cancer Medicine Centres (ECMC) funding and by the ECMC Network. This trial has been independently peer reviewed and has been adopted

For peer review only

by the National Institute for Health Research Clinical Research Network Portfolio. Special thanks to Dr Siân Lax for her contributions to this paper.

References

1. Kaatsch P. Epidemiology of childhood cancer. Cancer Treat Rev. 2010;36(4):277-85.

2. Einsiedel HG, von Stackelberg A, Hartmann R, Fengler R, Schrappe M, Janka-Schaub G, et al. Long-term outcome in children with relapsed ALL by risk-stratified salvage therapy: results of trial acute lymphoblastic leukemia-relapse study of the Berlin-Frankfurt-Münster Group 87. J Clin Oncol. 2005;23(31):7942-50.

3. Bassan R, Hoelzer D. Modern Therapy of Acute Lymphoblastic Leukemia. Journal of Clinical Oncology. 2011;29(5):532-43.

4. Geyer MB, Hsu M, Devlin SM, Tallman MS, Douer D, Park JH. Overall survival among older US adults with ALL remains low despite modest improvement since 1980: SEER analysis. Blood. 2017;129(13):1878-81.

5. Irving J, Matheson E, Minto L, Blair H, Case M, Halsey C, et al. Ras pathway mutations are prevalent in relapsed childhood acute lymphoblastic leukemia and confer sensitivity to MEK inhibition. Blood. 2014;124(23):3420-30.

6. Liu Y, Easton J, Shao Y, Maciaszek J, Wang Z, Wilkinson MR, et al. The genomic landscape of pediatric and young adult T-lineage acute lymphoblastic leukemia. Nature Genetics. 2017;49(8):1211-8.

7. Ryan SL, Matheson E, Grossmann V, Sinclair P, Bashton M, Schwab C, et al. The role of the RAS pathway in iAMP21-ALL. Leukemia. 2016;30(9):1824-31.

8. Zhang J, Ding L, Holmfeldt L, Wu G, Heatley SL, Payne-Turner D, et al. The genetic basis of early T-cell precursor acute lymphoblastic leukaemia. Nature. 2012;481(7380):157-63.

9. Zhang J, Mullighan CG, Harvey RC, Wu G, Chen X, Edmonson M, et al. Key pathways are frequently mutated in high-risk childhood acute lymphoblastic leukemia: a report from the Children's Oncology Group. Blood. 2011;118(11):3080-7.

10. Roberts KG, Li Y, Payne-Turner D, Harvey RC, Yang YL, Pei D, et al. Targetable kinase-activating lesions in Ph-like acute lymphoblastic leukemia. New England Journal of Medicine. 2014;371(11):1005-15.

11. Holmfeldt L, Wei L, Diaz-Flores E, Walsh M, Zhang J, Ding L, et al. The genomic landscape of hypodiploid acute lymphoblastic leukemia. Nat Genet. 2013;45(3):242-52.

12. Shochat C, Tal N, Bandapalli OR, Palmi C, Ganmore I, te Kronnie G, et al. Gain-of-function mutations in interleukin-7 receptor-α (IL7R) in childhood acute lymphoblastic leukemias. J Exp Med. 2011;208(5):901-8.

13. Zenatti PP, Ribeiro D, Li W, Zuurbier L, Silva MC, Paganin M, et al. Oncogenic IL7R gain-of-function mutations in childhood T-cell acute lymphoblastic leukemia. Nat Genet. 2011;43(10):932-9.

14. Jeong EG, Kim MS, Nam HK, Min CK, Lee S, Chung YJ, et al. Somatic mutations of JAK1 and JAK3 in acute leukemias and solid cancers. Clin Cancer Res. 2008;14(12):3716-21.

15. Goldinger SM, Zimmer L, Schulz C, Ugurel S, Hoeller C, Kaehler KC, et al. Upstream mitogen-activated protein kinase (MAPK) pathway inhibition: MEK inhibitor followed by a BRAF inhibitor in advanced melanoma patients. Eur J Cancer. 2014;50(2):406-10.

16. Carvajal RD, Piperno-Neumann S, Kapiteijn E, Chapman PB, Frank S, Joshua AM, et al. Selumetinib in Combination With Dacarbazine in Patients With Metastatic Uveal Melanoma: A Phase III, Multicenter, Randomized Trial (SUMIT). J Clin Oncol. 2018;36(12):1232-9.

17. Chung V, McDonough S, Philip PA, Cardin D, Wang-Gillam A, Hui L, et al. Effect of Selumetinib and MK-2206 vs Oxaliplatin and Fluorouracil in Patients With Metastatic Pancreatic Cancer After Prior Therapy: SWOG S1115 Study Randomized Clinical Trial. JAMA Oncol. 2017;3(4):516-22.

For peer review only

535 18. Krishnamurthy A, Dasari A, Noonan AM, Mehnert JM, Lockhart AC, Leong S, et al. Phase Ib Results of
536 the Rational Combination of Selumetinib and Cyclosporin A in Advanced Solid Tumors with an Expansion Cohort
537 in Metastatic Colorectal Cancer. Cancer Res. 2018;78(18):5398-407.

538 19. Jänne PA, Shaw AT, Pereira JR, Jeannin G, Vansteenkiste J, Barrios C, et al. Selumetinib plus docetaxel
539 for KRAS-mutant advanced non-small-cell lung cancer: a randomised, multicentre, placebo-controlled, phase 2
540 study. Lancet Oncol. 2013;14(1):38-47.

541 20. Matheson EC, Thomas H, Case M, Blair H, Jackson RK, Masic D, et al. Glucocorticoids and selumetinib
542 are highly synergistic in RAS pathway-mutated childhood acute lymphoblastic leukemia through upregulation of
543 BIM. Haematologica. 2019;104(9):1804-11.

544 21. McCune JS, Hawke RL, LeCluyse EL, Gillenwater HH, Hamilton G, Ritchie J, et al. In vivo and in vitro
545 induction of human cytochrome P4503A4 by dexamethasone. Clin Pharmacol Ther. 2000;68(4):356-66.

546 22. Banerjee A, Jakacki RI, Onar-Thomas A, Wu S, Nicolaides T, Young Poussaint T, et al. A phase I trial of
547 the MEK inhibitor selumetinib (AZD6244) in pediatric patients with recurrent or refractory low-grade glioma: a
548 Pediatric Brain Tumor Consortium (PBTC) study. Neuro Oncol. 2017;19(8):1135-44.

549 23. Dombi E, Baldwin A, Marcus LJ, Fisher MJ, Weiss B, Kim A, et al. Activity of Selumetinib in
550 Neurofibromatosis Type 1-Related Plexiform Neurofibromas. N Engl J Med. 2016;375(26):2550-60.

551 24. O'Quigley J, Pepe M, Fisher L. Continual reassessment method: a practical design for phase 1 clinical
552 trials in cancer. Biometrics. 1990;46(1):33-48.

553 25. Program CTE. Common Terminology Criteria for Adverse Events (CTCAE), Version 4.03. US Department
554 of Health and Human Services, NIH, NCI. 2010.

555 26. O'Connor L, Strasser A, O'Reilly LA, Hausmann G, Adams JM, Cory S, et al. Bim: a novel member of the
556 Bcl-2 family that promotes apoptosis. Embo j. 1998;17(2):384-95.

557 27. Goodman SN, Zahurak ML, Piantadosi S. Some practical improvements in the continual reassessment
558 method for phase I studies. Stat Med. 1995;14(11):1149-61.

559 28. Ko RH, Ji L, Barnette P, Bostrom B, Hutchinson R, Raetz E, et al. Outcome of patients treated for relapsed
560 or refractory acute lymphoblastic leukemia: a Therapeutic Advances in Childhood Leukemia Consortium study.
561 J Clin Oncol. 2010;28(4):648-54.

562 29. Nie Y, Lu W, Chen D, Tu H, Guo Z, Zhou X, et al. Mechanisms underlying CD19-positive ALL relapse after
563 anti-CD19 CAR T cell therapy and associated strategies. Biomarker Research. 2020;8(1):18.

564 30. Wheeler GM, Mander AP, Bedding A, Brock K, Cornelius V, Grieve AP, et al. How to design a dose-finding
565 study using the continual reassessment method. BMC Medical Research Methodology. 2019;19(1):18.

566

For peer review only

Figure Legend

Figure 1: SeluDex trial schema

SeluDex trial schema showing the patient pathway highlighting screening, trial entry, treatment, and follow-up.

For peer review only

Table 1: Main eligibility criteria for Group A in the SeluDex trial

Main Inclusion Criteria for Group A

Morphologically proven relapsed/refractory (M2 or M3 marrow; ≥1st relapse) or progressive B cell precursor or T-Acute Lymphoblastic Leukaemia (ALL) with demonstrated RAS pathway activating mutations (NRAS, KRAS, FLT3, PTPN11, cCBL, NF1, BRAF, IKZF2, IKZF3, IL7Rα or JAK1) identified during the trial screening process

B cell precursor patients must either:

- Have received CAR -T cell therapy, or
- Be awaiting CAR -T cell therapy, or
- Be considered ineligible for CAR -T cell therapy

≥18 years of age

Adequate renal function: Serum creatinine <1.5 x upper limit of normal (ULN)

Patient is able to swallow selumetinib capsules whole

Eastern Cooperative Oncology Group (ECOG) ≤2

Women of childbearing potential must have a negative pregnancy test

Patients who are women of childbearing potential and male patients with partners who are women of childbearing potential must agree to use appropriate contraception (see section 7.9.3 for definition) whilst on trial.

Written informed consent

Patients must have a body surface area (BSA) ≥ 0.55 m^2

Main Exclusion Criteria for Group A

ALL without presence of RAS-pathway activating mutations

Mature B-cell leukaemia and Philadelphia positive ALL

Prior exposure to MEK, RAS or RAF inhibitors

Any unresolved toxicity ≥ CTCAE Grade 2 from previous anti-cancer therapy, except for alopecia

Cardiac conditions as follows:

- Prior or current cardiomyopathy including but not limited to the following:
  - Known hypertrophic cardiomyopathy
  - Known arrhythmogenic right ventricular cardiomyopathy
- Even if full recovery has occurred, previous moderate or severe impairment of left ventricular systolic function (LVEF <45% on ECHO)
- Severe valvular heart disease
- Severe congenital heart disease
- Uncontrolled hypertension: BP ≥150/95 mmHg despite medical therapy
- Baseline (LVEF) below the lower limit of normal (LLN) or <55% measured by ECHO
- Acute coronary syndrome within 6 months prior to trial registration
- Uncontrolled Angina - Canadian Cardiovascular Society grade II-IV despite medical therapy
- Symptomatic heart failure New York Heart Association (NYHA) Class II-IV, prior or current cardiomyopathy, or severe valvular heart disease
- Atrial fibrillation with a ventricular rate >100 bpm on Electrocardiogram (ECG) at rest
- QTcF >450ms in male patients or ≥460ms in female patients, or other factors that increase the risk of QT prolongation

Ophthalmological conditions as follows:

- Current or past history of retinal pigment epithelial detachment (RPED)/central serous retinopathy (CSR) or retinal vein occlusion (RVO)
- Intraocular pressure (IOP) > 21 mmHg or uncontrolled glaucoma (irrespective of IOP)

Pregnant and breast-feeding females

For peer review only

Known severe hypersensitivity to selumetinib, dexamethasone or combination medications or any excipient of these medicinal products, or history of allergic reactions attributed to compounds of similar chemical or biologic composition to selumetinib

For peer review only

Table 2: Main eligibility criteria for Group P in the SeluDex trial

Main Inclusion Criteria for Group P

Morphologically proven relapsed/refractory (M2 or M3 marrow; ≥2nd relapse) or progressive B cell precursor or T-Acute Lymphoblastic Leukaemia (ALL) with demonstrated RAS pathway activating mutations (NRAS, KRAS, FLT3, PTPN11, cCBL, NF1, BRAF, IKZF2, IKZF3, IL7Rα or JAK1) identified during the trial screening process

B cell precursor patients must either:

- Have received CAR -T cell therapy, or
- Be awaiting CAR -T cell therapy, or

Be considered ineligible for CAR -T cell therapy

<18 years of age

Adequate renal function:

- ≤ 5 years: Serum creatinine <0.8 mg/dL or 70 µmol/L
- > 5 years but ≤ 10 years: Serum creatinine <1 mg/dL or 88 µmol/L
- > 10 years but ≤ 15 years: Serum creatinine <1.2 mg/dL or 106 µmol/L
- > 15 years: Serum creatinine <1.5 mg/dL or 132 µmol/L

Patient is able to swallow selumetinib capsules whole

Lansky play scale ≥60% or Karnofsky scale ≥60%

Women of childbearing potential must have a negative pregnancy test

Patients who are women of childbearing potential and male patients with partners who are women of childbearing potential must agree to use appropriate contraception (see section 7.9.3 for definition) whilst on trial.

Written informed consent

Patients must have a body surface area (BSA) ≥ 0.55 m^2

Main Exclusion Criteria for Group P

ALL without presence of RAS-pathway activating mutations

Mature B-cell leukaemia and Philadelphia positive ALL

Prior exposure to MEK, RAS or RAF inhibitors

Any unresolved toxicity ≥ CTCAE Grade 2 from previous anti-cancer therapy, except for alopecia

Cardiac conditions as follows:

- Prior or current cardiomyopathy including but not limited to the following:
  - Known hypertrophic cardiomyopathy
  - Known arrhythmogenic right ventricular cardiomyopathy
- Even if full recovery has occurred, previous moderate or severe impairment of left ventricular systolic function (SF <29% but excluding transient impairments due to e.g., anaemia/sepsis or results not thought to represent a true reflection of cardiac function)
- Severe valvular heart disease
- Severe congenital heart disease
- Uncontrolled hypertension: BP ≥95th percentile for age, height and gender
- Baseline SF <29%
- Atrial fibrillation with a ventricular rate >130 bpm on Electrocardiogram (ECG) at rest
- QTcF >450ms in patients <12 years or ≥460ms in patients ≥12 but <18 years

Ophthalmological conditions as follows:

- Current or past history of retinal pigment epithelial detachment (RPED)/central serous retinopathy (CSR) or retinal vein occlusion
- Intraocular pressure (IOP) > 21 mmHg or uncontrolled glaucoma (irrespective of IOP)

Pregnant and breast-feeding females

For peer review only

SeluDex

Known severe hypersensitivity to selumetinib, dexamethasone or combination medications or any excipient of these medicinal products, or history of allergic reactions attributed to compounds of similar chemical or biologic composition to selumetinib

For peer review only

For peer review only - http://bmjopen.bmj.com/site/about/guidelines.xhtml

Table 3: Group A (≥18 years) dosing schedule in phase I dose finding

Dose Level	Selumetinib (% of adult MTD)<br>Cycle 1 D1: single dose<br>Cycle 1 D4-D28: BD dosing<br>Subsequent cycles 2-6: D1-28: BD dosing		Dexamethasone<br>Cycle 1 D2-4 & D8-11: 6mg/m²/day, D15-18 & D22-25: 4mg/m²/day<br>Cycle 2 D1-4: 4mg/m²/day<br>Subsequent cycles 3-6, D1-5 only 6mg/m²/day	Initial Design<br>Applicable prior to DLTs
-1	80%	60mg BD PO	mg/m²***/day PO divided into 2 doses (as per local practice)	n/a
0	100%	75mg BD PO		Cohorts 1 and 2
+1*	113-120%**	85-90mg** BD PO		Cohorts 3, 4, 5 and 6

* **Important:** The selumetinib dose reverts from the +1 dose level to dose level 0 with the start of cycle 2 as patients will only be exposed to a short pulse of dexamethasone which is not expected to substantially affect selumetinib PK

** The proposed increase of dose might be changed based on PK results of dose level 0

*** Body surface area (BSA) for Group A to be calculated according to standard institutional practice

For peer review only

Table 4: Group P (<18 years) dosing schedule in phase I dose finding

**Dose Level**	**Selumetinib (% of adult MTD)**<br>Cycle 1 D1: single dose<br>Cycle 1 D4-D28: BD dosing<br>Subsequent cycles 2-6: D1-28: BD dosing		**Dexamethasone**<br>Cycle 1 D2-4 & D8-11: 6mg/m^2/day, D15-18 & D22-25: 4mg/m^2/day<br>Cycle 2 D1-4: 4mg/m^2/day<br>Subsequent cycles 3-6, D1-5 only 6mg/m^2/day	**Initial Design**<br>Applicable prior to DLTs
-1	80%	20mg/m^2*** BD PO	mg/m^2***/day PO divided into 2 doses (as per local practice)	Cohorts 1 and 2
0	100%	25mg/m^2*** BD PO		Cohorts 3 and 4
+1*	113-120%**	28-30mg**/m^2*** BD PO		Cohorts 5 and 6

* **Important:** The selumetinib dose reverts from the +1 dose level to dose level 0 with the start of cycle 2 as patients will only be exposed to a short pulse of dexamethasone which is not expected to substantially affect selumetinib PK

** The proposed increase of dose might be changed based on PK results of dose level 0

*** BSA for Group P to be calculated according to standard institutional practice

For peer review only

Table 5: Restricted medications within the SeluDex trial

• Acetazolamide<br>• Aminoglutethimide<br>• Antacids<br>• Anticholinesterases<br>• Anti-hypertensives<br>• Aprepitant<br>• Barbiturates<br>• Beta-naphthoflavone<br>• Carbamazepine<br>• Carbenoxolone<br>• Clarithromycin<br>• Coumarin anticoagulants<br>• Diltiazem<br>• Diuretics<br>• Efavirenz<br>• Ephedrine<br>• Erythromycin	• Fluconazole<br>• Fluvoxamine<br>• Glucocorticoids<br>• Hypoglycaemic agents, e.g. insulin<br>• Indinavir<br>• Itraconazole<br>• Ketoconazole<br>• Loop diuretics<br>• Methylcholanthrene<br>• Modafinil<br>• Nafcillin<br>• Nefazodone<br>• Nelfinavir<br>• Nevirapine<br>• Norethindrone<br>• NSAIDs<br>• Omeprazole	• Oral contraceptives<br>• Oxcarbazepine<br>• Phenobarbital<br>• Phenytoin<br>• Pioglitazone<br>• Prednisolone<br>• Primidone<br>• Rifabutin<br>• Rifampicin<br>• Ritonavir<br>• Salicylates<br>• Saquinavir<br>• Suboxone<br>• Telithromycin<br>• Thiazide diuretics<br>• Troglitazone<br>• Verapamil<br>• St John's Wort

For peer review only

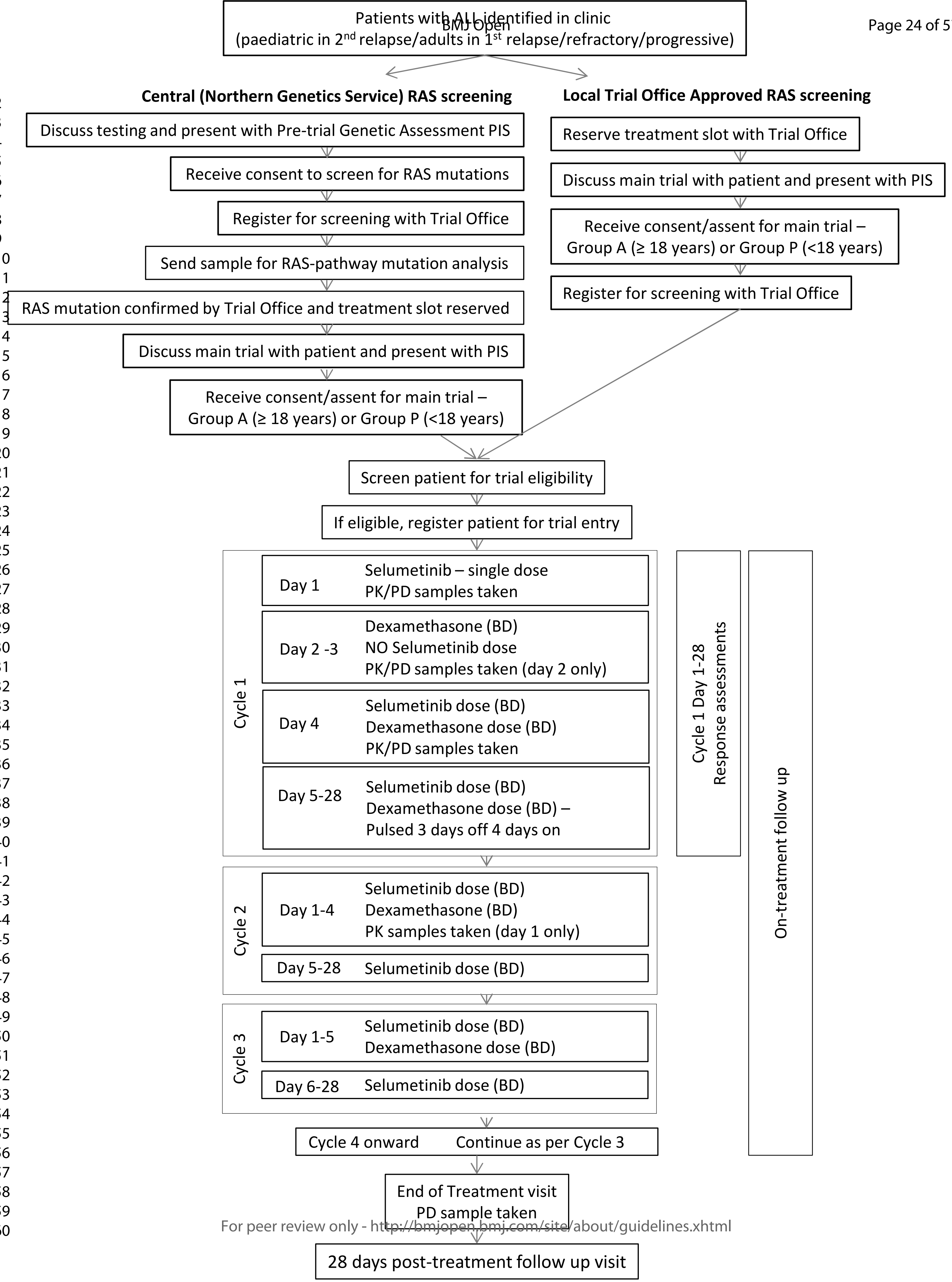
Patients with ALL identified in clinic
(paediatric in 2nd relapse/adults in 1st relapse/refractory/progressive)
Central (Northern Genetics Service) RAS screening
Discuss testing and present with Pre-trial Genetic Assessment PIS
Receive consent to screen for RAS mutations
Register for screening with Trial Office
Send sample for RAS-pathway mutation analysis
RAS mutation confirmed by Trial Office and treatment slot reserved
Discuss main trial with patient and present with PIS
Receive consent/assent for main trial – Group A (≥ 18 years) or Group P (<18 years)
Local Trial Office Approved RAS screening
Reserve treatment slot with Trial Office
Discuss main trial with patient and present with PIS
Receive consent/assent for main trial – Group A (≥ 18 years) or Group P (<18 years)
Register for screening with Trial Office
Screen patient for trial eligibility
If eligible, register patient for trial entry
Cycle 1
Day 1
Selumetinib – single dose
PK/PD samples taken
Day 2 -3
Dexamethasone (BD)
NO Selumetinib dose
PK/PD samples taken (day 2 only)
Day 4
Selumetinib dose (BD)
Dexamethasone dose (BD)
PK/PD samples taken
Day 5-28
Selumetinib dose (BD)
Dexamethasone dose (BD) –
Pulsed 3 days off 4 days on
Cycle 1 Day 1-28
Response assessments
On-treatment follow up
Cycle 2
Day 1-4
Selumetinib dose (BD)
Dexamethasone (BD)
PK samples taken (day 1 only)
Day 5-28
Selumetinib dose (BD)
Cycle 3
Day 1-5
Selumetinib dose (BD)
Dexamethasone dose (BD)
Day 6-28
Selumetinib dose (BD)
Cycle 4 onward
Continue as per Cycle 3
End of Treatment visit
PD sample taken
28 days post-treatment follow up visit

Supplementary Appendix 1

Data category	Information
Primary registry and trial identifying number	ISRCTN: 92323261
Date of registration in primary registry	Date applied: 22-Jan-2018<br>Date assigned: 23-May-2018. Due to administrative delays, the date assigned occurred five days after the first patient was recruited.
Secondary identifying numbers	EudraCT: 2016-003904-29ClinicalTrials.Gov: NCT03705507<br>ITCC-063 study
Source(s) of monetary or material support	Cancer Research UK<br>AstraZeneca<br>National Coordinating Centres were supported by a grant from ITCC "Imagine for Margo" fund and AstraZeneca
Primary sponsor	University of Birmingham
Secondary sponsor(s)	n/a
Contact for public queries	LB: L.J.Billingham@bham.ac.uk
Contact for scientific queries	TM: tobiasmenne@nhs.net
Public title	A trial looking at selumetinib and dexamethasone for acute lymphoblastic leukaemia (SeluDex)
Scientific title	SeluDex: an international trial of selumetinib in combination with dexamethasone for the treatment of acute lymphoblastic leukaemia

For peer review only

**Data category**	**Information**
Countries of recruitment	UK, Denmark, Italy, Germany, France, Netherlands
Health condition(s) or problem(s) studied	Relapsed/refractory acute lymphoblastic leukaemia
Intervention(s)	Dexamethasone and selumetinib
Key inclusion and exclusion criteria: **Adult Group**	Ages eligible for study: ≥18 years<br>Sexes eligible for study: both<br>Accepts healthy volunteers: no
	Inclusion criteria: adult patient (≥18 years) with proven ALL with demonstrated RAS pathway activating mutations, performance status ≤2
	Exclusion criteria: Prior exposure to MEK, RAS or RAF inhibitors, pregnancy or breastfeeding females, cardiac and/or ophthalmology conditions
Key inclusion and exclusion criteria: **Paediatric Group**	Ages eligible for study: <18 years<br>Sexes eligible for study: both<br>Accepts healthy volunteers: no
	Inclusion criteria: paediatric patient (<18 years) with proven ALL with demonstrated RAS pathway activating mutations, able to swallow selumetinib capsules whole, Lansky play scale ≥60% or Karnofsky scale ≥60%
	Exclusion criteria: Prior exposure to MEK, RAS or RAF inhibitors, pregnancy or breastfeeding females, cardiac and/or ophthalmology conditions
Study type	Interventional
	Allocation: non-randomised, open-label

For peer review only

SeluDex

Data category	Information
	Primary purpose: dose-finding and preliminary efficacy
	Phase I/II
Date of first enrolment	18-May-2018
Target sample size	Between 26 and 42 patients; minimum of 13 and maximum of 21 in each group,
Recruitment status	Open
Primary outcome(s)	Phase I: Occurrence/non-occurrence of dose limiting toxicities (time frame: between days 1 and 28 of cycle 1)<br>Phase II: Morphological response (time from: 28 days post-treatment end)
Key secondary outcome(s)	Occurrence of AEs as measured by CTCAE version 4 and causality assessment<br>Pharmacokinetic variables of selumetinib in combination with dexamethasone from the concentration time profile

For peer review only

__Please delete this line and print on hospital headed paper__

SeluDex: An international phase I/II expansion trial of the MEK inhibitor selumetinib in combination with dexamethasone for the treatment of relapsed/refractory RAS-pathway mutated paediatric and adult Acute Lymphoblastic Leukaemia

Informed Consent Form – Adult, Phase I

To be used only for participants over 16 years of age entering dose finding phase

EudraCT Reference: 2016-003904-29

Site: ______________________

Principal Investigator: ______________________

Trial Number (TNO): ☐☐☐ Screening Number (SNO): ☐☐ / ☐☐

Please INITIAL each box

1. I confirm that I have read and understand the ***Participant Information Sheet – Adult, Phase I*** (version……... dated………………………….) for the above trial. I have had the opportunity to consider the information, ask questions, and have had these answered satisfactorily ☐

2. I understand that my participation is voluntary and that I am free to withdraw at any time without giving any reason, without my medical care or legal rights being affected. I understand that if I withdraw from treatment my doctor may continue to provide the Trial Office with information that would routinely be collected about me and recorded in my medical notes. I am aware that I can also withdraw consent for this data transfer ☐

3. I give permission for my initials, date of birth, hospital number and NHS number to be given to the SeluDex Trial Office when I am registered to the trial as well as a copy of this consent form ☐

4. I understand that relevant sections of my medical notes and data collected during the trial may be looked at by individuals from the SeluDex Trial Office, regulatory authorities, Sponsor and/or NHS bodies, where it is relevant to my taking part in this research. I understand that this information will be held in a confidential manner. I give permission for ☐

Original to be kept in the Investigator Site File, 1 copy in hospital notes, 1 copy to the participant, 1 copy to the National Coordinating Centre

For peer review only - http://bmjopen.bmj.com/site/about/guidelines.xhtml

these individuals to have access to my records.

5. I understand that anonymised data from the trial may be provided to other third parties (e.g. pharmaceutical companies or other academic institutions) for research, safety monitoring or licensing purposes

6. I agree to my GP being informed of my participation in this trial

7. I understand that the SeluDex Trial Office, may access information held by national cancer registries and within national databases to keep in touch with me and to follow up on my health status

8. I give permission for collection of samples of my blood and bone marrow to be used in the SeluDex trial. I understand that samples will be sent to the University of Birmingham and other laboratories in the United Kingdom

9. I understand that DNA analysis may be performed on the samples taken for the trial.

10. I agree to take part in the above trial

The following is optional and will not affect entry into the trial, please initial in the relevant box:

No **Yes**

If in the event that an abnormality that might affect other family members is uncovered during genetic testing for the trial, I would like my doctor to be informed and to be referred to a genetic counsellor if appropriate.

Name of participant **Date** **Signature**

Name of person taking consent **Date** **Signature**

You must have signed the Site Signature & Delegation Log

This document was drafted using CRCTU-ICF-QCD-001, version 2.0

Original to be kept in the Investigator Site File, 1 copy in hospital notes, 1 copy to the participant, 1 copy to the National Coordinating Centre

For peer review only - http://bmjopen.bmj.com/site/about/guidelines.xhtml

Please delete this line and print on hospital headed paper

SeluDex

SeluDex: An international trial of selumetinib in combination with dexamethasone for the treatment of Acute Lymphoblastic Leukaemia.

Participant Information Sheet – Adult, Phase I

To be used only for patients over 16 years of age considering entering dose finding phase

---

This leaflet provides information about a clinical research trial and is intended to supplement your discussions about the trial with your doctor and nurses. Having read it, you may have further questions and these should be discussed with your consultant or one of the research team.

Dear Patient,

We would like to invite you to take part in a non-commercial, clinical trial being run by the University of Birmingham. Before you decide whether to take part in this trial, it is important for you to understand why the research is being done and what it would involve for you.

Please take time to read the following information carefully, and discuss it with your friends and relatives if you wish. Your doctor will go through the information sheet with you, and answer any questions you may have.

- Part 1 tells you the purpose of this trial, and what will happen to you if you take part
- Part 2 gives you more detailed information about the conduct of the trial

If there is anything that is not clear, or if you would like more information, please ask your doctor.

Take your time to decide whether or not you wish to take part. If you decide not to take part, this will not affect the quality of your care.

For peer review only - http://bmjopen.bmj.com/site/about/guidelines.xhtml

PART 1

What is the purpose of the trial?

The purpose of this trial is to test a new drug called selumetinib in combination with another drug called dexamethasone. The trial specifically includes those patients who have a relapse of their acute lymphoblastic leukaemia (ALL) or refractory ALL, and who have an identified change (mutation) in a particular gene in their cancer's DNA. We would like to see what effect combining these two drugs has on you and your leukaemia. This will include looking at how well this treatment works, finding out more information about how it affects the disease, and to see how safe the drugs are.

During Phase I, the part you have been invited to participate in, the trial will look at establishing what is the most suitable dose level of selumetinib in combination with dexamethasone. The purpose of this step of the research is to see what dose of selumetinib we can safely give to participants. We will get some preliminary information regarding the effectiveness of this combined treatment. We will also use results from your tests to see how the treatment affects the body.

After the Phase I part of the trial is complete, further patients who have not taken part in Phase I will be invited to participate in Phase II. The Phase II part of the trial will look at a dose level of selumetinib which has already been established in Phase I as being the most effective in combination with dexamethasone. The purpose of this step of the research is to develop further evidence about this recommended dose, and to see what effects the combination of these medications will have on participants' leukaemia. We will also continue to use results from tests to see how the treatment affects the body.

Why have I been invited to participate?

You have been invited to take part in the Phase I part of the trial because you have been diagnosed with relapsed ALL or with refractory ALL. If you have relapsed ALL and you are over 18 years of age this will be at least your first relapse whereas if you are 16-18 years of age this will be at least your second relapse. You will have previously consented to us undertaking a genetic test, or already had this testing done at hospital as part of your normal care, to assess the genetic status of your disease. This test has shown that your leukaemia carries a mutation in the RAS pathway, which means that you are a suitable candidate to take part in the SeluDex trial. Up to 42 participants will be recruited to the trial in total.

Do I have to take part?

No, you do not have to take part. It is up to you to decide whether or not to join the trial. Your doctor will describe the trial, and will go through this information sheet with you. They will also give you a copy of the information sheet to keep. If you decide to be part of the trial, you will be asked to sign a consent form to show you have agreed to participate. You are free to withdraw at any time, without giving a reason, and this will not affect the standard of care you receive thereafter.

What are the drugs being tested?

The investigational drugs being tested in this trial are called 'selumetinib' and 'dexamethasone'.

Selumetinib is manufactured and supplied by AstraZeneca, and it has not yet been licensed by the Medicines and Healthcare products Regulatory Agency (MHRA) and therefore only available as part of a clinical trial. As researchers learn more about the gene changes in cells

For peer review only - http://bmjopen.bmj.com/site/about/guidelines.xhtml

SeluDex

that cause cancer, they have been able to develop drugs that target these changes. Selumetinib is an example of such a drug. Cells normally grow in an orderly way. Chemical messages or signals tell them when to grow and when to stop. But if a gene becomes abnormal, a protein called MEK can produce a signal which makes cancer cells grow abnormally. Selumetinib is a MEK inhibitor, which means that it stops MEK proteins from sending signals to cancer cells for them to grow. This may stop or slow down the growth of cancer cells. As of 31 January 2020, approximately 4,335 patients have received selumetinib on its own, or in combination with another drug. You will take one dose of selumetinib on the first day of your first cycle and then from day 4 you will continue taking the drug throughout your treatment.

Dexamethasone is already used for the treatment of a number conditions including cancers and leukaemias, and belongs to a group of drugs known as glucocorticosteroids. These are important in the treatment of leukaemia due their ability to stimulate the death of cancer cells. Dexamethasone will be given as a tablet or oral suspension. You will not start taking dexamethasone until the second day of your first cycle, and will take this drug intermittently throughout treatment. You will take dexamethasone for 3 days during your first week of your first cycle, not take for 3 days and then take for 4 days until the beginning of cycle 2. After this, you will take dexamethasone just for the first five days of each cycle.

The research team at your hospital will provide you with more information on the routine for taking your medicine if you decide to take part in the trial.

If your leukaemia has also been detected in the central nervous system (CNS) (after a test called a lumbar puncture), additional treatment with intrathecal methotrexate (IT MTX) may be administered to help treat the leukaemia in the CNS.

It appears that it is more difficult for ALL participants receiving selumetinib and dexamethasone treatment in the SeluDex trial to ward off infections. Therefore participants have an increased risk of complications from this such as sepsis, which arises when the body's response to infection causes injury to its own tissues and organs requiring quick treatment. This could be due to the way selumetinib works in the body and that dexamethasone can hide symptoms of infection. Because of this you will receive a specific antibiotic (fluoroquinolone) during your first two cycles of treatment and another specific antibiotic (co-trimoxazole) throughout your treatment on the trial to help prevent this. Having preventative antibiotics is often part of your regular cancer care. Your doctor will also discuss with you that if you experience any signs of infection or sepsis (fever, fast heartbeat or breathing) you should contact your treating hospital straight away.

What will happen to me if I take part?

If you decide to take part, you will be asked to sign an Informed Consent Form to show that you have agreed to take part in the trial. After you have signed the consent form, you will undergo a period of screening tests to determine whether you are eligible and that it is safe for you to enter the trial.

Before you begin the trial:

You will need to have the following screening tests to ensure that it is appropriate and safe for you to take part. Some of these procedures are extra to your regular cancer care. If you have had some of them recently, they may not need to be repeated.

Routine tests and procedures

The research team will perform some routine checks. These will include:

- blood, urine tests
- an electrocardiogram (ECG), which will record the electrical activity of the heart
- a review of your medical history

For peer review only - http://bmjopen.bmj.com/site/about/guidelines.xhtml

- a physical examination
- assessment of your vital signs including blood pressure, temperature, heart rate
- review of your performance status, which is a measure of your general health and how your disease affects your daily routine
- you will also be asked questions about your health (e.g. date of diagnosis of your conditions, details of previous treatment or any other illnesses you may have) and what medicines you are taking at the moment
- a pregnancy test if you are female and of child-bearing potential

Extra tests and procedures

In addition to the tests above, an echocardiogram (ECHO) will be performed. This will record how your heart pumps blood and how the valves inside your heart look and work.

You will also have an eye examination. During the eye exam, the ophthalmologist will examine your eyes with a number of different instruments. They will look at the different structures of your eye to check for abnormalities. They may put drops in your eyes which will temporarily affect your vision, so you shouldn't drive yourself home. This test will be performed at screening only, but may be repeated whilst on treatment if deemed necessary by the trial doctor.

Fluid will be taken by lumbar puncture to test for leukaemia in the CNS which may or may not be routine. During this test treatment with intrathecal methotrexate (IT MTX) may be administered to help prevent or treat CNS relapse. This test will be performed for all participants at screening, and repeated at cycle 1 day 28 for those with CNS positive disease.

Please also refer to the summary table at the end of this information sheet. The screening period may last up to 3 weeks. If you are eligible to take part in the trial, you will be registered and given a unique trial number. If your doctor feels it is appropriate for your medical care, you may be given a course of one of three drugs (either dexamethasone, prednisolone or hydroxycarbamide) during the screening period. This treatment will end either seven days prior or up to one day prior to you registering and starting on the trial depending on the choice of pre-treatment.

During the trial:

You will be expected to do the following things during the trial:

- You will have to take selumetinib, twice-daily, continuously throughout the trial, and intermittent dexamethasone during the first and start of the second cycle and then for the first five days of each subsequent cycle
- You will have to have preventative antibiotic treatment
- Whilst taking the trial drugs you will be given a Patient Diary in which to record when you take your medication. Your research nurse will show you how to complete it. The diary will help you to remember if you have taken your dose and to record any side effects. If you accidently miss a dose, it is important that this is recorded in order for your doctor to see what medication you have taken. You will need to bring the diary to your clinic visits, and you will be issued with a new diary at the start of each new cycle of treatment. In addition, you must return the empty bottles and any unused tablets to your doctor when requested
- You will need to come to clinic for scheduled visits as indicated in the table at the end of this leaflet. In this trial, each 28-day period (4 weeks) is called a cycle of treatment
- You will be given a Patient ID Card which provides emergency contact details for the trial team. You should carry this with you at all times and present it to a doctor if you are admitted to a hospital

For peer review only - http://bmjopen.bmj.com/site/about/guidelines.xhtml

SeluDex

Routine tests and procedures

The research team will perform the following routine tests at some or all of the visits to see how the trial medication is affecting your body (please see the table at the end of this information sheet which shows when each assessment or activity is done): physical exam, pregnancy testing if you are female and of child-bearing potential, vital signs, performance status, ECG, and lab safety tests. You will also be asked questions about your health and what medicines you are taking at the moment.

Extra tests and procedures

Additional research blood samples and echocardiograms will be taken at various points during the trial treatment period. A full list of tests and procedures is included at the end of this information sheet.

How long will I be in the trial?

Most participants are expected to receive trial treatment for at least 1 or 2 cycles, and initially the number of cycles of trial treatment is six (approximately 6 months). Following this, participants who are considered to be receiving clinical benefit can remain on trial and have continued access to trial treatment whilst the trial is still ongoing after approval from AstraZeneca. If one drug is permanently stopped (for example due to serious side effects), your trial doctor may decide whether it is in your best interest to continue taking the other drug. If the drugs are not helping you, or you are having serious side effects, or you simply do not wish to continue with the trial treatment, you can stop the trial and you can discuss alternative treatment options with your doctor.

After you stop taking the trial drug

When you have finished taking the trial medication, you will need to come in to the clinic for an end of treatment visit. You will undergo the same routine and extra tests and procedures as performed during the trial (outlined in the table at the end of this information sheet). These are done to see how the trial affected your health. Depending on how well you responded to treatment and if your hospital has the appropriate facilities you may be asked to give a sample of your bone marrow or blood using a hospital consent form to store for future research. This would enable researchers to obtain the samples in the future to look if the RAS pathway activating mutation that was identified in your leukaemia at the start of the trial is still there to learn more about how effective the treatment is.

In addition, you need to come in for a follow-up visit 28 days after the last trial medication was taken. Any side effects you may still have, and other medication you may be taking will be reviewed.

After the follow up visit at 28 days, you will be regularly reviewed as part of your ongoing care by your usual doctor.

Payments

You will not receive any money for taking part in this trial.

What will I have to do?

You will be required to:

- Attend the hospital as requested for assessments on your progress. You will need to have regular checks. Any side effects of this medicine may continue after you finish your treatment so it is important to report side effects promptly

For peer review only - http://bmjopen.bmj.com/site/about/guidelines.xhtml

- Take selumetinib capsules, commencing with one dose on Day 1 and twice daily from day 4 after pre-treatment assessments are completed. Your research nurse will give you guidance on this. Capsules should be taken whole, on an empty stomach (no food or drink other than water for 2 hours prior to dosing and 1 hour after dosing) and with a glassful of water
- Selumetinib doses should be taken approximately 12 hours apart at the same time points each day
- Take dexamethasone on day 2-4, 8-11, 15-18 and 22-25 of your first treatment cycle, day 1-4 of your second treatment cycle, then during the first five days of each subsequent cycle
- Have preventative antibiotic treatment
- Report any symptoms to the trial doctor when you attend for clinic appointments. In between clinic appointments, call the study team if you experience any severe side effects
- If you are on regular medication or herbal supplements, please make sure your oncology doctor and research nurse know about them before you start your treatment so that they can be checked for compatibility with your trial treatment
- After starting your trial treatment, if any new medication is required, it is important that you tell the doctor prescribing the new medication that you are in a clinical trial and are taking selumetinib and dexamethasone. A concomitant medication and restrictions card is provided to carry with you. It is also important that you tell the oncology doctor if you have been asked to start taking any new medications
- Do not have any vaccinations without your Doctor's approval
- If you are going to have surgery or any other procedures, tell your doctor or dentist you are taking part in this trial and inform them of the trial medication
- Bring all unused or remaining trial medication with you to each clinic visit
- Complete a diary to record when you took your trial medication, and bring the diary with you to your clinic appointments. The diary will contain more specific instructions about how to take your medication. A new diary will be provided to you at the beginning of each cycle of treatment
- Stop wearing contact lenses if you experience any mild to moderate eye symptoms. Also, you must not use any eye drops for the treatment of eye symptoms (unless agreed by your doctor). You are advised to talk to your doctor if you have any concerns
- Refrain from:
    - Eating or drinking large amounts of grapefruit/grapefruit juice (no more than a small glass of grapefruit juice (120ml) or half a grapefruit daily)
    - Taking any vitamin or herbal supplements
- Avoid excessive sun exposure, and use adequate sunscreen protection if sun exposure is anticipated
- You must not take Vitamin E vitamins or supplements
- It is recommended that you apply alcohol-free moisturiser to your skin before bedtime.

What are the possible side effects of the treatment?

You may have side effects while on the trial. All treatments can have side effects. Everyone taking part in the trial will be monitored carefully for any side effects, however doctors don't know all the side effects that may happen. Side effects may be mild or very serious. Your health care team may give you medicines to help lessen side effects or in some cases treatment may be delayed.

Participants in this trial will be at risk of known and unknown side effects of selumetinib and dexamethasone, as well as potential unknown risks due to the combination of the 2 drugs.

For peer review only - http://bmjopen.bmj.com/site/about/guidelines.xhtml

Participants with central nervous system (CNS) positive disease may be treated with intrathecal methotrexate (IT MTX) so may also be at risk of methotrexate induced neurotoxicity.

In the event of severe toxicity, and at the advice of your doctor, trial treatment may be interrupted temporarily until side effects have eased. Following an interruption, trial treatment could be restarted at a lower dose. If the side effects have not sufficiently reduced, trial treatment will be permanently stopped.

Selumetinib

Selumetinib is a new drug. More than 3,080 people have had the drug so far, and the drug has been very well tolerated. As it is a new drug, there may be side effects from taking selumetinib that doctors don't know about yet. The side effects already associated with selumetinib are listed below.

Common side effects of selumetinib:

More than 10 in every 100 people (10%) have one or more of these:

- Skin rash, which may be itchy in about 7 out of 10 people (70%). The majority of events are mild, but in some cases (around 10%) this can be more serious and require treatment
- Diarrhoea in about 5 out of 10 people (50%). It may occur within days of starting selumetinib treatment, and participants are advised to call the trial team as soon as possible so that treatment can be started promptly to help manage these side effects
- Feeling sick in about 5 out of 10 people (50%), or being sick in about 3 out of 10 people (30%). Participants are advised to call the trial team as soon as possible so that treatment can be started promptly to help manage these side effects
- Feeling tired or weak in around 4 out of 10 people (40%)
- Swelling of the hands or feet occurs in around 3 out of 10 people (30%)
- Shortness of breath in around 2 out of 10 people (20%). If you experience difficulty breathing or shortness of breath, call the trial team immediately
- Swelling of the face or extremities is commonly reported in patients receiving selumetinib
- Patients may also experience soreness or inflammation of the mouth, which may begin within days of starting treatment and usually occurs within the first month of treatment

Less common side effects of selumetinib:

Between 1 and 10 in every 100 people (1%-10%) have one or more of these:

- Swelling of the face, hands or feet
- Mouth sores, dry mouth
- High temperature
- Dry skin and soreness or infection in the skin around fingernails or toe nails. Sun exposure should be avoided when receiving selumetinib treatment
- Blurred vision – this has been mild or moderate in all reported previous cases. There is around a 5% chance that this may affect your daily living, such as using the telephone
- An increase in blood pressure
- Changes in the way your liver works
- Small decrease in cardiac (heart) function – this is usually mild
- Changes in your lungs – this will be carefully monitored by your doctor
- A lower than normal amount of blood cells that carry oxygen (anaemia)

Early information from clinical studies suggests that people of Asian origin may experience higher blood levels of selumetinib than non-Asian subjects. Higher levels of selumetinib in the blood may cause more side effects. Your trial doctor will discuss this information with you if it might affect your participation in the trial.

For peer review only - http://bmjopen.bmj.com/site/about/guidelines.xhtml

SeluDex

Dexamethasone

This drug has a known side effect profile as listed below:

- Increased appetite/weight gain
- Fluid retention
- An increase in blood pressure
- Excessive glucose in the bloodstream (hyperglycaemia)
- Steroid-induced diabetes mellitus
- Increased sensitivity to infection
- Disturbance of sleep patterns
- Low mood/irritability
- Acne type skin rashes/stretch marks
- Inflammation of the pancreas
- Death of bone tissue due to a lack of blood supply (avascular necrosis)

Intrathecal Methotrexate (IT MTX)

IT MTX may be given to trial participants in whom the leukaemia was found in the fluid taken by lumbar puncture, these are called "CNS positive participants". This drug has the potential for rare cases of neurotoxicity. Patients can experience acute toxicity (headache, nausea/vomiting, stiff neck) or delayed side effects (seizures, altered levels of consciousness and encephalopathy). It has been used for several decades in combination with intensive chemotherapy for the treatment of paediatric and adult ALL order to prevent CNS relapses. All participants will be regularly assessed for the occurrence of adverse effects, and CNS positive participants will also have regular full neurological examinations.

What are the possible benefits of taking part?

We cannot predict whether you will benefit directly from taking part in this trial. All participants in the trial will be closely monitored and supported by the research team. It is possible that your disease may respond to the trial treatment. We cannot guarantee that this treatment will help you, but the information we get from this trial will help us to improve the treatment of people with ALL in the future. You will not be paid for the use of your samples, even if the research does lead to the development of new medical products. Such developments take time and are unlikely to be critically dependent upon samples from any particular participant.

What are the possible disadvantages and risks of taking part?

It is possible that this combination of treatment will not work and you will still experience side effects.

Blood samples

You may experience a little discomfort when a needle is put into a vein or a port-a-cath to take blood samples. There may be slight pain, a small amount of bleeding, discolouration, or bruising at the site where the needle is inserted (but this will clear after a week or two). There is also a risk of infection or phlebitis (abnormal blood clot), but these rarely happen. Blood samples will be grouped together and taken with routine samples as much as possible to minimise the number of times a needle is inserted into your vein.

Bone marrow samples

A bone marrow test is taken during your screening visit and again after 4 weeks of treatment to see how well you have responded to the treatment. This is used to look for the amount of leukaemia that you have left and is done by microscope examination and in addition a minimal residual disease (MRD) test is performed on the screening sample and on future samples for participants who have responded well. The site of the bone marrow examination might be

For peer review only - http://bmjopen.bmj.com/site/about/guidelines.xhtml

painful for a few days afterwards. A bone marrow examination may be performed under a general anaesthetic.

Lumbar puncture

A lumbar puncture is performed during screening and might get repeated in case the leukaemia is found in the fluid taken during the initial lumbar puncture. In this case your physician might want to treat you with IT MTX (see paragraph on side effects of treatment). A lumbar puncture may be performed under a general anaesthetic together with a bone marrow examination. Side effects of the procedure include headaches, local discomfort, bruising, bleeding and a low risk of infection.

Possible harm to the unborn child and pregnancy

Selumetinib may have a harmful effect on a baby developing in the womb when administered to a pregnant woman. Reproductive toxicology data indicate that selumetinib has adverse effects on embryo foetal development and survival, at dose levels that do not induce maternal toxicity in animal models. It is therefore essential that both men and women taking the trial treatment must agree to use a reliable form of contraception during the trial, e.g. oral contraceptive and condom, intra-uterine device (IUD) such as Mirena Coil and condom, diaphragm with spermicide and condom. Total abstinence is only an acceptable method of contraception when it is your usual and preferred lifestyle choice. You should continue the use of adequate contraception for at least 12 weeks after the treatment has finished.

Information for women

You should not take part in this trial if you are pregnant, breastfeeding or may become pregnant during the trial period. If you are female and of childbearing potential, you will have a pregnancy test during screening and then monthly whilst on trial. This may be repeated if pregnancy is suspected whilst you are on the trial. You must agree to use two reliable forms of contraception (as detailed above) for two weeks prior to entering the trial, during the trial, and for at least 12 weeks after the treatment has finished.

If you do become pregnant during the course of the trial, please tell your trial doctor immediately. The pregnancy will need to be monitored and information about the outcome of your pregnancy will be collected from your medical notes and those of your baby.

Information for men

If you have a partner who is pregnant or who could become pregnant you must agree to use two reliable forms of contraception (as detailed above) for two weeks prior to entering the trial, during the trial, and for at least 12 weeks after the treatment has finished.

If your partner becomes pregnant whilst you are on the trial treatment, please tell your doctor immediately. We would also like to collect information about the outcome of the pregnancy. If your partner becomes pregnant during the trial, we will ask your partner to consent to this monitoring, if she is happy to do so.

What are the alternatives for treatment?

It is important that you discuss any possible treatment alternatives with your doctors before deciding whether to enter this trial. If you decide not to participate in this trial, you will receive standard or palliative care, or may enter another early phase trial (if available).

What happens when the research trial stops?

The trial treatment will not be available after the end of the trial. You will receive trial treatment should it be of benefit only for the duration of the trial. Initially this will be for 6 cycles (approximately 6 months). After this if treatment is considered to be of benefit to you then you may remain on treatment by continuing on the trial whilst it is ongoing following approval from

For peer review only - http://bmjopen.bmj.com/site/about/guidelines.xhtml

SeluDex

AstraZeneca. The schedule of assessments for treatment continuation will follow cycles 3-6 (see table at the end of this document). At the end of the research trial, or if you withdraw from the trial before it ends, your trial doctor will assess your symptoms and discuss your options and prescribe further alternative treatment if appropriate. If the academic body sponsoring the research trial decides to stop the trial before it has finished, your trial doctor will explain the reasons why and arrange appropriate care for you.

What if there is a problem?

Any complaint about the way you have been treated during the trial or any possible harm you might suffer will be addressed. The detailed information on this is given in part 2 of this leaflet.

Will my taking part in the trial be kept confidential?

Yes. We will follow ethical and legal practice, and all information about you will be handled in confidence. The details are included in Part 2 of this leaflet.

This completes Part 1 of the Participant Information Sheet

If the information in Part 1 has interested you, and you are considering participation, please read the additional information in Part 2 before making any decision

For peer review only

For peer review only - http://bmjopen.bmj.com/site/about/guidelines.xhtml

SeluDex

PART 2

What if relevant new information becomes available?

Sometimes during the course of a research trial, new information becomes available about the disease or drug that is being studied. If this happens, your doctor will tell you about it and discuss with you whether you want to continue on the trial. If you decide to withdraw, your doctor will make arrangements for your care to continue in a different way. If you decide to continue in the trial you may be asked to sign an updated consent form. If the trial is stopped for any other reason, we will tell you and arrange your continuing care.

What will happen if I don't want to carry on with the trial?

You are free to withdraw from the trial at any time. You do not have to give a reason and your future treatment will not be affected. Your doctor will discuss your treatment options with you and will offer you the most suitable treatment available. However, if you choose to withdraw entirely from the trial, we would still like to use the information we have collected about you up until withdrawal. If you just want to stop trial treatment but are happy to be seen in accordance with the schedule specified in this information leaflet please let your local trial team know. Alternatively we would like to ask your permission for your hospital to continue to send information about your progress to the SeluDex Trial Office. This is information that is routinely taken and you will not need to do anything extra. Any stored blood or tissue samples collected specifically for this trial up until the point of withdrawal will be retained and analysed. With your permission, the SeluDex Trial Office would also like to obtain information about your progress from the cancer registries and national databases (for example NHS Digital).

What if there is a problem?

You will be closely monitored both during and after treatment, and any side effects you experience will be treated.

Complaints: If you have a concern about any aspect of this trial, you should ask to speak with the trial doctor who will do their best to answer your questions (see contact number at the end of this form). If you remain unhappy and wish to complain formally, you can do this through the NHS Complaints Procedure. Details can be obtained from the hospital.

Harm: Every care will be taken in the course of this clinical trial. However, in the unlikely event that you are harmed and this is due to someone's negligence then you may have grounds for legal action for compensation against the trial sponsor (University of Birmingham) or the NHS Trust treating you, but you may have to pay your legal costs. NHS Trust and Non-Trust Hospitals have a duty of care to patients treated, whether or not the patient is taking part in a clinical trial and the normal NHS complaints mechanisms will still be available to you. The sponsor of the trial does not hold insurance against claims for compensation for injury caused by participation in this trial and they cannot offer any indemnity.

Participants may also be able to claim compensation for injury caused by participation in this clinical trial without the need to prove negligence on the part of the sponsor or another party. You should discuss this possibility with your trial doctor in the same way as above.

Regardless of this, if you wish to complain, or have any concerns about any aspect of the way you have been approached or treated by members of staff or about any side effects (adverse events) you may have experienced due to your participation in the clinical trial, the normal NHS complaints mechanisms are available to you. Please ask your trial doctor if you would like more information on this. Details can also be obtained from the Department of Health website: http://www.dh.gov.uk.

For peer review only - http://bmjopen.bmj.com/site/about/guidelines.xhtml

What will happen to the samples that I give?

Routine safety blood and urine samples will be analysed at your hospital laboratory throughout the course of the trial to see how the trial medication is affecting your body (pharmacodynamic analysis) and how your body processes the trial medication (pharmacokinetic analysis).

Throughout the course of the trial, additional samples will be taken for the purpose of the research. These samples will be labelled and identified with a unique sample ID number, your trial number and sometimes date of birth and initials. Samples will be sent to three different locations:

- Samples collected for pharmacokinetic analysis will be sent to Newcastle University
- Samples for pharmacodynamic analysis will be initially stored at the biobank at the University of Birmingham and and may be subsequently analysed at Newcastle University and at AstraZeneca
- Minimal Residual Disease (MRD) samples, if not tested routinely at your hospital, will be sent to University College, London for analysis.

Any samples remaining at the end of the trial may be stored and used for future ethically approved research which may involve genetic analysis, animal or other laboratory work at commercial or private institutions, and which may take place in the UK or overseas. If your samples are used, they will be anonymised.

Will any genetic tests be done?

Prior to being approached by your doctor to consider taking part in this trial, you will have consented to give a sample to assess whether your disease is positive for a RAS pathway activating mutation in its genetic make up.

What will happen to the results of the research trial?

At the end of the trial, the information collected will be analysed and published in recognised medical journals. You will not be identified in any report or publication. The results will help doctors to decide how to treat relapsed ALL in the future. Participants taking part in this trial can find out about the results from their trial doctor once the results have been published. The results will also be available on the Cancer Research UK website and the CRCTU website (http://www.birmingham.ac.uk/crctu). We expect the first results to be available in approximately 3 years.

Who is organising and funding the research?

The trial is an investigator-initiated and investigator-led trial, and is being carried out by a network of doctors across the UK and internationally. The trial is sponsored by the University of Birmingham and is being coordinated by the Cancer Research UK Clinical Trials Unit (CRCTU). Financial support for the trial is provided by the charity Cancer Research UK and from a pharmaceutical company called AstraZeneca. Your doctor will not receive any payments for talking with you about, or recruiting you into, this research trial.

Who has reviewed the trial?

All research in the NHS is looked at by independent group of people called a Research Ethics Committee to protect your safety, rights, wellbeing and dignity. This trial has been reviewed and given favourable opinion by a Research Ethics Committee, and by the local Research and Development department at your hospital's Trust. Whilst the trial is ongoing, the results will be reviewed by a Trial Safety Committee (TSC) to ensure that it is appropriate to continue with the trial.

For peer review only - http://bmjopen.bmj.com/site/about/guidelines.xhtml

Will my taking part in this trial be kept confidential?

All your details and information collected about you for this research trial will be subject to the General Data Protection Regulation (GDPR) and the Data Protection Act 2018 and will be kept totally confidential. Your initials, date of birth, ethnicity, hospital number and NHS number (or Community Health Index (CHI) in Scotland) will be supplied to the Cancer Research UK Clinical Trials Unit when you are entered in to the trial along with a copy of your consent form. In routine communication, your hospital the Cancer Research UK Clinical Trials Unit will refer to you only by a unique trial number allocated to you, and/or your date of birth and initials. All information about you will be securely stored, in both electronic format and paper form by the researchers, and will only be accessible by authorised personnel.

Occasionally, we may need to check your medical records to make sure that the information provided about you is accurate. This will be done either by clinical staff or by designated trial personnel. It may also be necessary to allow authorised personnel from government regulatory agencies, the sponsor of the trial (the group legally responsible for the conduct of the trial) and/or NHS bodies to have access to information about you. This is for your protection, and is to ensure that the research trial is being conducted to the highest possible standards.

If you agree to take part in this trial, then, in addition to the authorised personnel who review the main trial information, medical information about you may be passed on to researchers for future medical research. The information that they will be given for their work relates to your medical condition and treatment only and will not be directly linked to your identity. Researchers will not be able to contact you directly about their research in the future and you will not be identified in any reports or publications resulting from the study.

*At <insert hospital name> this trial will be managed by the <insert name> Clinical Trials Unit at <insert location> where the clinical team looking after you reside. Therefore it will be necessary for identifiable information to be transferred to and held by the Clinical Trials Unit. This includes a copy of the consent form which you will sign if you agree to take part in the trial. This information will remain confidential at all times.

*Delete as appropriate

The University of Birmingham is the sponsor for this study based in the United Kingdom. We will be using information from you and/or your medical records in order to undertake this study and will act as the data controller for this study. This means that we are responsible for looking after your information and using it properly. The University of Birmingham will keep identifiable information about you for up to 25 years after the study has finished.

Your rights to access, change, or move your information is limited, as we need to manage your information in specific ways in order for the research to be reliable and accurate. If you withdraw from the study, we will keep the information about you that we have already obtained. To safeguard your rights, we will use the minimum personally-identifiable information possible.

You can find out more about how we use your information in our Privacy Policy on our website (www.birmingham.ac.uk/crctu).

The NHS via your hospital(s) will collect information from your medical records for this research study in accordance with our instructions. Your hospital(s) will use your name, NHS number and contact details to contact you about the research study, and make sure that relevant information about the study is recorded for your care, and to oversee the quality of the study.

The NHS will keep identifiable information about you from this study for up to 25 years after the study has finished.

For peer review only - http://bmjopen.bmj.com/site/about/guidelines.xhtml

Involvement of the General Practitioner/Family Doctor (GP)

As this trial requires you to take a drug which may cause side-effects, it is important that your General Practitioner (GP) is informed that you are taking part in the trial. With your permission your trial doctor will notify your GP that you intend to participate in the trial. In addition, we may ask them to provide information on your progress. If we do need to contact your GP for any follow-up information, we will need to use your full name in our correspondence.

For peer review only

For peer review only - http://bmjopen.bmj.com/site/about/guidelines.xhtml

SeluDex

Further information and contact details

If you have any further questions or concerns about your disease or this clinical trial please feel free to discuss them with the doctors and nurses looking after you before deciding to take part in the trial or at any time during the trial. Before you make a decision, you may want to discuss the trial with your family and friends and with your family doctor.

You may also find it helpful to contact the following organisations:

Cancer Research UK

An information service about cancer and cancer research studies hosted by Cancer Research UK

www.cancerresearchuk.org/about-cancer/

Tel: 0808 800 40 40

Macmillan Cancer Support

An independent patient advisory group

Macmillan Cancer Support, 89 Albert Embankment, London, SE1 7UQ, UK

www.macmillan.org.uk

Tel: 0808 808 0000

Children's Cancer and Leukaemia Group

A children's cancer charity for those involved in the treatment and care of children with cancer

CCLGroup, Clinical Sciences Building, Leicester Royal Infirmary, Leicester LE2 7LX

http://www.cclg.org.uk/

Tel: 0116 252 5858

Please take as much time as you need to make a decision and then let your doctor know what you have decided so that your treatment can be arranged.

If you have any further questions, you are very welcome to contact the trial doctor who is leading this trial, whose contact details are provided below:

Local Investigator: …………………………………………………………………...

Trial Nurse: …………………………………………………………………...

Trial Coordinator: …………………………………………………………………...

Emergency Contact Number: …………………………………………………………………...

Thank you for reading this information sheet. If you decide to take part you will be given a copy of this information sheet and your signed consent form.

For peer review only - http://bmjopen.bmj.com/site/about/guidelines.xhtml

Assessments at Each Visit

	Prior to start of treatment	Cycle 1	Cycle 2	Day 1 of each following cycle	End of Treatment	28 days follow up
**Laboratory Tests**						
Blood tests	✓	✓ (Days 1, 8, 15, 22 & 28)	✓ (Days 1 & 15)	✓	✓	✓
Urine sample	✓	✓ (Days 1, 8, 15 & 22)	✓ (Day 1)	✓	✓	✓
Pregnancy test [a]	✓		✓	✓	✓	✓
**Disease Assessment**						
Bone marrow testing/MRD Sample	✓	✓ (Day 28)		✓ (Cycle 4 & 6 only)	✓	
Lumbar puncture	✓	✓ (Day 28 if CNS positive)				
**Treatment**						
Selumetinib		✓ (Day 1 and continuously from day 4)				
Dexamethasone		✓ (Day 2-4, 8-11, 15-18 & 22-25)	✓ (Day 1-4)	✓ (Day 1-5)		
Preventative Antibiotics		Throughout treatment				
**Research Samples**						
Blood samples		✓ (Day 1 & 4)	✓ (Day 1)		✓	
**Other Assessments and Activities**						
Medical history	✓					
Physical examination	✓	✓ (Day 1, 8, 15 & 22)	✓ (Day 1 & 15)	✓	✓	✓
Performance Status	✓	✓ (Day 1, 8, 15 & 22)	✓ (Day 1)	✓	✓	✓
Vital signs (incl. weight)	✓	✓ (Day 1, 8, 15 & 22)	✓ (Day 1)	✓	✓	✓
Growth Chart (Height) – 16-18yrs only	✓			✓ (Cycle 4 only)	✓	✓
ECG	✓	✓ (Day 1)	✓ (Day 1)	✓	✓	✓
ECHO	✓	✓ (Day 28)		Additional tests may be necessary if your doctor is concerned about side effects		
Eye exam	✓	Additional tests may be necessary if your doctor is concerned about side effects				
Medication/side effects monitoring	✓ (Done at every clinic visit from screening through to 28-day follow up visit)					

[a] Female participants of childbearing potential only

For peer review only - http://bmjopen.bmj.com/site/about/guidelines.xhtml

SeluDex

Supplementary Appendix 4

SeluDex Trial Schedule of Events

	**Screening**[1] (Within 14 days of Trial Entry)	**C1/Day 1**[2]	**C1/Day 2**	**C1/Day 4**	**C1/Day 8**[3] (± 2 days)	**C1/Day 15**[3] (± 2 days)	**C1/Day 22**[3] (± 2days)	**C1/Day 28**[4] (- 3 days during Phase II only)	**C2/Day 1**[5]	**C2/Day 15**[3] (± 2 days)	**C3/Day 1**[6] (- 2 days)	**C4/Day 1**[6,7] (- 2 days)	**C5/Day 1**[6] (- 2 days)	**C6/Day 1**[6,7] (- 2 days)	**End of Treat-ment Visit**[8] (+ 14 days)	**28 day Follow Up Visit**[9] (+ 2 days)
Informed Consent[a]	x															
RAS-pathway mutation testing[b]	x*														x	
Medical history[c]	x															
Performance Status (ECOG, Lansky, or Karnofsky scale)	x	x			x	x	x		x (- 1 day)		x	x	x	x		x
Vital signs[d]	x	x			x	x	x		x (- 1 day)		x	x	x	x	x	x
Weight (kg)	x	x (-3 days)			x	x	x		x (- 1 day)		x	x	x	x	x	x
Body Surface Area	x	x (-3 days)							x (- 1 day)		x	x	x	x		
Physical examination[e]	x	x			x	x	x		x (- 1 day)	x	x	x	x	x		x
Serum chemistry[f]	x	x (-3 days)			x	x	x		x (- 1 day)	x	x	x	x	x		x
Full blood count & peripheral blood blast count[g]	x	x (-3 days)			x	x	x	x		x	x	x	x	x	x	x
ECG[h]	x	x							x (- 1 day)		x	x	x	x		x
Urinalysis (dipstick)	x	x			x	x	x		x (- 1 day)		x	x	x	x		x
Height (cm)	x															
Bone marrow aspirate[i]	x							x				x (- 3 days)		x (- 3 days)	x	
Cytogenetic analysis & Immunophenotyping	x															
MRD sample [j]	x							x				x (- 3 days)		x (- 3 days)	x	
Lumbar puncture[k]	x							x								
Serological studies for HBV, HCV, HIV	x															
Pregnancy test[l]	x (-7 days)								x (-1 day)		x	x	x	x	x	x
ECHO[m]	x							x (- 3 days)								
Ophthalmological exam[n]	x															
Adverse events		x	x	x	x	x	x	x	x	x	x	x	x	x	x	x
Concomitant medications	x	x	x	x	x	x	x	x	x	x	x	x	x	x	x	x
Growth Chart (Group P only) [o]	x											x			x	x
Pharmacokinetic samples (blood) [p]		x	x	x					x							

For peer review only

For peer review only - http://bmjopen.bmj.com/site/about/guidelines.xhtml

SeluDex

	Screening[1] (Within 14 days of Trial Entry)	C1/Day 1[2]	C1/Day 2	C1/Day 4	C1/Day 8[3] (± 2 days)	C1/Day 15[3] (± 2 days)	C1/Day 22[3] (± 2days)	C1/Day 28[4] (- 3 days during Phase II only)	C2/Day 1[5]	C2/Day 15[3] (± 2 days)	C3/Day 1[6] (- 2 days)	C4/Day 1[6,7] (- 2 days)	C5/Day 1[6] (- 2 days)	C6/Day 1[6,7] (- 2 days)	End of Treat-ment Visit[8] (+ 14 days)	28 day Follow Up Visit[9] (+ 2 days)
Pharmacodynamic samples - RNA & Fixed (blood)[q]		x	x	x											x	
Patient Diary (dispense and review as appropriate)[r]		x							x		x	x	x	x		
Selumetinib dispensing		x							x		x	x	x	x		
Dexamethasone dispensing			x						x		x	x	x	x		
Fluoroquinolone prophylaxis dispensing		x							x							
Co-trimoxazole prophylaxis dispensing		x							x		x	x	x	x		

Initially trial treatment is for 6 cycles (approximately 6 months) however following this participants who are considered to be receiving clinical benefit can remain on trial and have continued access to selumetinib whilst the trial is open following approval from AstraZeneca. The schedule of assessments for treatment continuation should follow cycles 3-6.

[1] Any assessments done as standard care do not require informed consent and may be provided as screening data if conducted within 14 days of trial entry unless otherwise stated

[2] Weight, Body Surface Area, Serum Chemistry, FBC and peripheral blood blast count may be performed within the 3 days prior to cycle 1 day 1

[3] Visit may occur within the 2 days prior to or after the scheduled visit date

[4] During Phase I bone marrow aspirate and all associated assessments at this visit must occur on cycle 1 day 28 of treatment except for the ECHO which may be performed within the 3 days prior to the scheduled visit date. During Phase II this visit and all associated assessments may occur within the 3 days prior to the scheduled visit date

[5] Performance status, vital signs, weight, BSA, physical examination, serum chemistry, FBC, ECG and urinalysis (dipstick) may be performed within the 1 day prior to the scheduled visit date

[6] Visit may occur within the 2 days prior to the scheduled visit date

[7] Bone marrow aspirate and MRD sample may be performed within the 3 days prior to the scheduled visit date

[8] The End of Treatment assessment visit should be performed within 14 days of the patient's last administration of selumetinib. Should the patient discontinue following a recent bone marrow aspirate visit then a repeat bone marrow aspirate for end of treatment is not required at the discretion of the investigator

[9] Patients will be followed up for 28 days after the last dose of selumetinib, with a clinic visit taking place on day 28. Visit may occur within the 2 days after the scheduled visit date

* RAS-pathway mutation result date does not have to be within 14 days of trial entry but must be confirmed using a sample from the current relapse/disease

[a] Written informed consent must be received before any trial specific procedures occur

[b] Sample from current relapse/disease must confirm presence of a RAS-pathway activating mutation - NRAS, KRAS, FLT3, PTPN11, cCBL, NF1, BRAF, IKZF2, IKZF3, IL7Rα or JAK1. Results from trial office approved local molecular testing are accepted for all of these mutations. Testing for NRAS, KRAS, FLT3, PTPN11 and cCBL mutations can be done centrally by Northern Genetics Services (Previously NewGene Limited) in Newcastle, UK – refer to the laboratory manual for further information. Optional end of treatment sample to be collected from non-responders and stored at sites with local biobank facilities and appropriate ethical approval using local consent to assess clonal evolution of RAS-pathway wildtype ALL

[c] Medical history – including demographics, prior treatment, allergy history, discuss contraception

For peer review only - http://bmjopen.bmj.com/site/about/guidelines.xhtml

SeluDex

[d] Blood pressure, pulse measurement, temperature, and respiratory rate to be performed with the patient sitting for 5 minutes prior to the evaluation

[e] Physical examination includes complete review of systems, physical examination of pertinent organ systems, and neurological exam

[f] Includes: Glucose, LDH and creatine kinase (CK) at screening only. Creatinine, Sodium, Potassium, Total Calcium, Total Protein, Phosphate, Magnesium, Urea, Total Bilirubin, AST or ALT ALP and albumin at all timepoints

[g] Full Blood Count with differential count and peripheral blood blast count as an additional request test at all timepoints

[h] Repeat ECG as clinically indicated

[i] Bone marrow aspirate at screening (peripheral blood can be used in the case of a dry tap if peripheral WCC > $50x10^9$/L – screening only); cycle 1 day 28, cycle 4 day 1 and cycle 6 day 1. For any patients who continue treatment after cycle 6, a bone marrow aspirate should be performed every other cycle.

[j] An MRD sample will be collected from the bone marrow aspirate at screening for all patients. At subsequent timepoints an MRD sample should be collected each time a BM aspirate is performed for patients who have achieved at least a CRi and where a marker was identified at screening. Group A: MRD samples will be sent to UCL for analysis. Group P: MRD will be analysed as part of standard of care

[k] A Lumbar puncture to assess CNS status should be done at Screening for all patients and at cycle 1 day 28 for CNS positive patients only

[l] Women of childbearing potential will require a negative pregnancy test (serum or urine) prior to registration and within seven days prior to study drug administration, then monthly

[m] An ECHO should be performed at screening. Repeat on cycle 1 day 28 (or within the 3 days prior) and as clinically indicated. (Group A: LVEF, LVEDV & LVESV, Group P: SF)

[n] Repeat as clinically indicated

[o] Group P only. Body height (standing or lying length where age-appropriate) will be measured. Where possible, a stadiometer should be used. Where this may be difficult in particularly young children, standard measurement methods may be used.

[p] Pharmacokinetic samples at the following time points:

- Cycle 1 day 1: pre first selumetinib dose, then at 30 minutes, 1 hour, 2 hours, 4 hours, 6 hours and 24 hours after the first dose (pre cycle 1 day 2 dexamethasone dose)
- Cycle 1 day 4: pre first selumetinib/dexamethasone dose, then at 30 minutes, 1 hour, 2 hours, 4 hours, 6 hours after the first dose
- Cycle 2 day 1: pre first selumetinib/dexamethasone dose, then at 30 minutes, 1 hour, 2 hours, 4 hours, 6 hours after the first dose

[q] Pharmacodynamic blood samples at the following time points (only if WCC >$10x10^9$/l). PD-RNA samples - all sites. PD-Fixed samples – if facilities available at site as agreed at initiation:

- Cycle 1 day 1: pre first selumetinib dose, then at 1 hour, 4 hours, 6 hours and 24 hours after the first dose
- Cycle 1 day 4: pre first selumetinib/dexamethasone dose, then at 1 hour, 4 hours, 6 hours after the first dose
- End of Treatment visit (one sample only)

[r] All patients/parents/legal guardians will be required to complete a diary, which must be returned to the clinic for checking at each visit. The research nurse should complete the relevant sections including recording the AM and PM doses and the dates they are to be taken on the diary. Patients/parents/legal guardians should be instructed to record daily time of administration of the study drugs in the diary. If a dose is missed, the reason must be noted in the diary by the patient/parent/legal guardian. The patient diary will then be reviewed by the research nurse at the end of each cycle. Patients/parents/legal guardians should be advised to return any unused IMP in the original bottles, in addition to returning any empty bottles.

For peer review only - http://bmjopen.bmj.com/site/about/guidelines.xhtml
For peer review only

SeluDex

Supplementary Appendix 5

Adverse Event

Any untoward medical occurrence in a patient or clinical trial subject administered a medicinal product and which does not necessarily have a causal relationship with this treatment.

Comment:

An AE can therefore be any unfavourable and unintended sign (including abnormal laboratory findings), symptom or disease temporally associated with the use of an investigational medicinal product, whether or not related to the investigational medicinal product (IMP).

Adverse Reaction

All untoward and unintended responses to an IMP related to any dose administered.

Comment:

An AE judged by either the reporting Investigator or Sponsor as having causal relationship to the IMP qualifies as an AR. The expression reasonable causal relationship means to convey in general that there is evidence or argument to suggest a causal relationship.

Serious Adverse Event

Any untoward medical occurrence or effect that at any dose:

Results in death

Is life threatening*

Requires hospitalisation** or prolongation of existing inpatients' hospitalisation

Results in persistent or significant disability or incapacity

Is a congenital anomaly/birth defect

Or is otherwise considered medically significant by the Investigator***

Comments:

The term severe is often used to describe the intensity (severity) of a specific event. This is not the same as serious, which is based on patients/event outcome or action criteria.

* Life threatening in the definition of an SAE refers to an event in which the patient was at risk of death at the time of the event; it does not refer to an event that hypothetically might have caused death if it were more severe.

**Hospitalisation is defined as an unplanned, formal inpatient admission, even if the hospitalisation is a precautionary measure for continued observation. Thus, hospitalisation for protocol treatment (e.g., line insertion), elective procedures (unless brought forward because of worsening symptoms) or for social reasons (e.g., respite care) are not regarded as an SAE.

For peer review only

*** Medical judgment should be exercised in deciding whether an AE is serious in other situations. Important AEs that are not immediately life threatening or do not result in death or hospitalisation but may jeopardise the subject or may require intervention to prevent one of the other outcomes listed in the definition above, should be considered serious.

Serious Adverse Reaction

An Adverse Reaction which also meets the definition of a Serious Adverse Event.

Suspected Unexpected Serious Adverse Reaction

A SAR that is unexpected i.e., the nature, or severity of the event is not consistent with the applicable product information.

A SUSAR should meet the definition of an AR, UAR and SAR.

Unexpected Adverse Reaction

An AR, the nature or severity of which is not consistent with the applicable product information (e.g., Investigator Brochure for an unapproved IMP or (compendium of) Summary of Product Characteristics (SPC) for a licensed product).

When the outcome of an AR is not consistent with the applicable product information the AR should be considered unexpected.

For peer review only

SPIRIT
STANDARD PROTOCOL ITEMS: RECOMMENDATIONS FOR INTERVENTIONAL TRIALS

SPIRIT 2013 Checklist: Recommended items to address in a clinical trial protocol and related documents*

Section/item	Item No	Description
**Administrative information**		
Title	1	Descriptive title identifying the study design, population, interventions, and, if applicable, trial acronym<br><br>Page 1; lines 1-4
Trial registration	2a	Trial identifier and registry name. If not yet registered, name of intended registry<br><br>Page 2; lines 57-58
	2b	All items from the World Health Organization Trial Registration Data Set<br><br>Supplementary Appendix 1
Protocol version	3	Date and version identifier<br><br>Page 12; line 442
Funding	4	Sources and types of financial, material, and other support<br><br>Page 13; lines 466-472
Roles and responsibilities	5a	Names, affiliations, and roles of protocol contributors<br><br>Names and affiliations on page 1; lines 5-26<br>Names and role on page 12; lines 454-464
	5b	Name and contact information for the trial sponsor<br><br>Page 10; lines 359-360
	5c	Role of study sponsor and funders, if any, in study design; collection, management, analysis, and interpretation of data; writing of the report; and the decision to submit the report for publication, including whether they will have ultimate authority over any of these activities<br><br>Page 13; lines 474-476
	5d	Composition, roles, and responsibilities of the coordinating centre, steering committee, endpoint adjudication committee, data management team, and other individuals or groups overseeing the trial, if applicable (see Item 21a for data monitoring committee)

For peer review only

		Page 10; lines 359-375
**Introduction**		
Background and rationale	6a	Description of research question and justification for undertaking the trial, including summary of relevant studies (published and unpublished) examining benefits and harms for each intervention<br>Page 3; lines 69-95
	6b	Explanation for choice of comparators<br>Page 3; line 102
Objectives	7	Specific objectives or hypotheses<br>Page 3; lines 96-98
Trial design	8	Description of trial design including type of trial (eg, parallel group, crossover, factorial, single group), allocation ratio, and framework (eg, superiority, equivalence, noninferiority, exploratory)<br>Page 3; lines 101-104
**Methods: Participants, interventions, and outcomes**		
Study setting	9	Description of study settings (eg, community clinic, academic hospital) and list of countries where data will be collected. Reference to where list of study sites can be obtained<br>Page 3; lines 103-104 and Page 11; line 338
Eligibility criteria	10	Inclusion and exclusion criteria for participants. If applicable, eligibility criteria for study centres and individuals who will perform the interventions (eg, surgeons, psychotherapists)<br>Page 4; lines 116-128
Interventions	11a	Interventions for each group with sufficient detail to allow replication, including how and when they will be administered<br>Page 4; lines 141-168
	11b	Criteria for discontinuing or modifying allocated interventions for a given trial participant (eg, drug dose change in response to harms, participant request, or improving/worsening disease)<br>Page 5; lines 181-197
	11c	Strategies to improve adherence to intervention protocols, and any procedures for monitoring adherence (eg, drug tablet return, laboratory tests)<br>n/a

For peer review only - http://bmjopen.bmj.com/site/about/guidelines.xhtml

	11d	Relevant concomitant care and interventions that are permitted or prohibited during the trial<br><br>Page 6; lines 199-201
Outcomes	12	Primary, secondary, and other outcomes, including the specific measurement variable (eg, systolic blood pressure), analysis metric (eg, change from baseline, final value, time to event), method of aggregation (eg, median, proportion), and time point for each outcome. Explanation of the clinical relevance of chosen efficacy and harm outcomes is strongly recommended<br><br>Page 6; lines 203-241
Participant timeline	13	Time schedule of enrolment, interventions (including any run-ins and washouts), assessments, and visits for participants. A schematic diagram is highly recommended (see Figure)<br><br>Supplementary Appendix 4
Sample size	14	Estimated number of participants needed to achieve study objectives and how it was determined, including clinical and statistical assumptions supporting any sample size calculations<br><br>Page 8; lines 262-313
Recruitment	15	Strategies for achieving adequate participant enrolment to reach target sample size<br><br>n/a

Methods: Assignment of interventions (for controlled trials)

Allocation:

Sequence generation	16a	Method of generating the allocation sequence (eg, computer-generated random numbers), and list of any factors for stratification. To reduce predictability of a random sequence, details of any planned restriction (eg, blocking) should be provided in a separate document that is unavailable to those who enrol participants or assign interventions<br><br>n/a
Allocation concealment mechanism	16b	Mechanism of implementing the allocation sequence (eg, central telephone; sequentially numbered, opaque, sealed envelopes), describing any steps to conceal the sequence until interventions are assigned<br><br>n/a
Implementation	16c	Who will generate the allocation sequence, who will enrol participants, and who will assign participants to interventions

For peer review only - http://bmjopen.bmj.com/site/about/guidelines.xhtml

Blinding (masking)	17a	Who will be blinded after assignment to interventions (eg, trial participants, care providers, outcome assessors, data analysts), and how<br><br>n/a
	17b	If blinded, circumstances under which unblinding is permissible, and procedure for revealing a participant's allocated intervention during the trial<br><br>n/a

Methods: Data collection, management, and analysis

Data collection methods	18a	Plans for assessment and collection of outcome, baseline, and other trial data, including any related processes to promote data quality (eg, duplicate measurements, training of assessors) and a description of study instruments (eg, questionnaires, laboratory tests) along with their reliability and validity, if known. Reference to where data collection forms can be found, if not in the protocol<br><br>Page 10; lines 339-357
	18b	Plans to promote participant retention and complete follow-up, including list of any outcome data to be collected for participants who discontinue or deviate from intervention protocols<br><br>n/a
Data management	19	Plans for data entry, coding, security, and storage, including any related processes to promote data quality (eg, double data entry; range checks for data values). Reference to where details of data management procedures can be found, if not in the protocol<br><br>Page 10; lines 339-357
Statistical methods	20a	Statistical methods for analysing primary and secondary outcomes. Reference to where other details of the statistical analysis plan can be found, if not in the protocol<br><br>Page 6; lines 212-242
	20b	Methods for any additional analyses (eg, subgroup and adjusted analyses)<br><br>Page 6; lines 234-236
	20c	Definition of analysis population relating to protocol non-adherence (eg, as randomised analysis), and any statistical methods to handle missing data (eg, multiple imputation)<br><br>Page 8; lines 263-269

For peer review only - http://bmjopen.bmj.com/site/about/guidelines.xhtml

**Methods: Monitoring**		
Data monitoring	21a	Composition of data monitoring committee (DMC); summary of its role and reporting structure; statement of whether it is independent from the sponsor and competing interests; and reference to where further details about its charter can be found, if not in the protocol. Alternatively, an explanation of why a DMC is not needed<br><br>Page 10; lines 360-376
	21b	Description of any interim analyses and stopping guidelines, including who will have access to these interim results and make the final decision to terminate the trial<br><br>Page 9; line 314
Harms	22	Plans for collecting, assessing, reporting, and managing solicited and spontaneously reported adverse events and other unintended effects of trial interventions or trial conduct<br><br>Page 10; lines 372-374
Auditing	23	Frequency and procedures for auditing trial conduct, if any, and whether the process will be independent from investigators and the sponsor<br><br>n/a
**Ethics and dissemination**		
Research ethics approval	24	Plans for seeking research ethics committee/institutional review board (REC/IRB) approval<br><br>Page 12; lines 439-443
Protocol amendments	25	Plans for communicating important protocol modifications (eg, changes to eligibility criteria, outcomes, analyses) to relevant parties (eg, investigators, REC/IRBs, trial participants, trial registries, journals, regulators)<br><br>n/a
Consent or assent	26a	Who will obtain informed consent or assent from potential trial participants or authorised surrogates, and how (see Item 32)<br><br>Page 4; line 131-136
	26b	Additional consent provisions for collection and use of participant data and biological specimens in ancillary studies, if applicable<br><br>n/a

For peer review only

For peer review only - http://bmjopen.bmj.com/site/about/guidelines.xhtml

Confidentiality	27	How personal information about potential and enrolled participants will be collected, shared, and maintained in order to protect confidentiality before, during, and after the trial<br><br>Page 10; lines 378-385
Declaration of interests	28	Financial and other competing interests for principal investigators for the overall trial and each study site<br><br>Page 13; lines 479-480
Access to data	29	Statement of who will have access to the final trial dataset, and disclosure of contractual agreements that limit such access for investigators<br><br>Page 10; lines 353-358
Ancillary and post-trial care	30	Provisions, if any, for ancillary and post-trial care, and for compensation to those who suffer harm from trial participation<br><br>n/a
Dissemination policy	31a	Plans for investigators and sponsor to communicate trial results to participants, healthcare professionals, the public, and other relevant groups (eg, via publication, reporting in results databases, or other data sharing arrangements), including any publication restrictions<br><br>Page 12; lines 444-447
	31b	Authorship eligibility guidelines and any intended use of professional writers<br><br>Page 12; lines 446-447
	31c	Plans, if any, for granting public access to the full protocol, participant-level dataset, and statistical code<br><br>n/a
**Appendices**		
Informed consent materials	32	Model consent form and other related documentation given to participants and authorised surrogates<br><br>Appendices 2 and 3
Biological specimens	33	Plans for collection, laboratory evaluation, and storage of biological specimens for genetic or molecular analysis in the current trial and for future use in ancillary studies, if applicable<br><br>n/a

*It is strongly recommended that this checklist be read in conjunction with the SPIRIT 2013 Explanation & Elaboration for important clarification on the items. Amendments to the protocol should be tracked and dated. The SPIRIT checklist is copyrighted by the SPIRIT

For peer review only

Group under the Creative Commons "Attribution-NonCommercial-NoDerivs 3.0 Unported" license.

For peer review only
