# Supplementary material for: Selumetinib in combination with dexamethasone for the treatment of relapsed/refractory RAS-pathway mutated paediatric and adult acute lymphoblastic leukaemia (SeluDex): study protocol for an international, parallel-group, dose-finding with expansion phase I/II trial
Source: BMJ Open. 2022 Mar 4;12(3):e059872. doi: 10.1136/bmjopen-2021-059872 (PMC8900053; doi:10.1136/bmjopen-2021-059872)
Supplement: Reviewer comments [file bmjopen-2021-059872.reviewer_comments.pdf]

PEER REVIEW HISTORY

BMJ Open publishes all reviews undertaken for accepted manuscripts. Reviewers are asked to complete a checklist review form (**http://bmjopen.bmj.com/site/about/resources/checklist.pdf**) and are provided with free text boxes to elaborate on their assessment. These free text comments are reproduced below.

ARTICLE DETAILS

**TITLE (PROVISIONAL)**	Selumetinib in combination with dexamethasone for the treatment of relapsed/refractory RAS-pathway mutated paediatric and adult acute lymphoblastic leukaemia (SeluDex): study protocol for an international, parallel-group, dose-finding with expansion phase I/II trial
**AUTHORS**	Menne, Tobias; Slade, Daniel; Savage, Joshua; Johnson, Sarah; Irving, Julie; Kearns, Pamela; Plummer, Ruth; Shenton, Geoff; Veal, Gareth J; Vormoor, Britta; Vormoor, Josef; Billingham, Lucinda

This article was not externally reviewed at BMJ Open. Protocols that have been independently assessed prior to submission to BMJ Open are usually fast-tracked to publication on the grounds that further substantial changes will not be possible. This independent assessment will usually be external, independent review for both a substantial grant award from a non-commercial or government funder and ethics approval.
